# Stoichiometric and irreversible cysteine-selective protein modification using carbonylacrylic reagents

**DOI:** 10.1038/ncomms13128

**Published:** 2016-10-26

**Authors:** Barbara Bernardim, Pedro M.S.D. Cal, Maria J. Matos, Bruno L. Oliveira, Nuria Martínez-Sáez, Inês S. Albuquerque, Elizabeth Perkins, Francisco Corzana, Antonio C.B. Burtoloso, Gonzalo Jiménez-Osés, Gonçalo J. L. Bernardes

**Affiliations:** 1 Department of Chemistry, University of Cambridge, Lensfield Road, CB2 1EW Cambridge UK; 2 Instituto de Química de São Carlos, Universidade de São Paulo, São Carlos, São Paulo CEP 13560-970, Brazil; 3 Instituto de Medicina Molecular, Faculdade de Medicina, Universidade de Lisboa, Avenida Professor Egas Moniz, 1649-028 Lisboa, Portugal; 4 Albumedix Ltd, Castle Court, 59 Castle Boulevard, Nottingham NG7 1FD, UK; 5 Departamento de Química, Universidad de La Rioja, Centro de Investigacioón en Síntesis Química, 26006 Logroño, Spain; 6 Institute of Biocomputation and Physics of Complex Systems (BIFI), University of Zaragoza, BIFI-IQFR (CSIC), 50018 Zaragoza, Spain

## Abstract

Maleimides remain the reagents of choice for the preparation of therapeutic and imaging protein conjugates despite the known instability of the resulting products that undergo thiol-exchange reactions *in vivo*. Here we present the rational design of carbonylacrylic reagents for chemoselective cysteine bioconjugation. These reagents undergo rapid thiol Michael-addition under biocompatible conditions in stoichiometric amounts. When using carbonylacrylic reagents equipped with PEG or fluorophore moieties, this method enables access to protein and antibody conjugates precisely modified at pre-determined sites. Importantly, the conjugates formed are resistant to degradation in plasma and are biologically functional, as demonstrated by the selective imaging and detection of apoptotic and HER2+ cells, respectively. The straightforward preparation, stoichiometric use and exquisite cysteine selectivity of the carbonylacrylic reagents combined with the stability of the products and the availability of biologically relevant cysteine-tagged proteins make this method suitable for the routine preparation of chemically defined conjugates for *in vivo* applications.

## Abstract (web summary)

Current cysteine bioconjugation strategies for protein-drug conjugates synthesis often yield heterogeneous and poorly stable products. Here, the authors use carbonylacrylic derivatives to selectively modify cysteine residues and synthesize biologically functional antibody conjugates highly stable in plasma.

## Graphical abstract

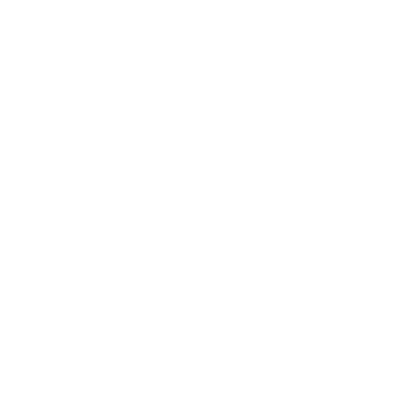

Protein bioconjugation has a crucial role in the development of novel biologically active protein conjugates for applications in biology and medicine^1^,^2^,^3^. The chemoselectivity and mildness of the process are key to precisely install modifications at pre-determined sites without disturbing the structure, function and activity of the protein^4^,^5^,^6^. Reactivity, accessibility and abundance of a particular amino acid side chain are key aspects required to achieve selective modification of one certain residue over all other proteinogenic amino acids. Among these, cysteine (Cys) remains the amino acid of choice due to its low abundance and the high nucleophilicity of the sulfhydryl side chain^7^. Indeed, most protein conjugates used in the clinic, for example antibody-drug conjugates (ADCs)^2^,^8^, are prepared using Cys bioconjugation methods.

Several alkylation reagents and Michael acceptors have been and continue to be reported for Cys bioconjugation^7^,^9^. More recently, selective arylation of Cys has also been demonstrated as a viable strategy for Cys selective bioconjugation. Cys arylation has been achieved by reacting perfluoroaryl reagents with a sequence specific (Phe-Cys-Pro-Phe, termed ‘π-clamp')^10^ that was genetically encoded into a protein or by using organometallic palladium reagents^11^.

Despite the developments in the field of Cys bioconjugation, maleimides are still the most commonly used reagents, mainly due to fast aqueous reaction kinetics, easy synthesis of modified maleimide reagents and the fact that does not require extensive genetic engineering of the protein target. For instance, the Food and Drug Administration approved ADC—Brentuximab vedotin^12^—is prepared by conjugation of a maleimide linker-drug molecule to Cys residues on an antibody. However, it is now well-documented that the thio-succinimidyl conjugates formed undergo rapid exchange reactions with thiols present in plasma leading to the release of the maleimide^13^,^14^,^15^. In the case of ADCs, this can lead to toxicity, as the product of the thiol-exchange reaction is a highly potent cytotoxic drug. Thus, there is a pressing need for the development of methods to ready build protein and antibody conjugates in a manner that allows for site-selective and irreversible installation of probes and drugs at specific sites within their sequence.

In the specific case of maleimides, strategies have been pursued to enhance the natively slow hydrolysis kinetics in order to generate a stable ring-opened maleimide. This has been achieved either by genetic engineering of the sequence adjacent to Cys^13^ or by chemical introduction of a basic amino group on the maleimide structure^16^,^17^,^18^. These approaches lead to conjugates that are more stable *in vivo*, but require extensive sequence engineering, use reagents that result in a less efficient conjugation and often result in heterogenous mixtures of hydrolysed versus non-hydrolysed conjugates. Replacement of the endocyclic olefin by an exocyclic one has also been shown to reduce thiol-exchange reactions^19^. However, the slower kinetics of the reaction of exocyclic maleimides with Cys requires a larger excess of reagents to achieve useful conversions in proteins. Furthermore, an open form of a maleimide has been shown to be a useful scaffold when conjugated to a coumarin for fast thiol-quantification glutathione reductase^20^.

In an attempt to provide an optimal Cys-bioconjugation method, we explored simple carbonylacrylic derivatives to identify those that were highly reactive for Cys in aqueous conditions at near neutral pH and afforded products resistant to degradation that might be used for *in vivo* therapeutic and imaging applications.

## Results

### Design of carbonylacrylic reagents for Cys modification

**Figure 1:**
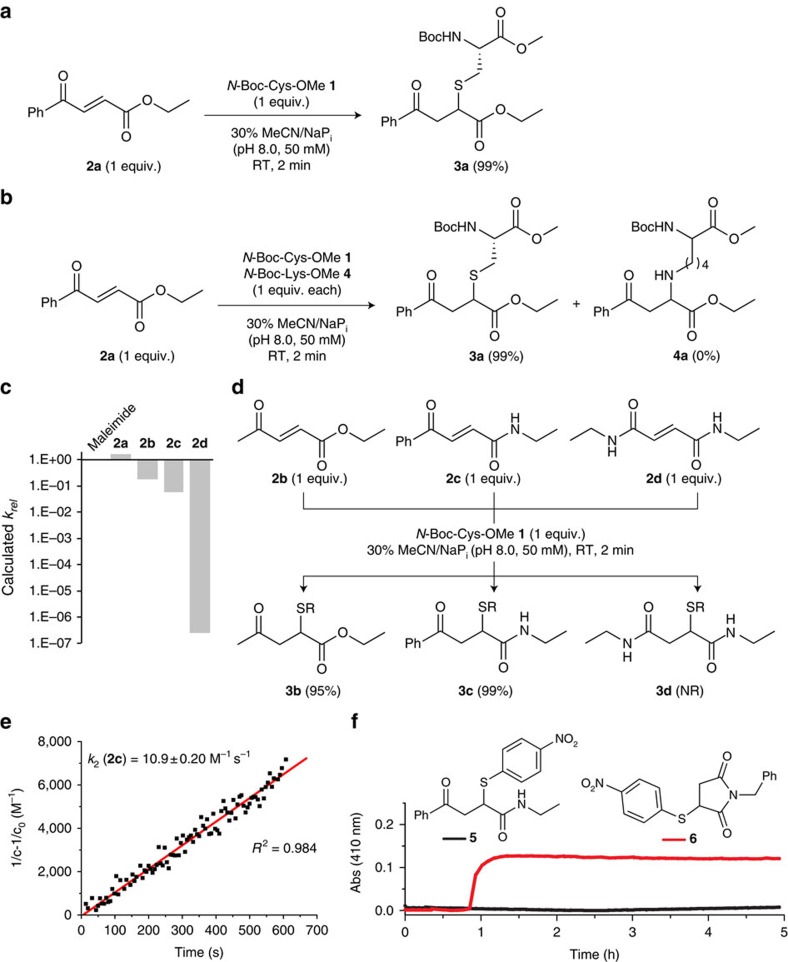
Identification of a suitable carbonylacrylic derivative for reaction with Cys. (**a**) Reaction of **2a** with *N*-Boc-Cys-OMe **1** provides the thioether product **3a** in 99% yield after 2 min. (**b**) Reaction of **2a** in an equimolar mixture of **1** and *N*-Boc-Lys-OMe **4** shows complete Cys chemoselectivity. (**c**) Relative reaction rates at 25 °C (*k*_*rel*_) calculated for the conjugate addition of methyl thiolate to maleimide and 3-carbonylacrylic acid derivatives **2a**–**d**. (**d**) Reaction of derivatives **2b**–**d** with **1**. (**e**) Experimental determination of the second-order rate constant for the addition of compound **2c** to **1**. See the Supplementary Methods for additional details and kinetics data for compound **2a**. (**f**) Kinetic assay for the stability of chromogenic thiol adducts **5** and **6** at physiological pH. The absorbance of **5** at 410 nm is constant over time, indicating no release of 4-nitrophenolate. In contrast, for **6**, a steady increase in absorbance over time was observed. It is important to note that although both compounds absorb at 410 nm, we have set the initial absorbance to zero for clarity. The original data for **5** (*A*_0h_=0.45 nm, *A*_5h_=0.45 nm) and for **6** (*A*_0h_=0.23 nm, *A*_5h_=0.35 nm) is presented in the Supplementary Fig. 7. NR, no reaction; RT, room temperature.

We began our study by reacting the protected amino acid Boc-Cys methyl ester **1** with the commercially available carbonylacrylic reagent, 3-benzoylacrylate **2a**. Remarkably, the reaction was complete (99% yield) using stoichiometric amounts of **2a** in less than 2 min at room temperature and could be conducted in 30% MeCN in sodium phosphate buffer (NaP_i_, pH 8.0, 50 mM) in open air (Fig. 1a). Importantly, in a competition experiment performed under the same reaction conditions, Boc-Lysine(Lys) methyl ester **4** remains unreactive and only the Cys adduct is formed, demonstrating well the high chemoselectivity of **2a** towards Cys (Fig. 1b). Considering the structural simplicity of **2a**, we designed a series of variants with the aid of quantum mechanical calculations, balancing reactivity and functionality at both ends of the reacting alkene. Ketones, esters and amides **2b**–**d** were considered aiming to find a highly reactive and extensible reagent that could be used for rapid Cys selective and irreversible protein conjugation. An abbreviated Cys (MeS^−^) was used in the calculations as a model sulfhydryl nucleophile. A quickly evaluable reactivity predictor such as the energy gap (Δ*E*) between the highest occupied molecular orbital of MeS^−^ and the lowest unoccupied molecular orbital of the Michael acceptor, was first used to screen the reactivity of different carbonylacrylic candidates (a smaller highest occupied molecular orbital–lowest unoccupied molecular orbital gap would indicate a higher reaction rate)^21^. Such analysis predicted that *trans*-1,2-disubstituted olefins bearing either a methyl ketone and an ester (**2b,** Δ*E*=4.5 eV) or a phenyl ketone and an amide (**2c**, Δ*E*=4.4 eV) should have a reactivity comparable to that of **2a** (Δ*E*=4.2 eV) and maleimide (Δ*E*=4.3 eV). Further refinement of this descriptor through more elaborate transition state calculations (Supplementary Figs 1 and 2 and Supplementary Table 1 and Supplementary Data 1), confirmed that **2a**, **2b** and **2c** are within the same reactivity window than the reference maleimide (see relative calculated reaction rates, Fig. 1c). Of note, while an amide is tolerated at one side of the double bond, offering great synthetic advantages for bioconjugation, the presence of amides at both ends of the olefin such as in **2d,** (Δ*E*=5.1 eV), reduces its calculated reactivity up to seven orders of magnitude due to its inability to stabilize the negative charge accumulated in the carbonylacrylamide upon thiolate addition. Our experimental results were in accordance with these predictions as carbonylacrylic derivatives **2a–c** gave a complete product formation (Fig. 1a,d) and the reaction proceeded with fast kinetics (*k*_*2*_ (**2a**)=40.2 M^−1^s^−1^ and *k*_*2*_ (**2c**)=10.9 M^−1^s^−1^; Fig. 1e and Supplementary Fig. 3). Finally, a competition experiment followed by ^1^H-NMR against the commonly used *N*-methyl maleimide, showed a similar product distribution indicating a comparable reaction rate for **2a**, **2b** and the maleimide derivative (Supplementary Fig. 4).

Next, we studied and compared the stability of thioether conjugates obtained using the novel carbonylacrylates with those obtained using conventional maleimides. Using a kinetic assay with chromogenic thiol adducts, we demonstrated that **5**, which was prepared from a carbonylacrylic derivative, is fully stable when compared with the thio-succinimidyl derivative **6** at pH 7.4 (Fig. 1f). In addition, reaction of **3c** with the natural thiol nucleophile glutathione (GSH), showed no C–S cleavage for over a week as assessed by NMR and mass spectrometry (Supplementary Figs 5 and 6).

### Reaction optimization for protein modification

**Figure 2:**
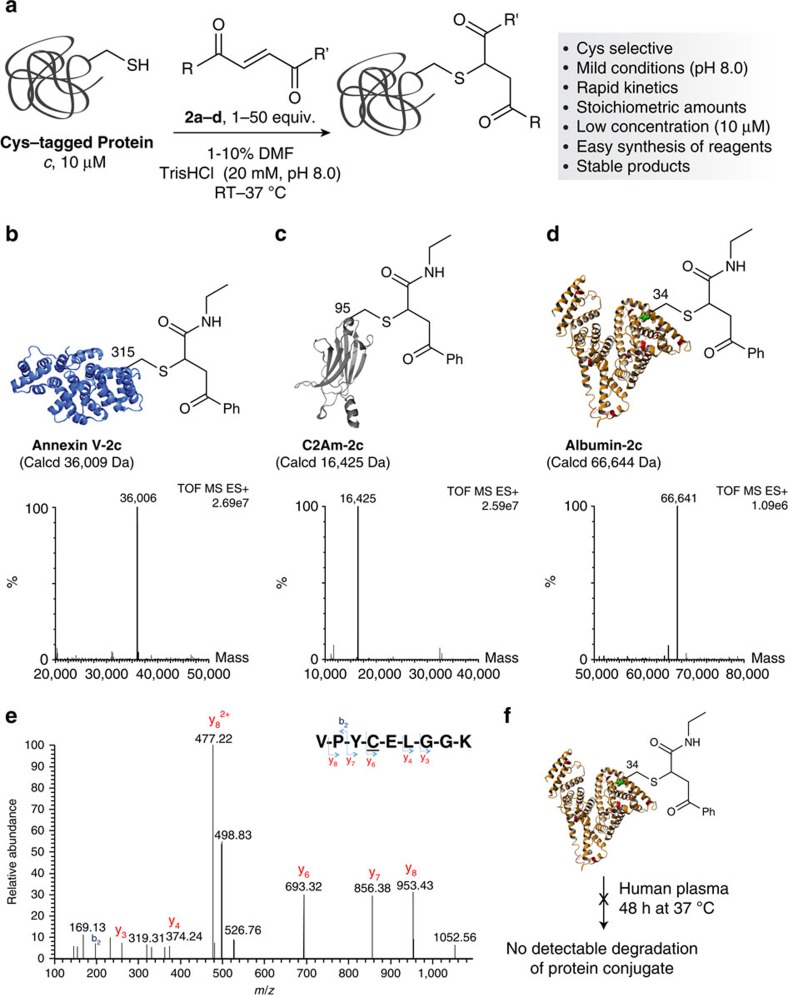
Protein Cys bioconjugation with carbonylacrylic reagent **2c**. (**a**) Schematic of the reaction of Cys-tagged proteins with carbonylacrylic reagents **2a**–**d**. (**b**–**d**) Electrospray ionization–MS spectra of conjugates (**b**) Annexin V-**2c**, (**c**) C2Am-**2c** and (**d**) Albumin-**2c**. (**e**) MS/MS spectrum of the *m/z* 526.75 doubly charged ion of the tryptic peptide VPYCELGGK, containing the modification at the Cys residue of C2Am-**2c**. The generated *C*-terminal y fragment ions (y6–y8) are consistent with the mass of the modification. (**f**) The albumin-**2c** conjugate remains intact when exposed to exogenous (GSH) thiols and in plasma as assessed by LC–MS. See Supplementary Fig. 21. RT, room temperature; DMF, dimethylformamide.

With a new class of irreversible Michael-acceptor reagents that are Cys-selective in hand, we further explored their use for protein bioconjugation. The carbonylacrylic derivatives **2a**–**d** were evaluated for the labelling of Annexin V, a protein used as an apoptosis imaging agent that contains a free, hindered Cys at position 316 (Table 1 and Supplementary Figs 8–12)^22^. Using 25 equivalents of **2a**–**d** in TrisHCl (pH 8.0, 50 mM) for 27 h at 25 °C, we found that only **2c** gave a useful conversion to the corresponding thioether adduct. Both **2a** and **2b,** featuring ester motifs, suffer from partial hydrolysis under the conditions tested. Based on these results, we decided to optimize the reaction conditions using **2c** and found that for Annexin V complete conversion to the product (>95%) can be obtained using 50 equivalents of **2c** after 1 h at 37 °C as confirmed by liquid-chromatography–mass-spectrometry (LC–MS) analysis. In this regard, the total protein content in the ion chromatogram before and after the conjugation reaction is analysed by MS and the protein concentration is analysed after purification of the conjugate by Bradford protein assay, indicating complete conversion to a single product in >95% yield (Fig. 2b and Supplementary Fig. 13). Under identical conditions, pH 8.0 and 50 equivalents, the reaction of Annexin V with a maleimide affords <50% of the expected conjugate with additional modifications on Lys (data not shown).

### Protein scope for Cys-bioconjugation

**Figure 3:**
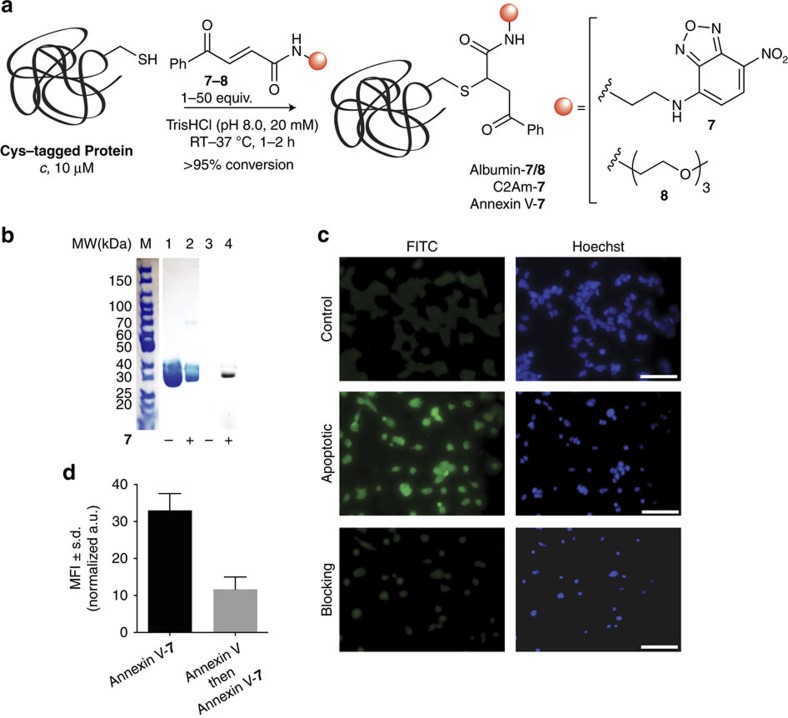
Fluorescent labelling and PEGylation of proteins. (**a**) Scheme of the modification of albumin and Annexin V with fluorescent **7** or PEG **8** functionalized carbonylacrylic reagents. See Supplementary Methods for full synthetic details. (**b**) Treatment of Annexin V with **7** afforded a fluorescent protein conjugate, as detected by SDS–polyacrylamide gel electrophoresis gel. Lanes 1 and 2, coommassie staining. Lanes 3 and 4, fluorescence. (**c**) Fluorescence images of non-apoptotic (control) and apoptotic HEK293 cells after labelling with Annexin V-**7**. Blocking studies were performed by pre-incubation of apoptotic cells with a 25 × excess of non-fluorescent Annexin V before incubation with Annexin V-**7**. (**d**) The mean fluorescent intensity (MFI) showed a significant decrease of the binding of Annexin V-**7** after blocking of the apoptotic receptors. Apoptotic cells appear green with the FITC filter, while the nuclei are stained blue with the Hoechst dye. Scale bar, 100 μm. s.d., standard deviation; a.u., arbitrary units; RT, room temperature.

The scope of method was then studied on different proteins (Fig. 3a) including an engineered variant of the C2A domain of Synaptotagmin-I (C2Am), which is another apoptosis imaging agent^23^, as well as a recombinant human albumin—Recombumin (Albumedix Ltd), which has been used for example in drug-delivery applications (Supplementary Figs 14–17; ref. 24). Both proteins have surface exposed Cys residues and full conversion to the corresponding thioether adducts could be obtained when reacted with **2c** (Fig. 2c,d). Much to our delight, we found that for albumin a single equivalent of **2c** is sufficient to achieve complete conversion to the product, which demonstrates the high efficiency of our method (Supplementary Figs 16 and 17). While for certain less reactive Cys residues a slight excess of reagent may be necessary (25–50 equivalents), stoichiometric Cys selective modification may be attained depending on the local geometry and chemical environment of Cys. The selectivity of the carbonylacrylic reagents for Cys was corroborated using trypsin digestion followed by peptide mapping using mass spectrometry (Fig. 2e) or by chemical controls that involved Cys blocking using Ellman's reagent before reaction with **2c** (Supplementary Figs 18–20). Our data demonstrate that the new carbonylacrylic reagents are highly selective for Cys at pH 8.0, which contrasts with maleimides that show Lys cross-reactivity at slightly basic pH (maleimide bioconjugation is usually performed at slightly acidic pH∼6.0 to 7.0 to avoid potential Lys cross-reactivity).

### Stability and binding of albumin conjugates

We next evaluated the stability of the albumin conjugate with **2c** in human plasma, as well as in the presence of 10 mM GSH (Fig. 2f). Importantly, we observed that the thioether conjugate exhibited complete resilience to degradation remaining intact, as demonstrated by LC–MS analysis (Supplementary Fig. 21). Finally, by using surface plasma resonance, we confirmed that the conjugate of albumin retained its ability to bind to the neonatal Fc receptor (FcRn)^25^ (*K*_D_ (albumin)=16.13 μM; *K*_D_ (albumin-**2c**)=14.11 μM) (Supplementary Fig. 22). The data show the potential of our new method to generate stable and functional protein conjugates for *in vivo* applications.

### Synthesis of functionalized carbonylacrylic reagents

To achieve a general and effective bioconjugation method, it is important that different functional groups can be readily attached to the carbonylacrylic scaffold. Many relevant conjugation reagents such as fluorescent probes, polyethylene glycol (PEG) derivatives or drugs are commercially available bearing a free amine handle, thus allowing for easy access to functionalized carbonylacrylic reagents through amidation of the corresponding carboxylic acid intermediate (see Supplementary Methods for synthetic details). To further exemplify the utility of our method for bioconjugation we synthesized a fluorescent carbonylacrylic derivative **7** bearing nitrobenzofurazan (*λ*_ex_≈465 nm and *λ*_em_≈539 nm) and a PEGylated derivative **8** (Fig. 3a) and showed that albumin could be easily labelled using stoichiometric amounts of **7** or **8** within 1 to 2 h (Supplementary Figs 23–25).

### Precise protein fluorescent labelling

Many maleimide-based fluorescent labelling strategies, including those used to promote hydrolysis, can lead to fluorescence quenching of the synthetic probe attached to the protein^19^,^26^. To demonstrate the utility of our method to fluorescently labelled proteins, we decided to conjugate **7** to Annexin V and use the respective chemically defined Annexin V fluorescent conjugate to specifically label apoptotic cells by binding to the phosphatidylserine receptor on their cell membrane. Remarkably, the Cys selective installation of **7** into Annexin V (Fig. 3b and Supplementary Figs 26 and 27; for C2Am see Supplementary Figs 28 and 29) allowed for efficient labelling and visualization of apoptotic HEK293 cells (Fig. 3c). Moreover, the specificity of the observed labelling, which is generated by the increase of the phosphatidylserine receptor in the cell surface during apoptosis, was further confirmed through blocking of cells with non-labelled Annexin V before incubation with the fluorescently labelled Annexin V, which led to a significant loss of fluorescence (Fig. 3c,d). Together, these data not only demonstrated that probe fluorescence is retained after functionalization of the protein with the carbonylacrylic reagent, but also the utility of the method to create precisely modified fluorescently labelled proteins that maintain high levels of specific activity.

### Cys-selective antibody conjugation

**Figure 4:**
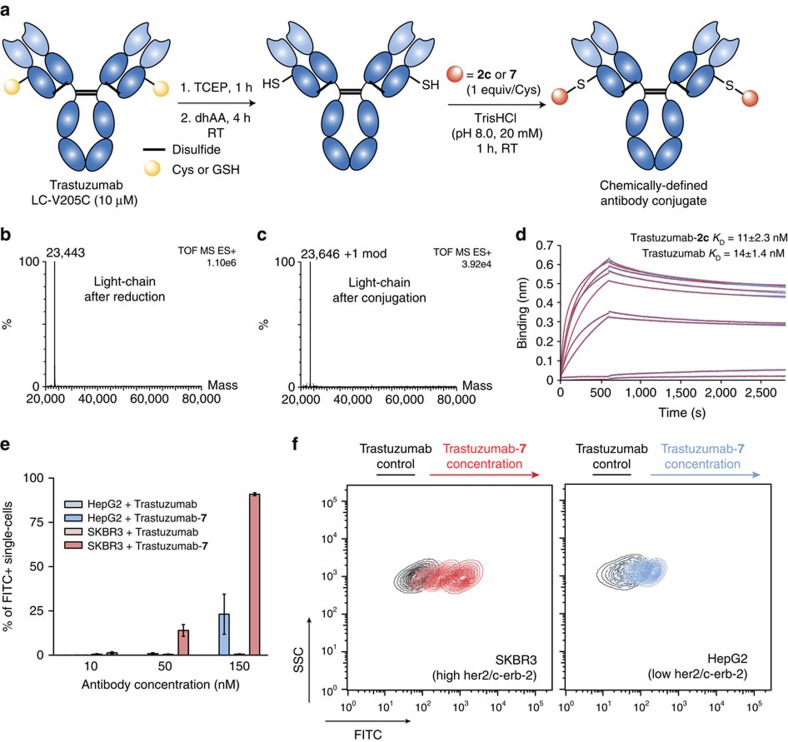
Construction of homogenous and functional antibody-conjugates. (**a**) Scheme for chemoselective and equimolar bioconjugation of Trastuzumab with **2c** and **7**. (**b**,**c**) Electrospray ionization–MS spectra of the light-chain of Trastuzumab (**b**) before and (**c**) after conjugation with **2c**. (**d**) BLI curves and fit for Trastuzumab-**2c** and HER2. *K*_D_ constants derived from BLI experiments for non-modified Trastuzumab and conjugated Trastuzumab-**2c**. (**e**,**f**) Analysis of specificity of the Trastuzumab-**7** towards HER2 by flow-cytometry. (**e**) Percentage of FITC-positive single cells, after treatment with fluorescent Trastuzumab-**7** or non-fluorescent Trastuzumab. (**f**) Superposition of contour plots of side-scatter detection versus FITC-equivalent fluorescence intensity, in SKBR3 cells (expressing high levels of her2/c-erb-2) and in HepG2 cells (expressing low levels of her2/c-erb-2). Controls were treated with non-conjugated Trastuzumab and samples were treated with increasing concentrations of Trastuzumab-**7** (10, 50 and 150 nM). RT, room temperature.

With a method in hand suitable for rapid and irreversible bioconjugation of Cys-tagged proteins, we next evaluated its application on antibody conjugation. Much to our delight, we found that the conjugation reaction proceeded with stoichiometric amounts of **2c** or **7** for 1 h at room temperature after tris(2-carboxyethyl)phosphine reduction (Supplementary Figs 30 and 31) and dehydroascorbic acid mediated disulfide re-oxidation of Trastuzumab LC-V205C (Fig. 4a). Mass spectrometry analysis showed a single modification in the light chain (Fig. 4b,c and Supplementary Fig. 32) while the heavy chain remained unmodified (Supplementary Fig. 33). This indicates a successful re-oxidation of the interchain disulfides with conjugation occurring only at the engineered free Cys in the light-chain, yielding a pure and chemically defined conjugate. Notably, our method improves the homogeneity, the quality of the antibody conjugate product, as conjugates of Trastuzumab LC–V205C prepared using maleimides result in a mixture of modified (∼80–90%) and non-modified species (∼10–20%; refs 13, 16, 27, 28). Importantly, the Trastuzumab-**2c** conjugate retained its binding activity to the HER2 antigen (*K*_D_=11±2.3 nM for the conjugate versus *K*_D_=14±1.4 nM for the unmodified antibody) as shown by bio-layer interferometry (BLI) (Fig. 4d and Supplementary Fig. 34). These results were corroborated by enzyme-linked immunosorbent assay (Supplementary Fig. 35). We also show by flow-cytometry analysis and using fluorescently labelled Trastuzumab-**7** (Supplementary Fig. 36) that the modified antibody retains its specificity towards SKBR3 cells, which express high levels of its target antigen (her2/c-erb-2), as opposed to HepG2 cells, which express low levels of its target (Fig. 4e,f and Supplementary Discussion). Moreover, fluorescently labelled Trastuzumab-**7** appears to be specific to her2/c-erb-2 receptors at an identical antibody concentration to that previously reported for Trastuzumab^29^. Also, at 150 nM, percentages of her2–positive cells in each cell line also are in accordance with previous reports using Trastuzumab and other commercially available antibodies targeting her2–receptors^29^. At higher concentrations, we observed unspecific binding of the antibody, while at concentrations lower than 10 nM, there was no observable staining (data not shown). Our flow-cytometry data clearly shows that the modified Trastzumab-**7** retains its specificity towards her2 receptors. Together, these results demonstrate the utility of our new bioconjugation method for the generation of homogenous and functional antibody-conjugates using equimolar (1 equivalent per Cys) quantities of the carbonylacrylic reagents here reported.

In summary, a one-step and irreversible Cys selective bioconjugation is reported using simple carbonylacrylic reagents. The reaction proceeds with a high degree of Cys selectivity using equimolar amounts of synthetically accessible reagents under aqueous biologically friendly conditions. The conjugates formed are fully stable when exposed to GSH and in plasma, and retain their function, as evidenced by the selective imaging and detection of apoptotic and HER2–positive cells, respectively. The superior reactivity and Cys selectivity of the carbonylacrylic reagents enable access to fully homogenous conjugates including the antibody Trastuzumab as opposed to the antibody conjugate prepared using maleimides. Our method is likely to be of a general benefit for other sensitive Cys-tagged protein/antibody systems, many of which are already available through large-scale production. As such, we are confident that the direct chemoselective and irreversible Cys bioconjugation technology disclosed herein will find a significant use for the preparation of imaging and therapeutic conjugates for *in vivo* purposes.

## Methods

### Synthetic details

For detailed procedures and characterization, see the Supplementary Methods. For ^1^H and ^13^C NMR spectra of the compounds in this article, see Supplementary Figs 37–46.

### General procedure for antibody conjugation

Trastuzumab LC-V205C (15 μl, 400 nmol) disulfide bonds were reduced by mild reduction in TrisHCl (pH 8.0, 20 mM) (23 μl) at 25 °C by the addition of tenfold molar excess reducing agent tris(2-carboxyethyl)phosphine (1 μl, 4,000 μM) for 1.5 h, followed by filtration using a Zeba Spin Desalting Column previously equilibrated with TrisHCl (pH 8.0, 20 mM). The sample was eluted *via* centrifugation (2 min, 1,500*g*). To re-form the interchain disulfide bonds, the reduced Trastuzumab was incubated for 4 h at 25 °C with dehydroascorbic acid at a tenfold molar excess. After, a 5 μl aliquot of reaction mixture was analysed by LC–MS (a 5 μl aliquot was diluted with 5 μl TrisHCl buffer (pH 8.0, 20 mM)). Finally, to an eppendorf with 20 μl of re-oxidized Trastuzumab LC-V205C was added a 0.48 μl aliquot of a stock solution of **2c** or **7** (1 equivalent/Cys) in dimethylformamide (1% of total volume) and the reaction was mixed for 1 h at room temperature. A 10 μl aliquot was analysed by LC–MS and complete conversion to the expected product was observed, that is, modification of the Cys on the light chain (Supplementary Fig. 32) and no modification in the heavy-chain (Supplementary Fig. 33).

### LC–MS analysis of protein conjugation

LC–MS was performed on a Xevo G2-S TOF mass spectrometer coupled to an Acquity high-performance liquid chromatography (UPLC) system using an Acquity UPLC BEH300 C4 column (1.7 μm, 2.1 × 50 mm). Solvents A, a water with 0.1% formic acid and B, 71% acetonitrile, 29% water and 0.075% formic acid were used as the mobile phase at a flow rate of 0.2 ml min^–1^. The gradient was programmed as follows: 72% A to 100% B after 25 min then 100% B for 2 min and after that 72% A for 18 min. The electrospray source was operated with a capillary voltage of 2.0 kV and a cone voltage of 40 V. Nitrogen was used as the desolvation gas at a total flow of 850 l h^–1^. Total mass spectra were reconstructed from the ion series using the MaxEnt algorithm preinstalled on MassLynx software (v. 4.1 from Waters) according to the manufacturer's instructions. To obtain the ion series described, the major peak(s) of the chromatogram were selected for integration and further analysis. See Supplementary Fig. 13.

### Tryptic digestion and MS/MS analysis

The protein solution was reduced, alkylated and digested with trypsin overnight. Post-digestion, the solution was pipetted into a vial and placed in the autosampler of the LC pump. All LC–MS/MS experiments were performed using a Dionex Ultimate 3,000 RSLC nanoUPLC (Thermo Fisher Scientific Inc, Waltham, MA, USA) system and a QExactive Orbitrap mass spectrometer (Thermo Fisher Scientific Inc, Waltham, MA, USA). Separation of peptides was performed by reverse-phase chromatography at a flow rate of 300 nl min^−1^ and a Thermo Scientific reverse-phase nano Easy-spray column (Thermo Scientific PepMap C18, 2 μm particle size, 100A pore size, 75 μm × 50 cm length). Peptides were loaded onto a pre-column (Thermo Scientific PepMap 100 C18, 5 μm particle size, 100A pore size, 300 nm × 5 mm length) from the Ultimate 3,000 autosampler with 0.1% formic acid for 3 min at a flow rate of 10 μl min^−1^. After this period, the column valve was switched to allow elution of peptides from the pre-column onto the analytical column. Solvent A was water +0.1% formic acid and solvent B was 80% acetonitrile, 20% water +0.1% formic acid. The linear gradient employed was 2–40% B in 30 min. The LC eluent was sprayed into the mass spectrometer by means of an Easy-spray source (Thermo Fisher Scientific Inc.). All *m/z* values of eluting ions were measured in an Orbitrap mass analyser, set at a resolution of 70,000. Data dependent scans (Top 20) were employed to automatically isolate and generate fragment ions by higher energy collisional dissociation in the quadrupole mass analyser and measurement of the resulting fragment ions was performed in the Orbitrap analyser, set at a resolution of 17,500. Peptide ions with charge states of 2+ and above were selected for fragmentation. Post-run, the data was processed using Protein Discoverer (version 1.4., ThermoFisher). Briefly, all MS/MS data were converted to mgf files and these were submitted to the Mascot search algorithm (Matrix Science, London UK) and searched against a custom database containing the C2A domain of synaptotagmin-I sequence and applying a fixed modification of carbamidomethyl (C) and variable modifications of oxidation (M), deamidation (NQ) and a custom modification of C2Am-**2c** (C), using a peptide tolerance of 5 p.p.m. (MS) and 0.1Da (MS/MS). Peptide identifications were accepted if they could be established at >95.0% probability.

### Protein gels

The incubation solution (5.0 μl) was transferred to tube, and NuPAGE LDS Sample Buffer (4 × , 2.5 μl), NuPAGE Reducing Agent (10 × , 1 μl), and H_2_O (1.5 μl) were added to the tube. The solution was heated at 70 °C for 10 min. The heated solution was loaded to NuPAGE Bis-Tris mini gel (10 × 10 cm) with 4–12% gradient polyacrylamide concentration, and then the conjugation reaction was analysed by electrophoresis (200 V). The buffering system employed was 1 × SDS Running Buffer (NuPAGE MES SDS Running Buffer, 20 × , pH 7.3, 50 to 950 ml deionized water). For reduced samples, 500 μl of NuPAGE Antioxidant was added to each 200 ml 1 × SDS running buffer. After 35 min, the intensities of fluorescence were analysed. Then, the gel was stained with 0.5% of Ruby. The gel was mixed overnight at room temperature and read the day after. After washing the gel, coomassie (0.5%) was added and the gel was read 2 h after mixing at room temperature. See Supplementary Figs 24, 27, 29 and 36.

### Microscopy studies

Human embryonic kidney HEK293 cells were maintained at 37 °C in a humidified atmosphere of 5% CO_2_ in DMEM high glucose (Gibco) supplemented with 10% heat inactivated fetal bovine serum (HI-FBS, Gibco) and 1X antibiotic-antimycotic (Gibco). When cells have reached the appropriate density (70–80% confluent), the medium was aspirated and cells harvested with 0.25% Trypsin-EDTA. Then, 200 μl of the cell suspension (∼50,000 cells) was applied on top of 12 mm glass coverslips pre-coated with poly D-lysine (Corning BioCoat) placed inside a 24-well plate. After 1 h of incubation to allow cells to adhere, 200 μl of additional media was added to flood the wells. Cells were then grown for more 7 h at 37 °C before apoptosis was induced by treatment for 12 h with 2 μM of actinomycin D in fresh growth media. Untreated cells at the same density were included as a control. After apoptosis induction the media was removed, cells were washed with D-PBS (Gibco) and incubated at 37 °C for 20 min with **7** at a concentration of 0.2 μM in 10 mM HEPES pH 7.4 supplemented with 140 mM NaCl and 2.5 mM CaCl_2_ (annexin binding buffer from Molecular Probes). At the same time blocking studies were performed by pre-incubating apoptotic cells with a 25 × excess of non-fluorescent Annexin V wild type (5 μM, 37 °C for 20 min) before incubation with the fluorescent probe **7** for additional 20 min at 37 °C. For fluorescent DNA nuclei staining, Hoechst 33,342 (0.8 μg ml^−1^, Sigma Aldrich, 15 min at 37 °C) was used. After labelling, the cells were washed with PBS two times. Finally, cells were fixed with PBS containing 4% (w/v) formaldehyde for 15 min at room temperature, further washed two times with PBS, and mounted on slides with Ibidi mounting medium. Fluorescence microscopy was performed using an inverted epifluorescent microscope (Olympus IX-71) connected to a F-view digital camera (Soft Imaging System). Images were acquired in the fluorescein isothiocyanate (FITC) and Hoechst channels and analysed using the software Cell-F. Identical image acquisition settings were used for the control, experimental and blocking data sets.

### Determination of antibody conjugates binding affinity

#### Biotinylation of antibodies

Non-modified Trastuzumab and Trastuzumab-**2c** were conjugated to a biotin linker using Biotin-(PEG)_4_-N-hydroxysuccinimide(NHS) (Thermofisher Scientific) in order to carry out BLI experiments using Streptavidin (SA) Biosensors. A solution of EZ-Link NHS-(PEG)_4_-Biotin (20 μl, 200 μM in PBS) was added to the corresponding protein (20 μl, 20 μM in PBS) and was left at room temperature for 30 min. The crude reaction mixture was buffer exchanged with PBS for three times to remove the excess of NHS-(PEG)_4_-Biotin, obtaining a biotin-to-antibody ratio around 1.6.

#### Bio-layer interferometry

Binding assays were performed on an Octet Red Instrument (fortéBIO). Ligand immobilization, binding reactions, regeneration and washes were conducted in wells of black polypropylene 96-well microplates. Trastuzumab and Trastuzumab-**2c** (20 nM) were immobilized on Streptavidin (SA) Biosensors in PBS with 0.1% BSA and 0.02% tween at 30 °C. Binding analysis were carried out at 25 °C, 1,000 r.p.m. in PBS pH 7.4 with 0.1% BSA and 0.02% tween, with a 600 s of association followed by a 2,200 s of dissociation, using different concentrations of the recombinant HER2 receptor to obtain the association curve. Glycine pH 2.0 was used as a regeneration buffer. Data were analysed using Data Analysis (fortéBIO), with Savitzky-Golay filtering. Binding was fitted to a 2:1 Heterogeneous ligand model, steady state analysis were performed to obtain the binding kinetics constants (*K*_D_). See Supplementary Fig. 34.

### Determination of antibody conjugates specificity

#### Cell culture conditions

SKBR3 (ATCC HTB-30) and HepG2 (ATCC HB-8065) cells were routinely grown in a humidified incubator at 37 °C under 5% CO_2_ and split before reaching confluence using TrypLE Express. Both cells lines were grown in DMEM medium supplemented with 10% heat-inactivated FBS, 2 mM GlutaMAX, 10 mM HEPES, 1% non-essential amino acids (NEAA), 1 mM sodium pyruvate, 100 units ml^−1^ penicillin and 100 μg ml^−1^ streptomycin. All reagents were bought from Gibco, Life Technologies (USA), unless otherwise stated.

#### Flow cytometry

SKBR3 and HepG2 cells were seeded in 24 well-plates at a concentration of 50,000 cells per well and 30,000 cells per well, respectively, and cultured for 48 h before being used for experimentation. Cells were detached from the plates and collected into flow cytometry round bottom polystyrene tubes. Culture medium was removed and cells were re-suspended in complete medium supplemented with either Trastuzumab or Trastuzumab-**7** (at 5, 10, 50, 150, 300 and 500 nM) and incubated at room temperature, in the dark. After 1 h, cell suspensions were centrifuged at 400 g for 4 min, the medium was removed and cells were re-suspended in 300 μl of a PBS solution supplemented with 10% FBS. Cells were kept in the dark until analysis. Acquisition was performed on a LSRFortessa^TM^ flow cytometer (BD Biosciences, USA) set up with a 488 nm laser and a combination of a 505 nm long-pass and a 530/30 nm band-pass filter (combination used for FITC detection). Due to expected overlap between the emission spectrum of Trastuzumab-**7** with the one of R-phycoerythrin (PE) channels, data were also acquired with the combination of filters normally used for its detection (a 550 nm long-pass and a 575/26 nm band-pass filter), and compensation was performed between these channels. Only results for single-cell events are shown, and data are expressed as the average of three independent experiments±s.e.m.

### Data availability

Additional information and the data supporting this research are available within the article and the Supplementary Information file and from the corresponding author upon reasonable request.

## Additional information

**How to cite this article:** Bernardim, B. *et al*. Stoichiometric and irreversible cysteine-selective protein modification using carbonylacrylic reagents. *Nat. Commun.* **7,** 13128 doi: 10.1038/ncomms13128 (2016).

## Supplementary Material

Supplementary Information Supplementary Figures 1-46, Supplementary Table 1, Supplementary Methods, Supplementary Discussion and Supplementary References.

Supplementary Dataset 1 Cartesian coordinates of the lowest energy calculated structures.

## Figures and Tables

**Table 1:** Cys-bioconjugation on proteins.

**Cys-protein (10** μ**M)**	**2**	**Equivalent**	**Temperature (°C)**	**Time (h)**	**Conversion (%)**
Annexin V	**2a**	25	25	27	30†
Annexin V	**2b**	25	25	27	30†
Annexin V	**2c**	25	25	27	60
Annexin V	**2d**	25	25	27	0
Annexin V	**2c**	50	37	1	>95
C2Am	**2c**	25	25	1	>95
Albumin	**2c**	1	25	2	>95

Identification of an efficient carbonylacrylic reagent and reaction optimization for site-selective Cys modification on proteins.*

^*^General bioconjugation conditions: protein (10 μM) in TrisHCl (pH 8.0, 20 mM), carbonylacrylic derivative **2a–d** (1–50 equiv.) dissolved in dimethylformamide (1–10%) were reacted in an Eppendorf tube for the indicated time.

^†^Hydrolysis of the ester was observed by LC–MS. See Supplementary Methods and Supplementary Figs 8–12 for full details. The bold numbers are the compound numbers.
