# Supplementary material for: Stoichiometric and irreversible cysteine-selective protein modification using carbonylacrylic reagents
Source: Nat Commun. 2016 Oct 26;7:13128. doi: 10.1038/ncomms13128 (PMC5095172; doi:10.1038/ncomms13128)
Supplement: Supplementary Information — Supplementary Figures 1-46, Supplementary Table 1, Supplementary Methods, Supplementary Discussion and Supplementary References. [file ncomms13128-s1.pdf]

Supplementary Figures

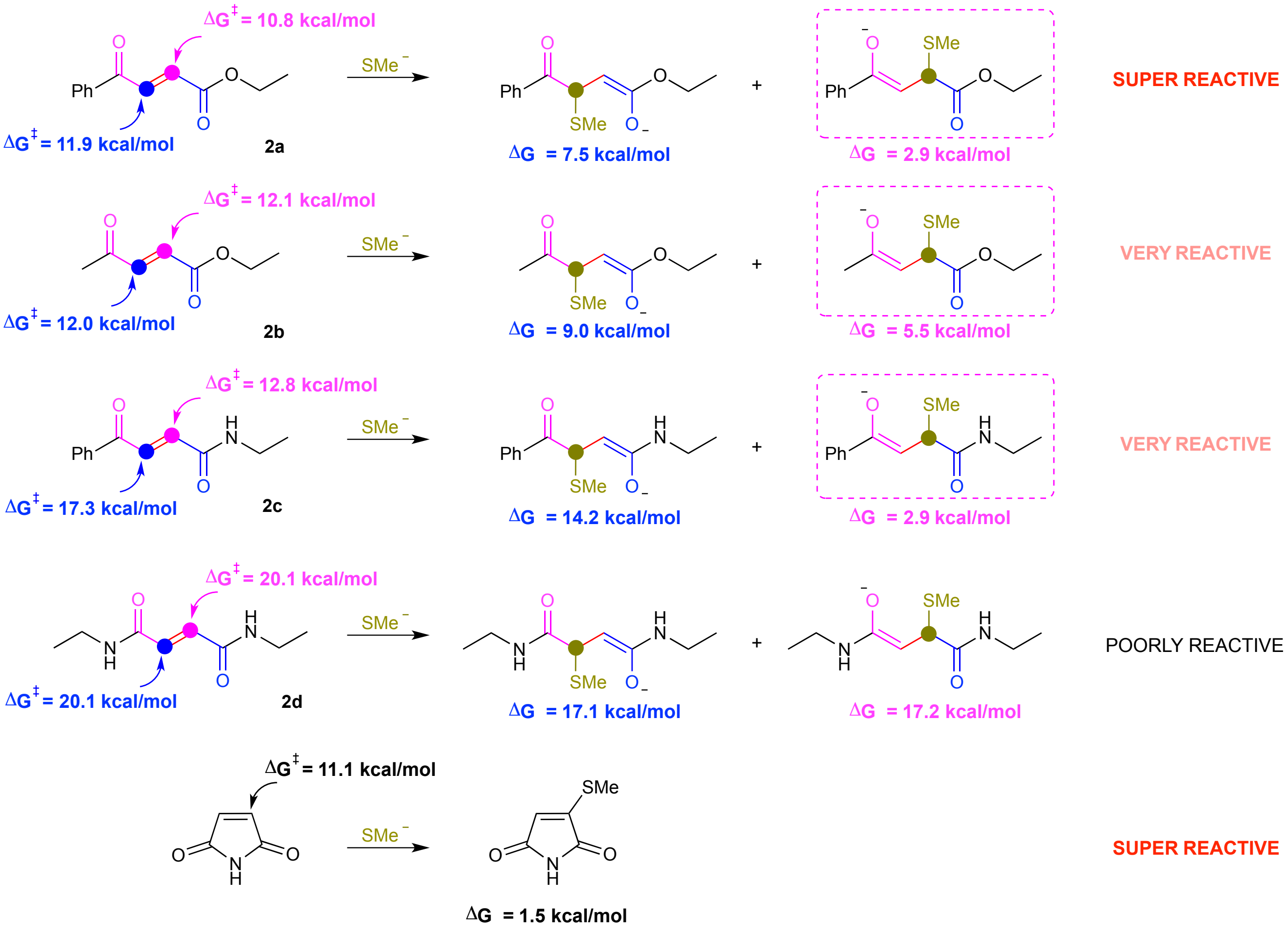

Supplementary Fig. 1 Activation (ΔG‡) and reaction (ΔG) free energies. These were calculated with PCM_{water}/M06-2X/6-31+G(d,p) for the Michael addition of methanethiolate anion to carbonylacrylic reagents.

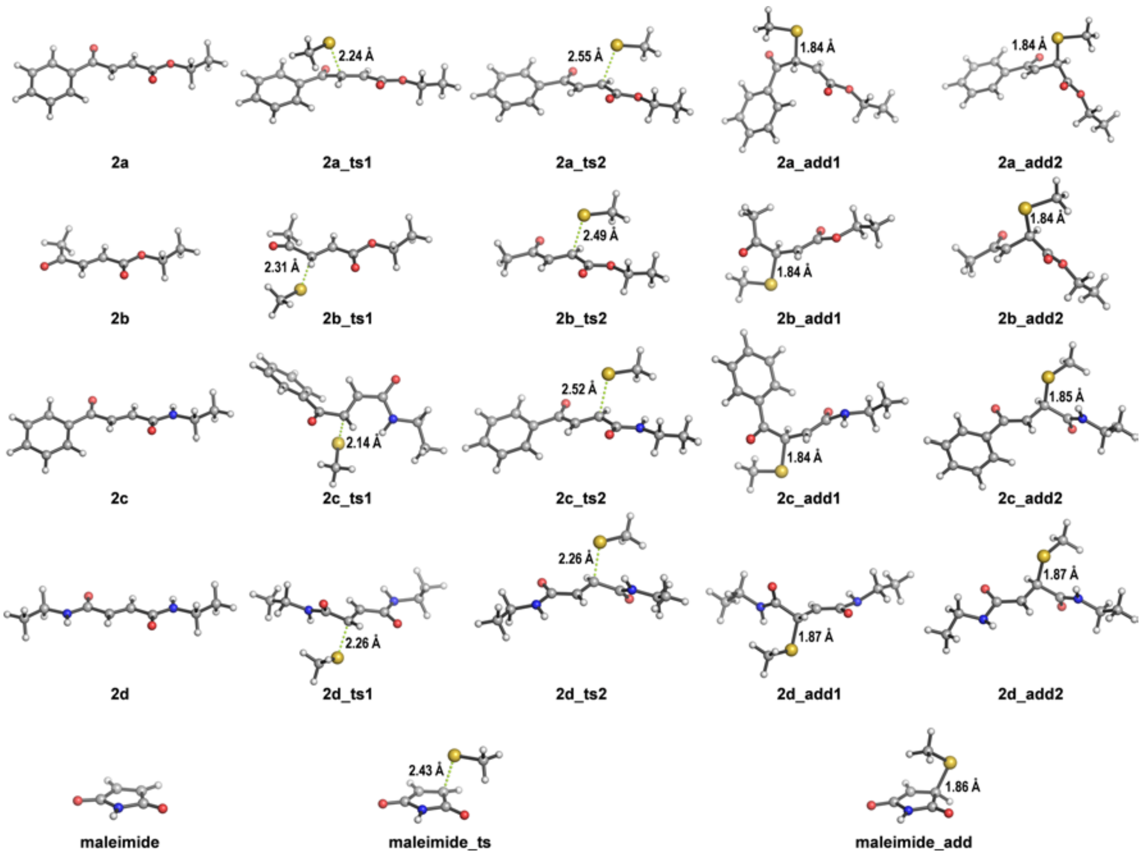

Supplementary Fig. 2 Guide to compound numbering of calculated structures. Note: Only the lowest energy conformers are shown.

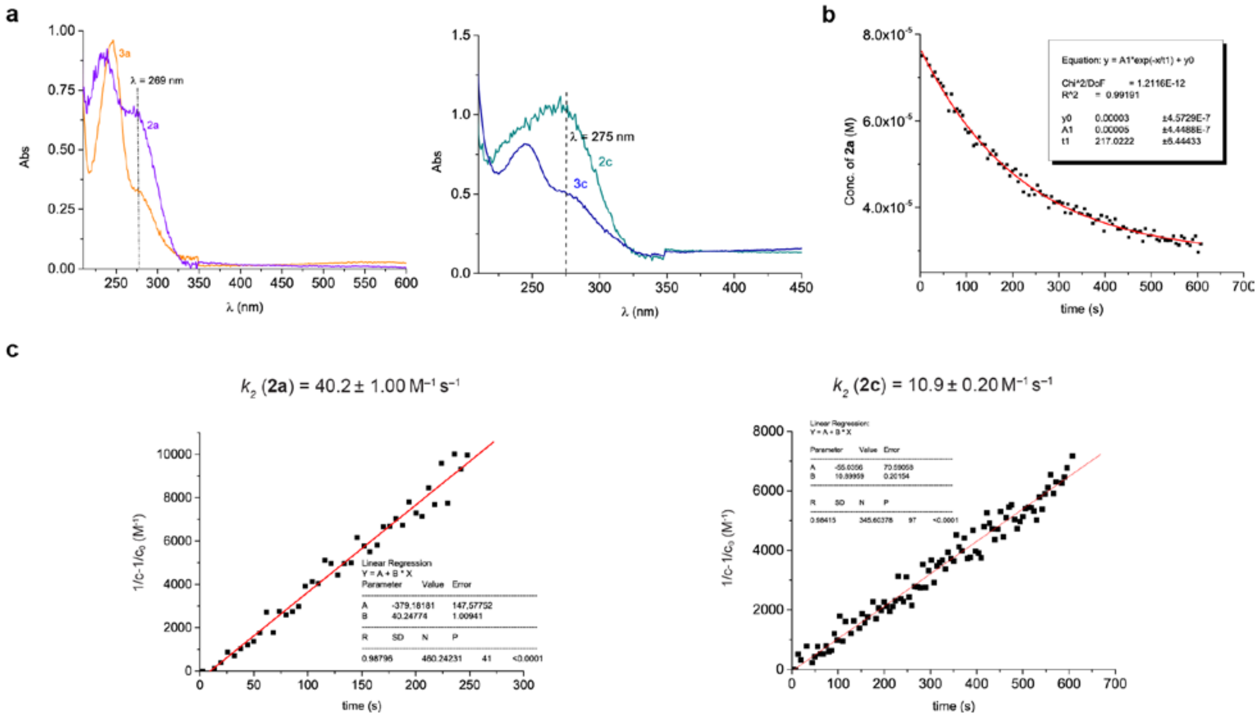

Supplementary Fig. 3 Calculation of reaction kinetics. (**a**) UV spectra of compounds **2a** and **2c**, together with the UV spectra of the corresponding adducts **3a** and **3c** obtained after reaction with **1**. (**b**) Variation of concentration of compound **2a** *versus* time determined by measuring the absorbance at 269 nm. (**c**) Experimental determination of the second order rate constant for the addition of compound **2a** (left panel) and **2c** (right panel) to cysteine derivative **1**.

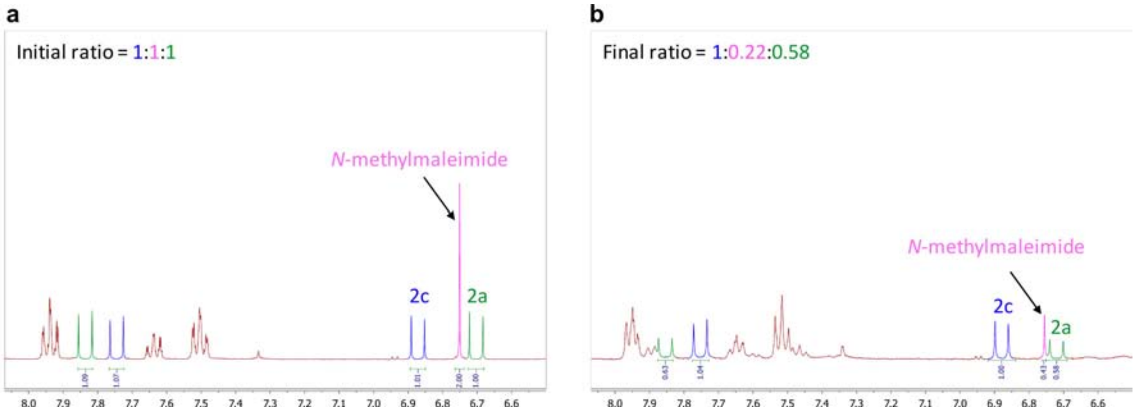

Supplementary Fig. 4. Competition experiment of the reaction between 2a, 2c and *N*-methylmaleimide and 1a. (**a**) ^{1}H NMR (400 Hz) of a mixture of compounds **2a**,**2c** and *N*-methyl maleimide (ratio 1:1:1), recorded at 20 ºC in 30% CD_3CN in deuterated sodium phosphate buffer (pH 8.0, 50 mM). (**b**) Product distribution obtained by ^{1}H NMR (400 Hz), recorded under same experimental conditions, after adding 1 equiv. of compound **1** to the mixture of Michael acceptors.

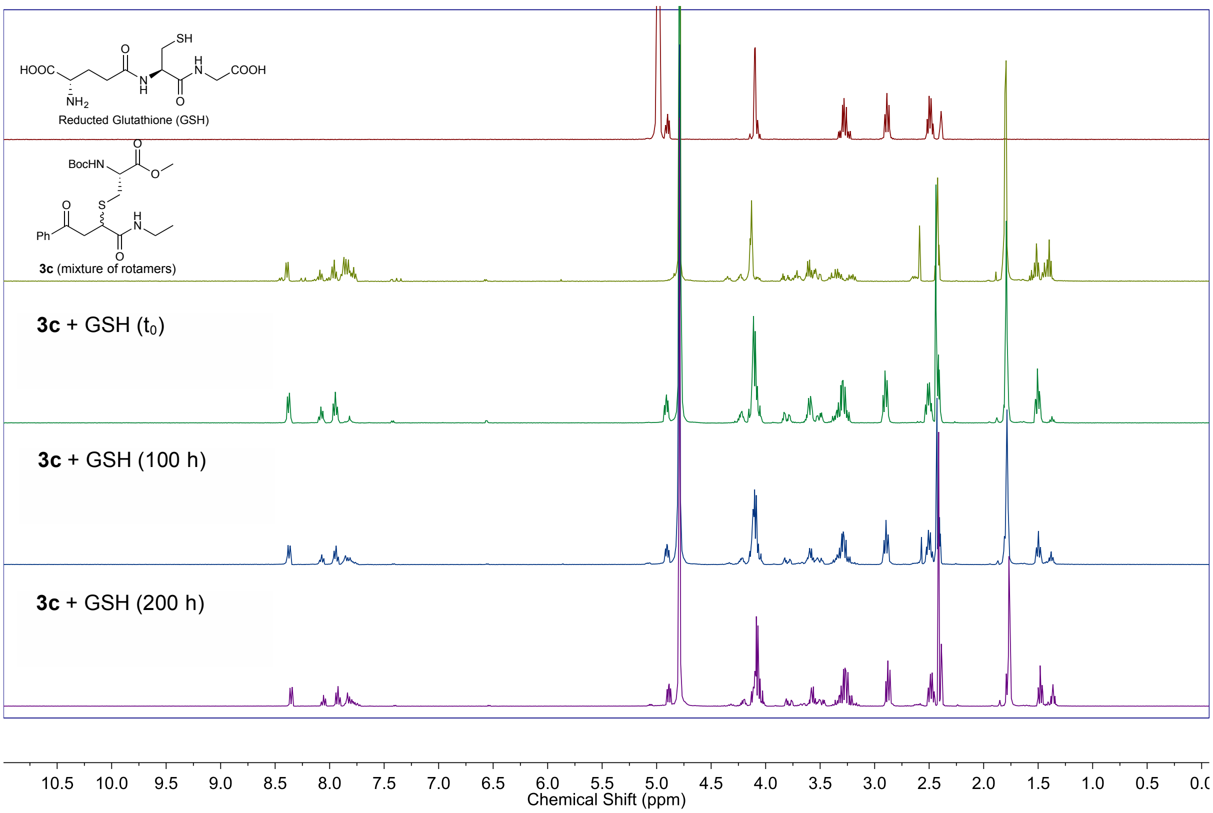

Supplementary Fig. 5 Stability of thiol Michael-addition product 3c.

Glutathione exchange studies by ^{1}H NMR.

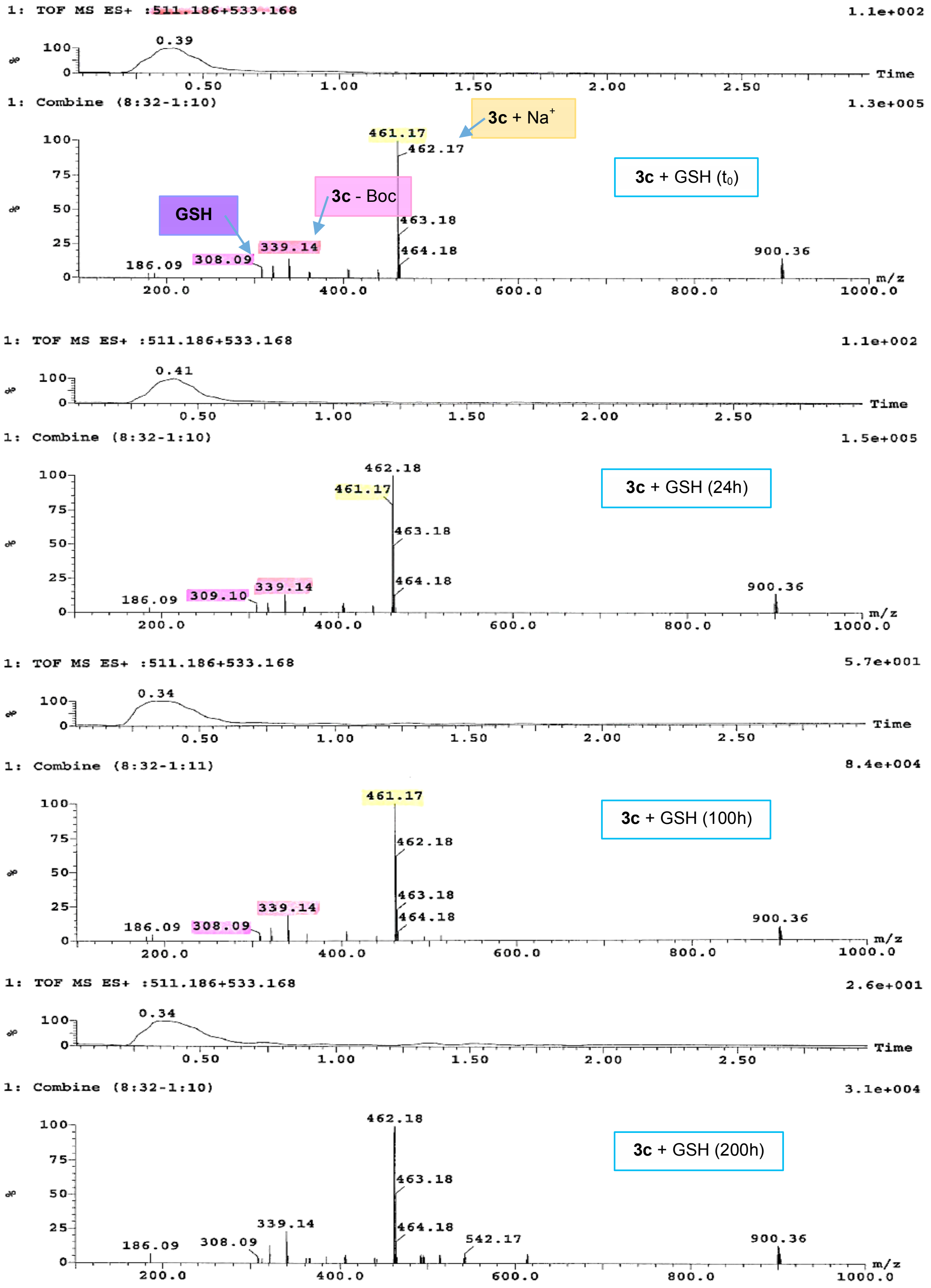

Supplementary Fig. 6 Stability of thiol Michael-addition product 3c.

Glutathione exchange studies by LRMS.

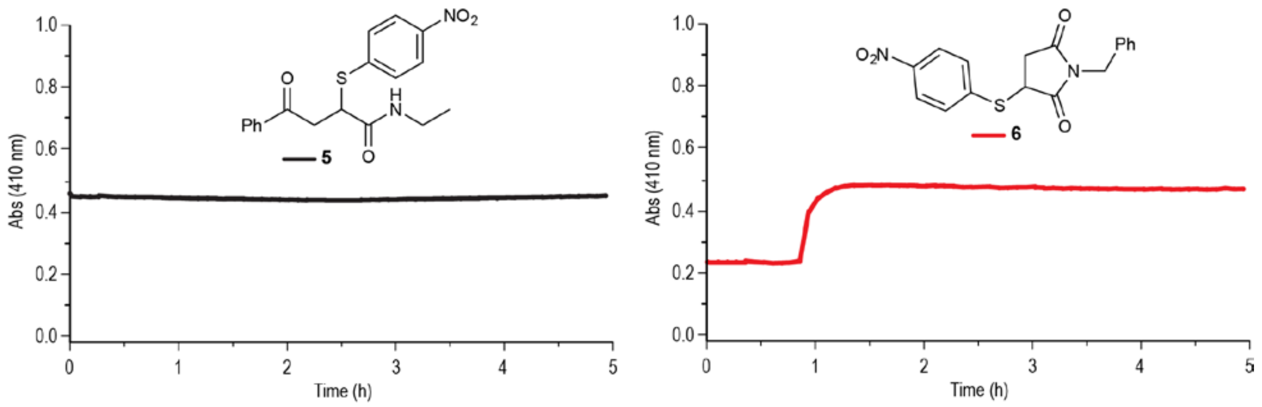

Supplementary Fig. 7 Comparison of kinetic stability of 5 *vs* maleimide derivative 6.

Kinetic assays for reverse Michael reactions on chromogenic thiol adducts **5** and **6**.

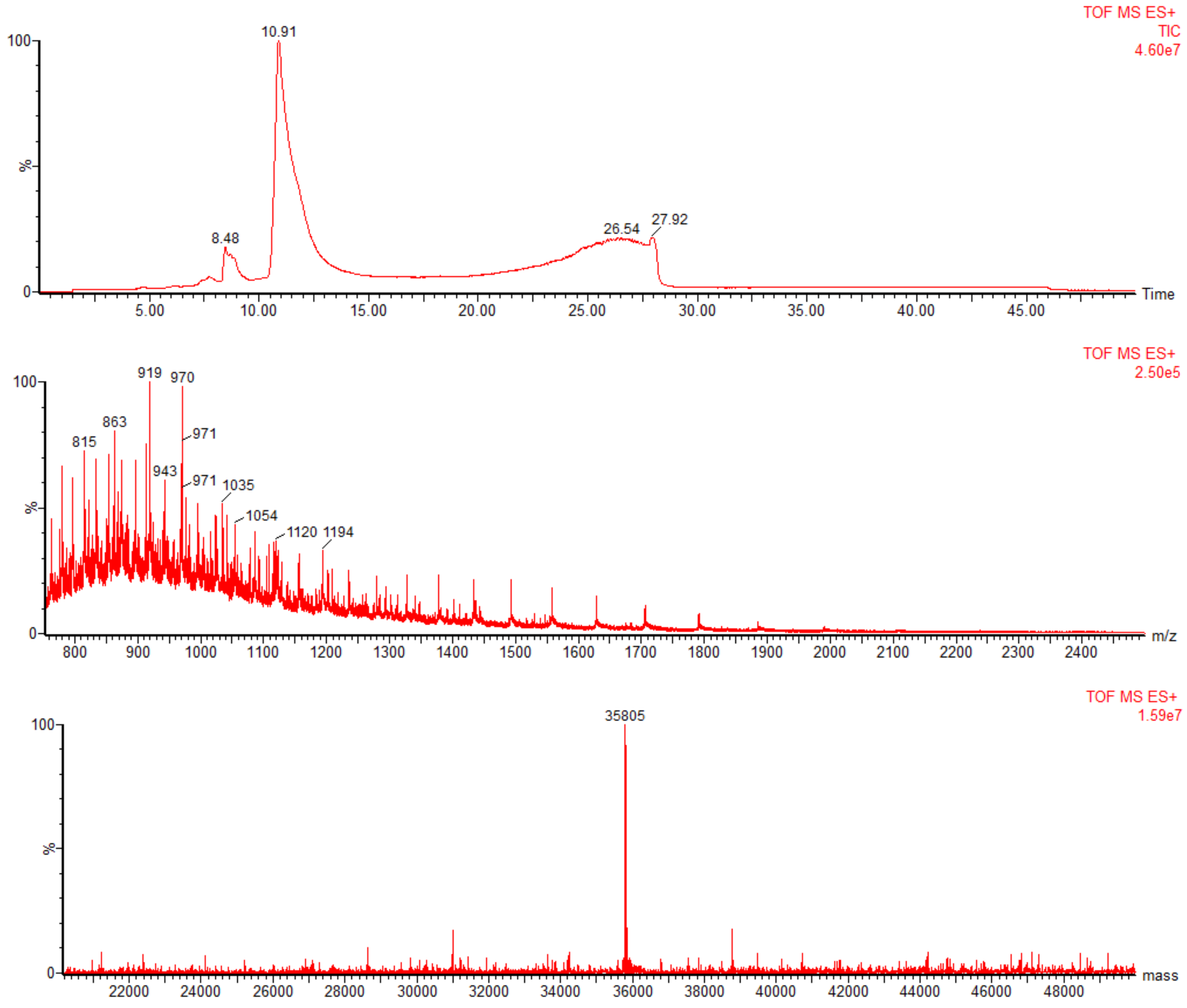

Supplementary Fig. 8 LC–MS analysis of Annexin V-Cys315. Total ion chromatogram, combined ion series and deconvoluted mass spectrum reconstructed from the ion series using the MaxEnt algorithm.

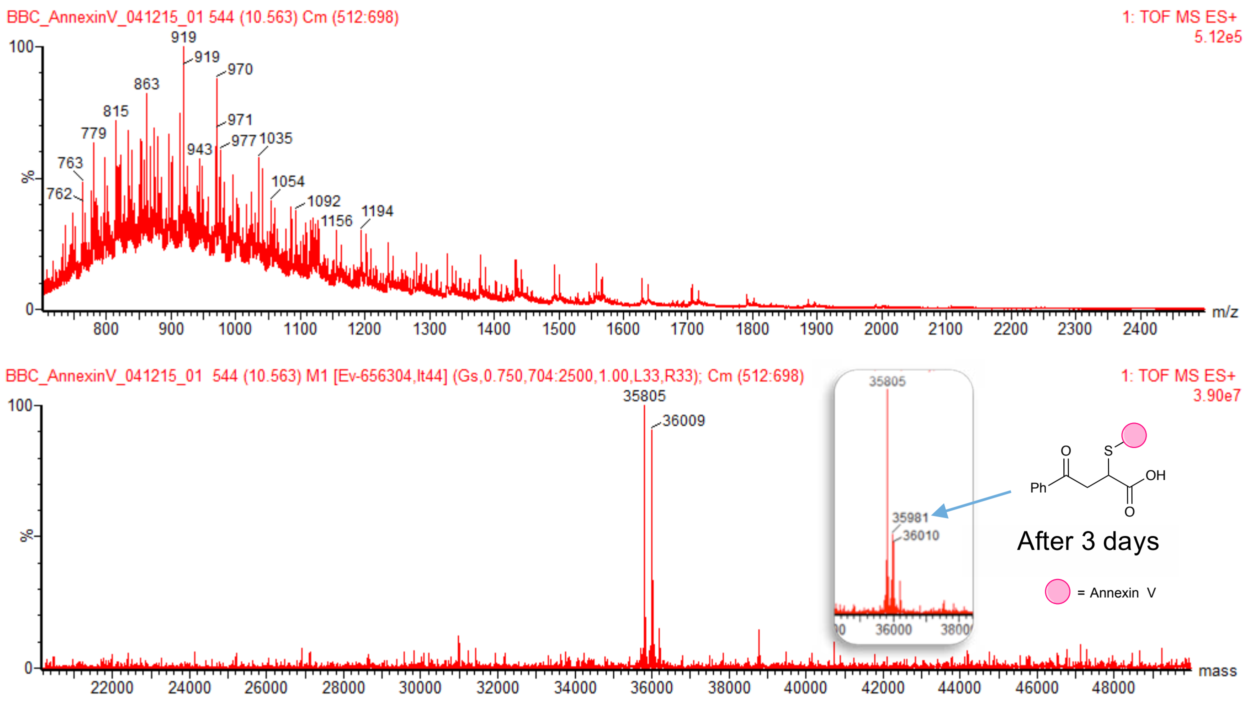

Supplementary Fig. 9 LC–MS analysis of the reaction of Annexin V with ethyl (*E*)-4-oxo-4-phenylbut-2-enoate 2a after 27 h and 3 days at RT. Combined ion series and deconvoluted mass spectrum reconstructed from the ion series using the MaxEnt algorithm.

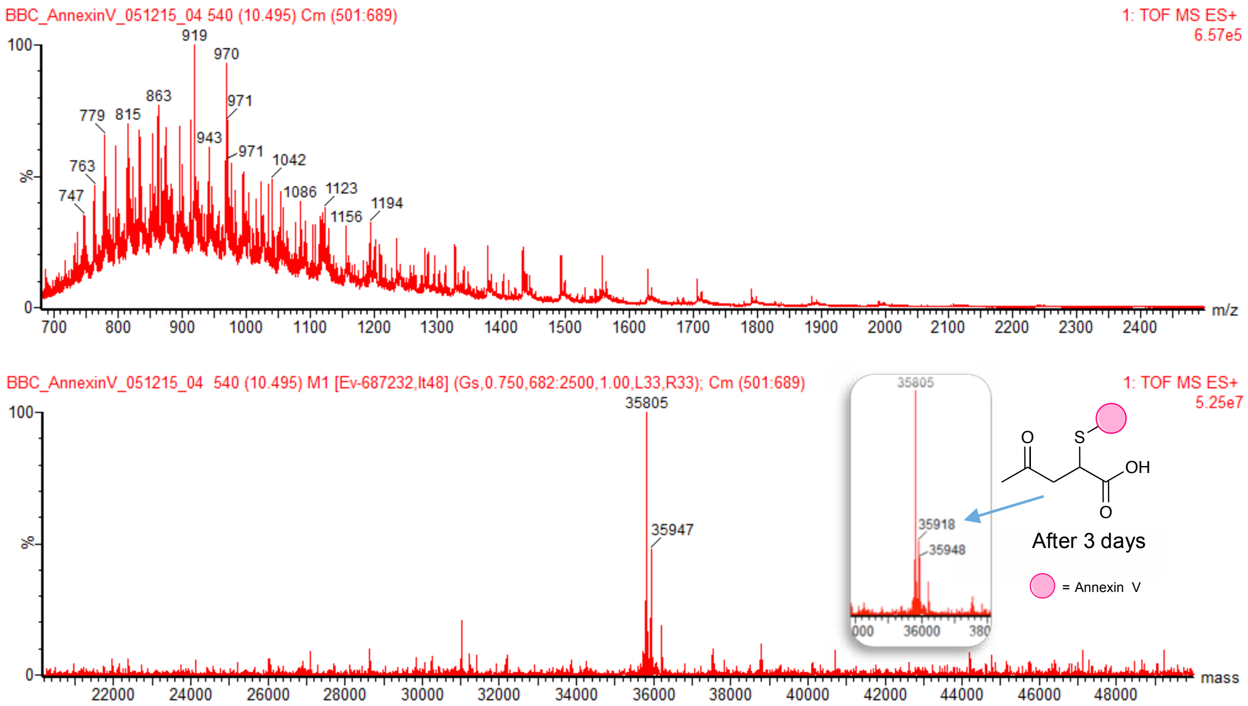

Supplementary Fig. 10 LC–MS analysis of the reaction of Annexin V with ethyl (*E*)-4-oxopent-2-enoate 2b after 27 h and 3 days at RT. Combined ion series and deconvoluted mass spectrum reconstructed from the ion series using the MaxEnt algorithm.

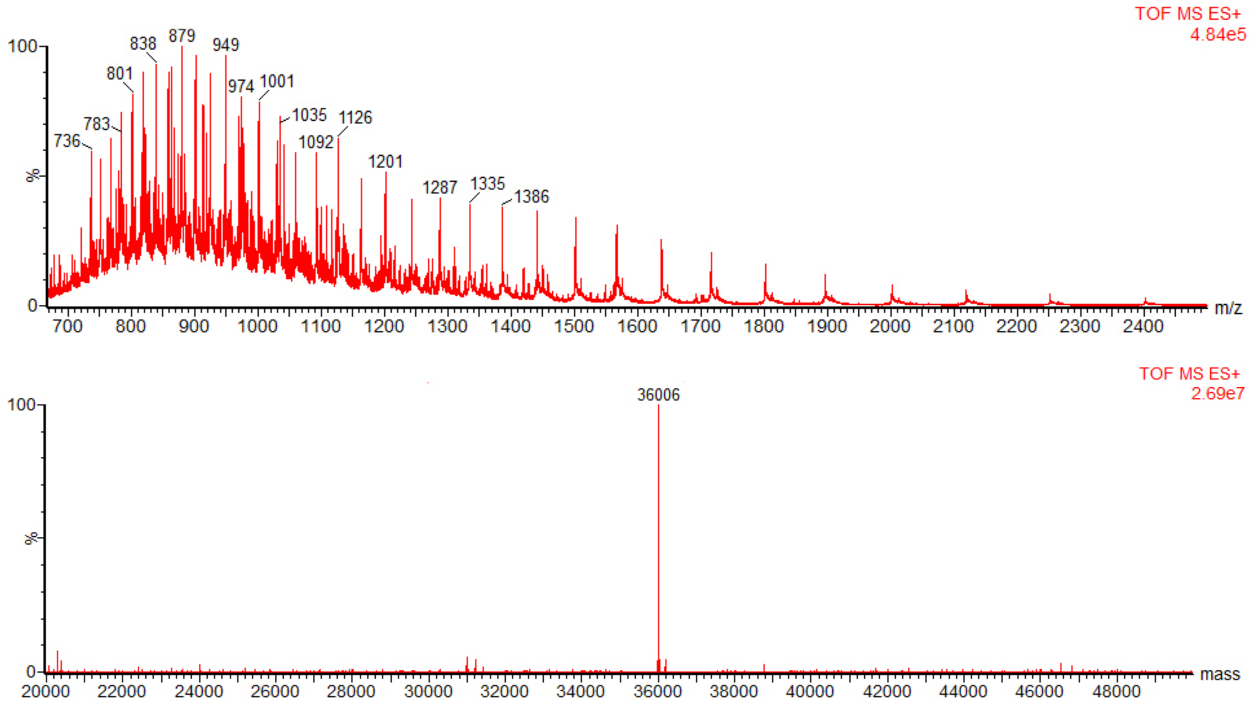

Supplementary Fig. 11 LC–MS analysis of the reaction of Annexin V with (*E*)-*N*-ethyl-4-oxo-4-phenylbut-2-enamide 2c after 1 h at 37 ºC. Combined ion series and deconvoluted mass spectrum reconstructed from the ion series using the MaxEnt algorithm.

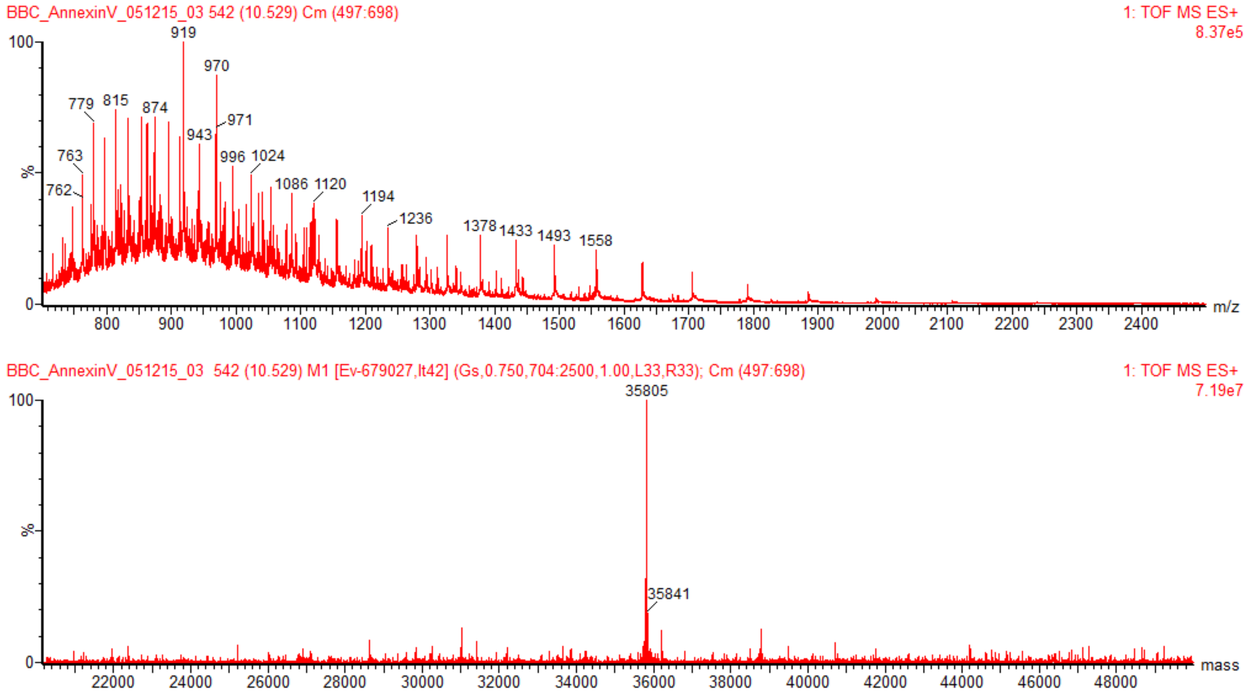

Supplementary Fig. 12 LC–MS analysis of the reaction of Annexin V with *N*$_1$,*N*$_4$-diethylfumaramide 2d after 27 h at RT. Combined ion series and deconvoluted mass spectrum reconstructed from the ion series using the MaxEnt algorithm.

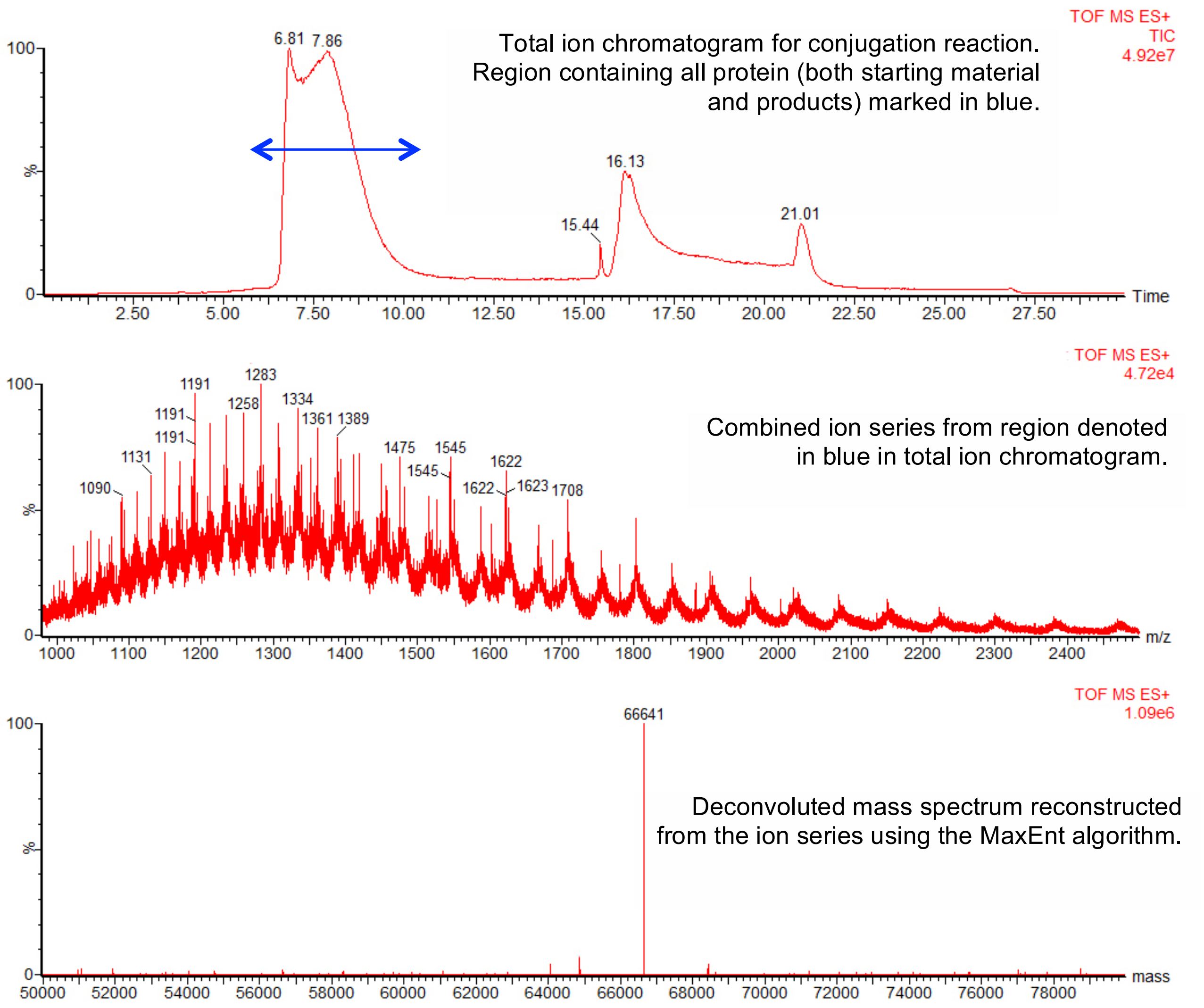

Supplementary Figure 13. A typical analysis of a conjugation reaction by LC–MS is described for the albumin protein. The total ion chromatogram, combined ion series and deconvoluted spectra are shown for the product of the reaction of albumin with 1 equiv. of (*E*)-*N*-ethyl-4-oxo-4-phenylbut-2-enamide **2c**. Identical analyses were carried out for all the conjugation reactions performed in this work. A LC-MS spectrum were collected for all proteins before the reaction with the carbonylacrylic reagent.

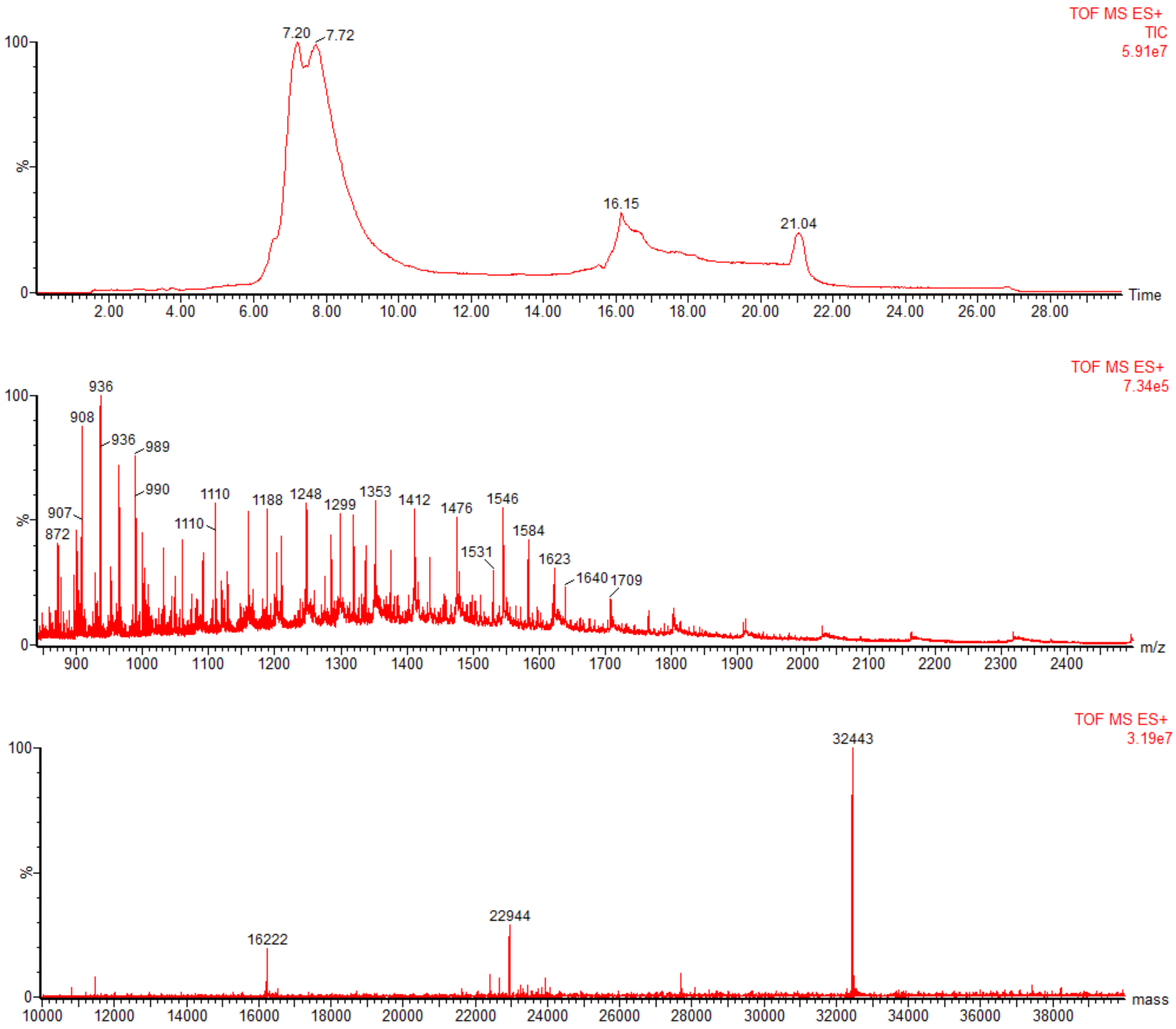

Supplementary Fig. 14 LC–MS analysis of C2Am-Cys95. Total ion chromatogram, combined ion series and deconvoluted mass spectrum reconstructed from the ion series using the MaxEnt algorithm.

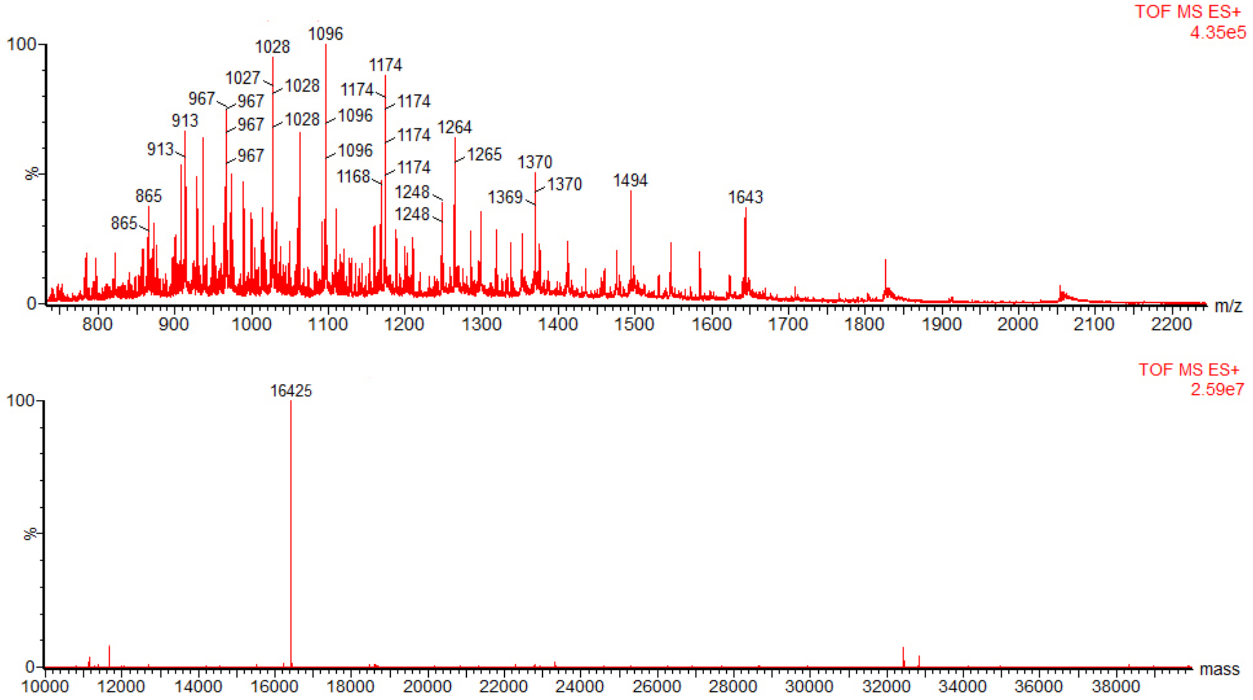

Supplementary Fig. 15 LC–MS analysis of the reaction of C2Am with 2c after 1 h at RT. Combined ion series and deconvoluted mass spectrum reconstructed from the ion series using the MaxEnt algorithm.

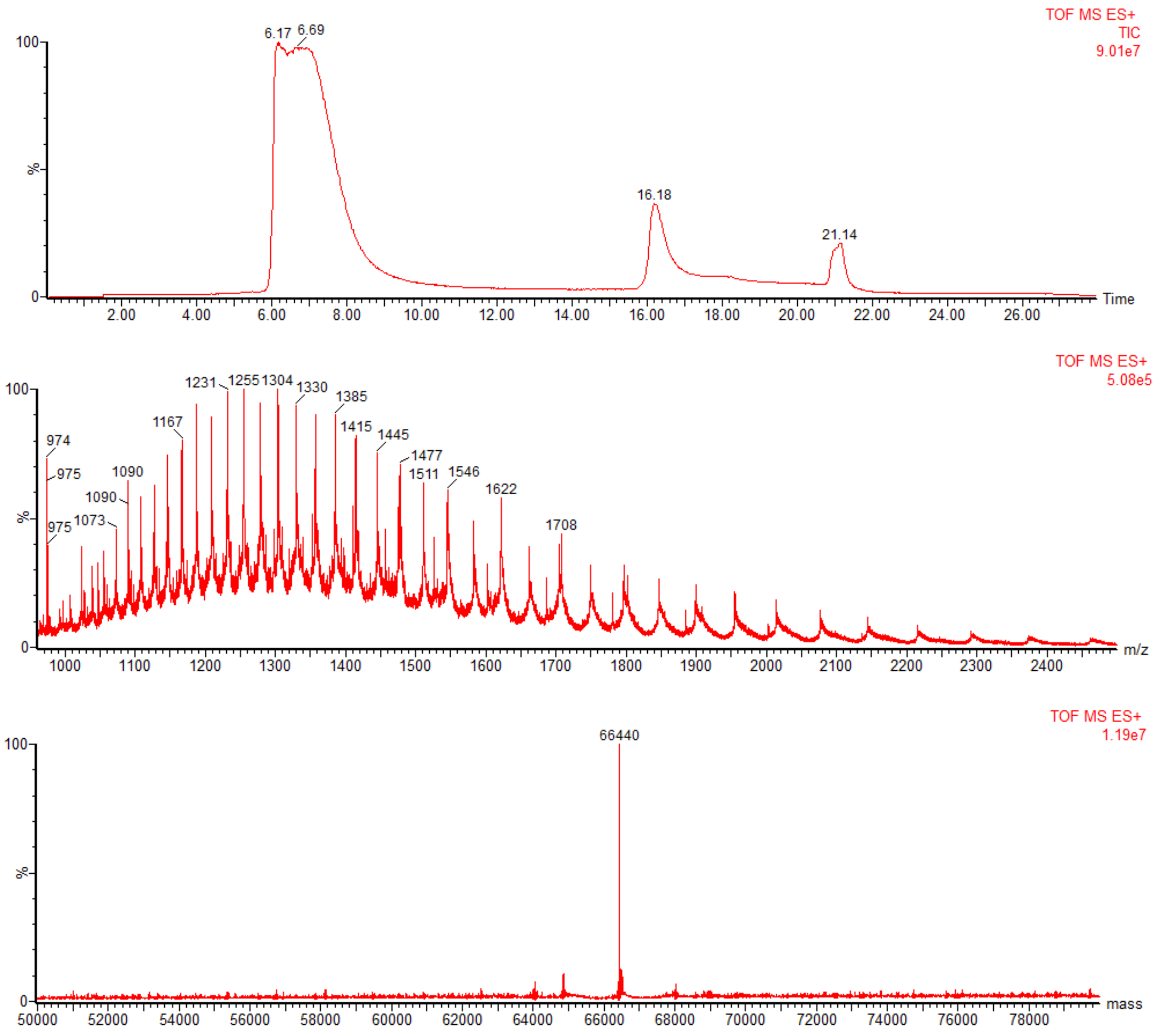

Supplementary Fig. 16 LC–MS analysis of albumin-Cys34. Total ion chromatogram, combined ion series and deconvoluted mass spectrum reconstructed from the ion series using the MaxEnt algorithm.

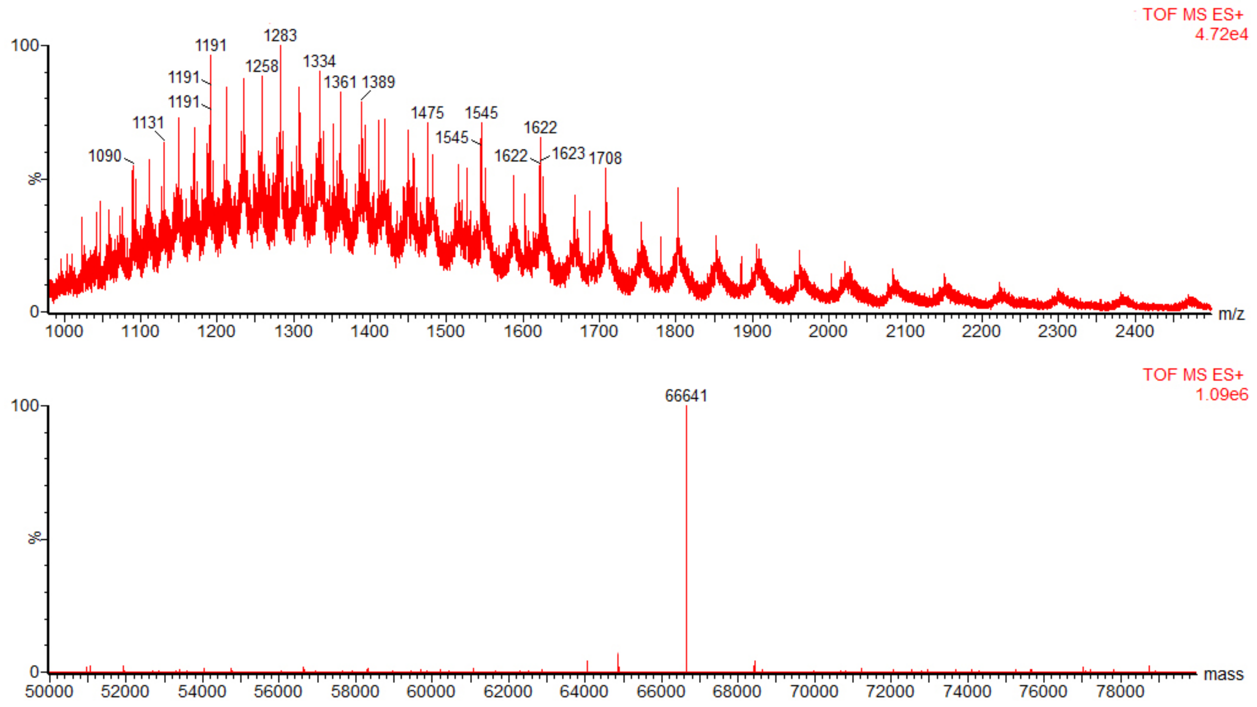

Supplementary Fig. 17 LC–MS analysis of the reaction of albumin with 2c after 2 h at RT. Combined ion series and deconvoluted mass spectrum reconstructed from the ion series using the MaxEnt algorithm.

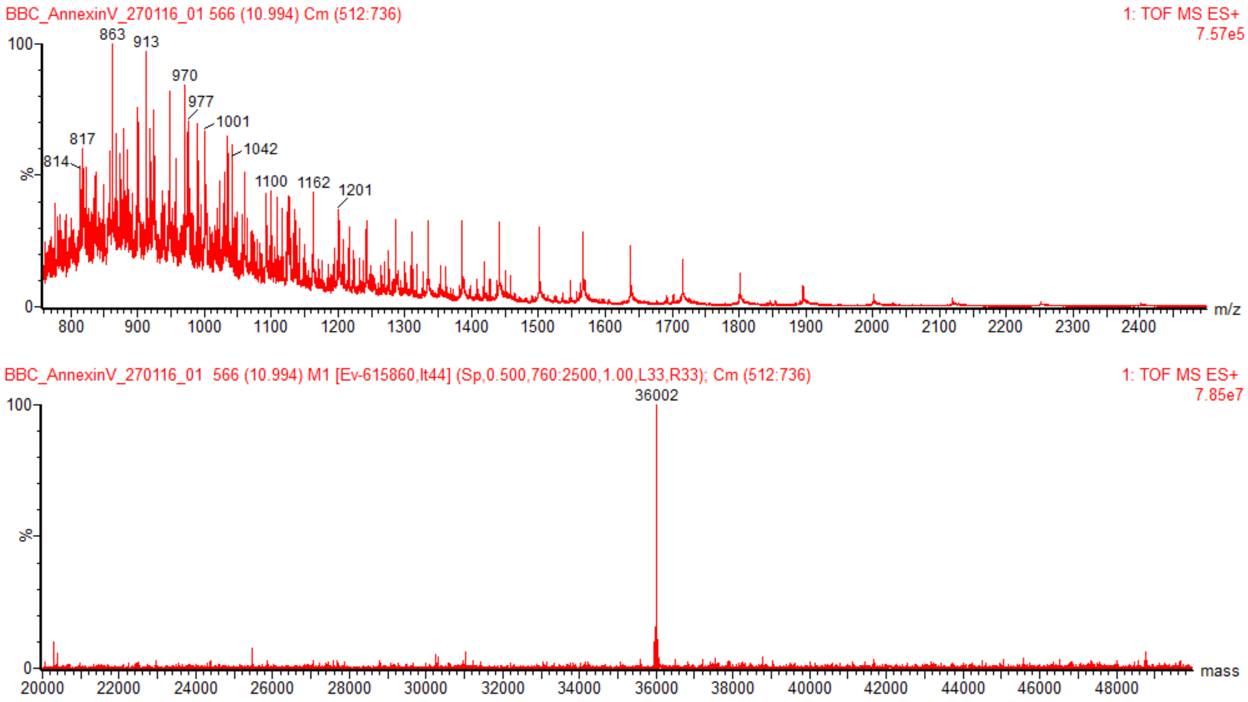

Supplementary Fig. 18 LC–MS analysis of the reaction of Annexin-Cys315 with Ellman's reagent after 4 h at 37 ºC. Combined ion series and deconvoluted mass spectrum reconstructed from the ion series using the MaxEnt algorithm.

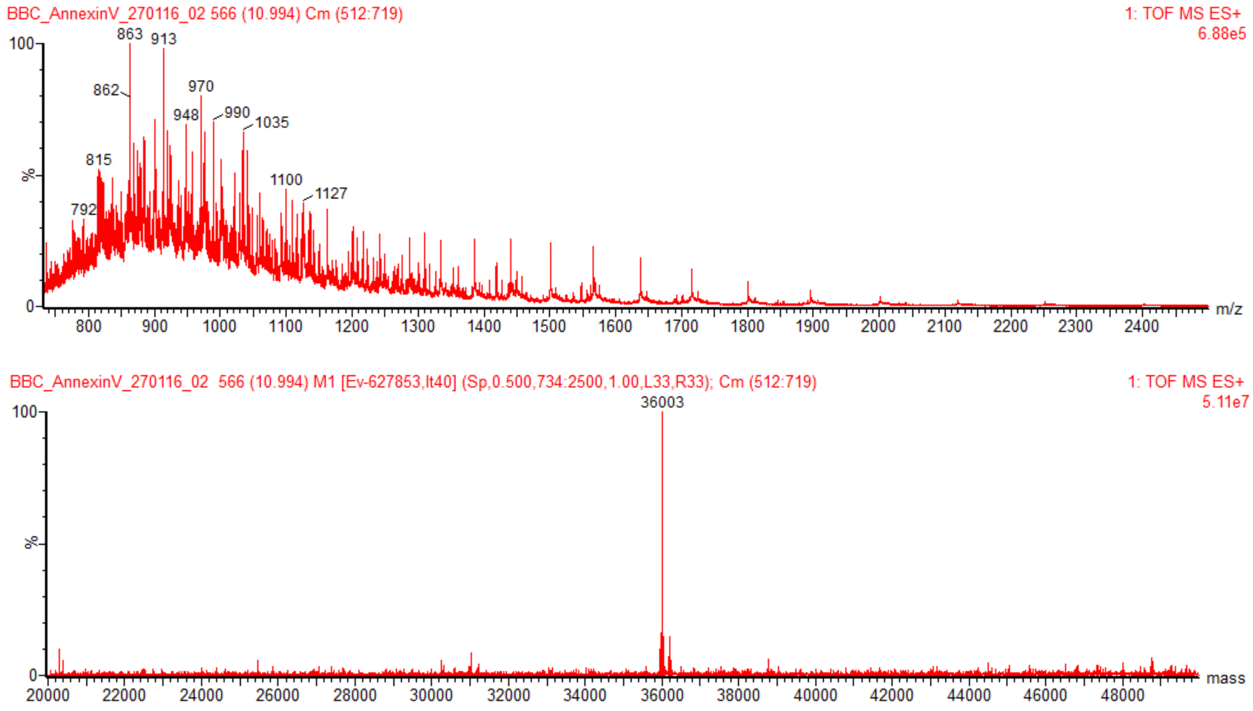

Supplementary Fig. 19 LC–MS analysis of the reaction of Annexin V-SS-Ellman's with 2c after 1 h at 37 ºC. No reaction was observed. Combined ion series and deconvoluted mass spectrum reconstructed from the ion series using the MaxEnt algorithm.

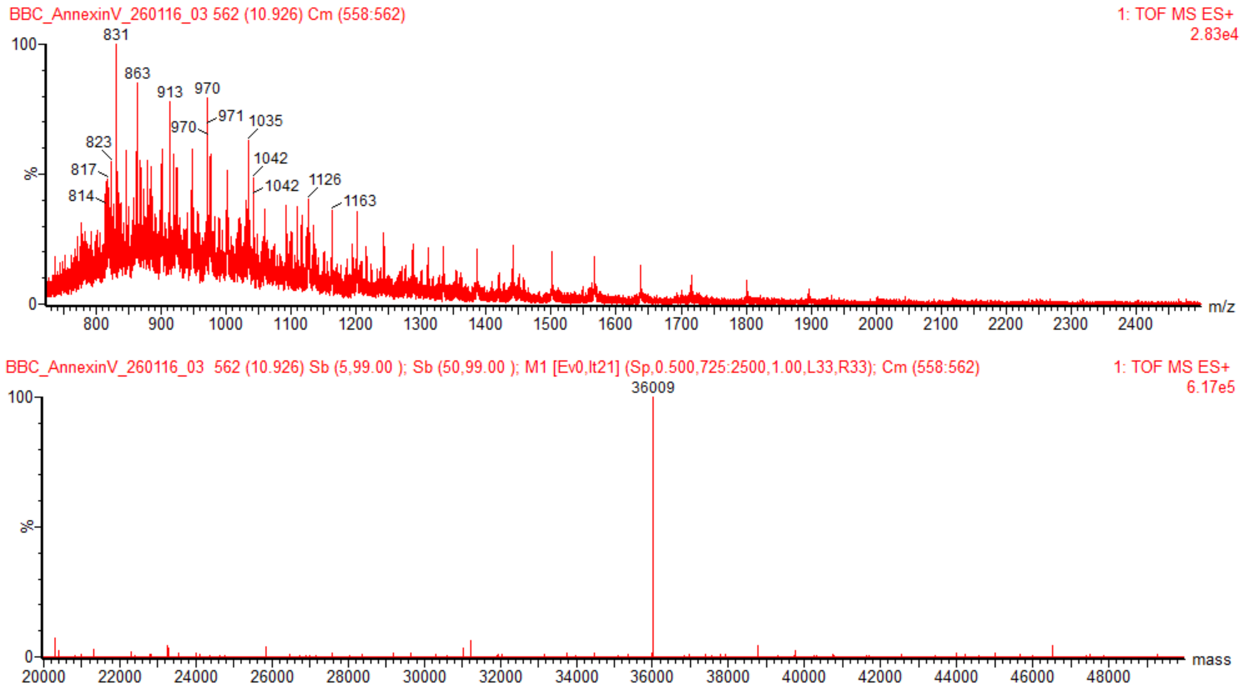

Supplementary Fig. 20 LC–MS analysis of the reaction of the conjugate Annexin V-2c with Ellman's reagent after 1 h at 37 ºC. No reaction was observed. Combined ion series and deconvoluted mass spectrum reconstructed from the ion series using the MaxEnt algorithm.

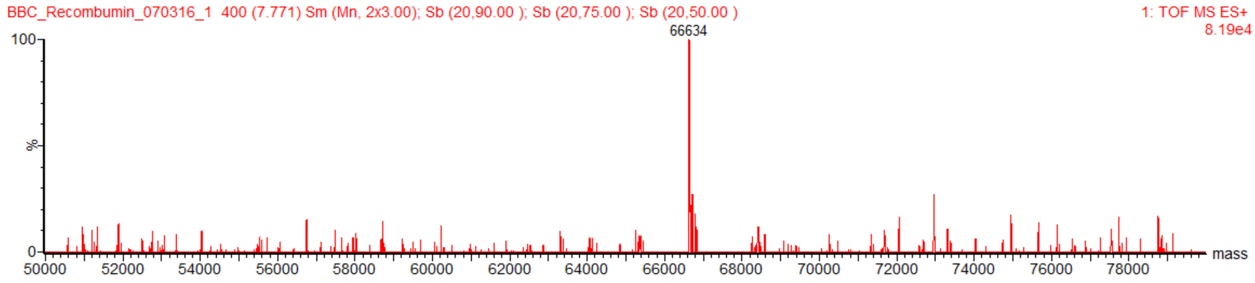

Supplementary Fig. 21 Stability of the conjugate albumin-2c in human plasma. Reaction of albumin-**2c** in the presence of human plasma after 48 h at 37 ºC. Deconvoluted mass spectrum reconstructed from the ion series using the MaxEnt algorithm.

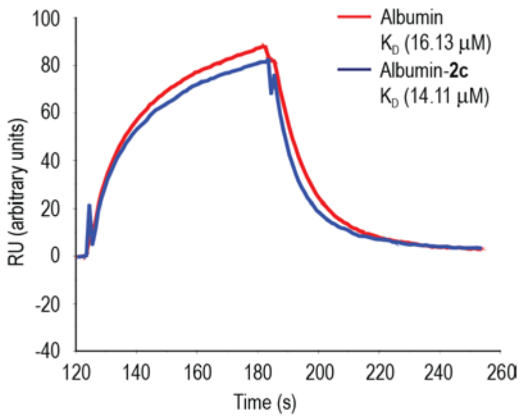

Supplementary Fig. 22 Assessment of the FcRn binding properties of albumin-2c. Biacore SPR assessment of human FcRn binding of albumins at 10 μM.

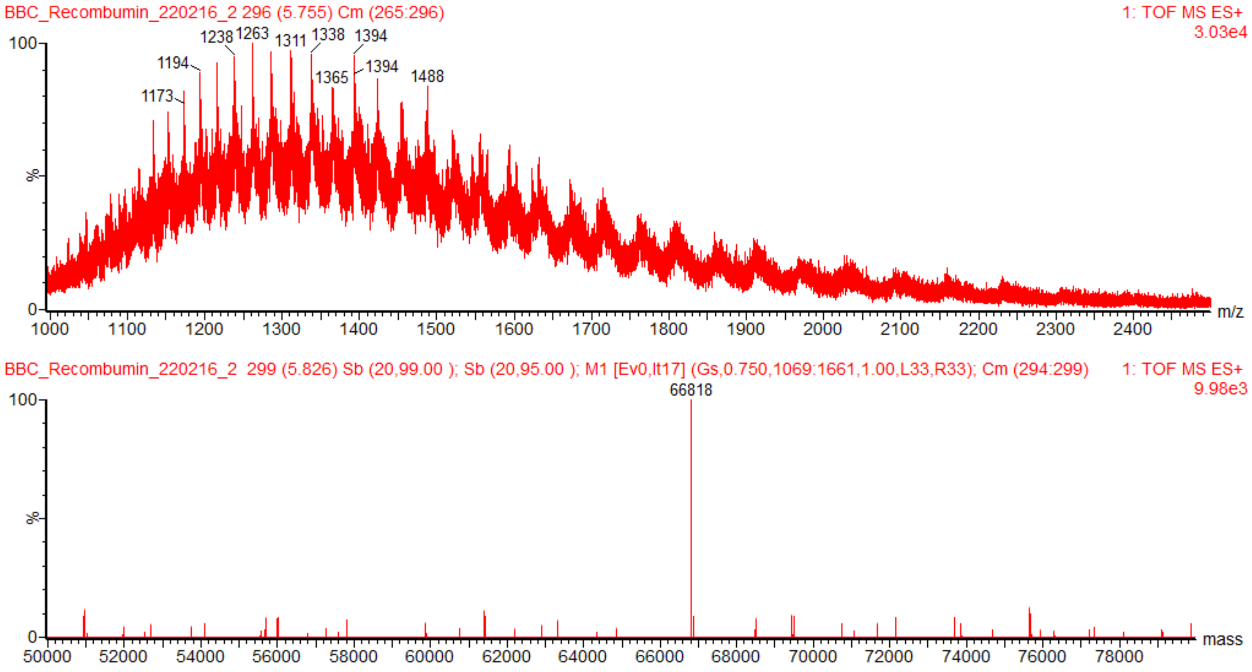

Supplementary Fig. 23 Labelling of albumin with the fluorescent reagent 7.

Reaction of albumin-Cys34 with fluorescent reagent **7** after 2 h at RT. Deconvoluted mass spectrum reconstructed from the ion series using the MaxEnt algorithm.

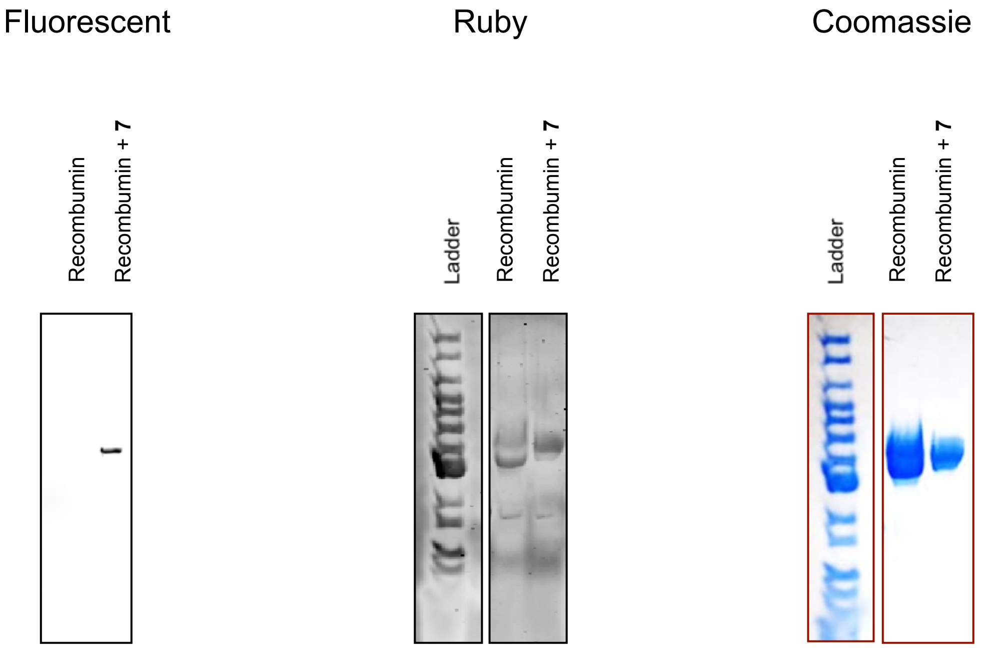

Supplementary Fig. 24 SDS-PAGE analysis of albumin-7. NuPAGE® Novex 4–12% Bis-Tris Mini Gel with MES buffer. Each lane contains 5 µL Mark12™ Unstained Standard.

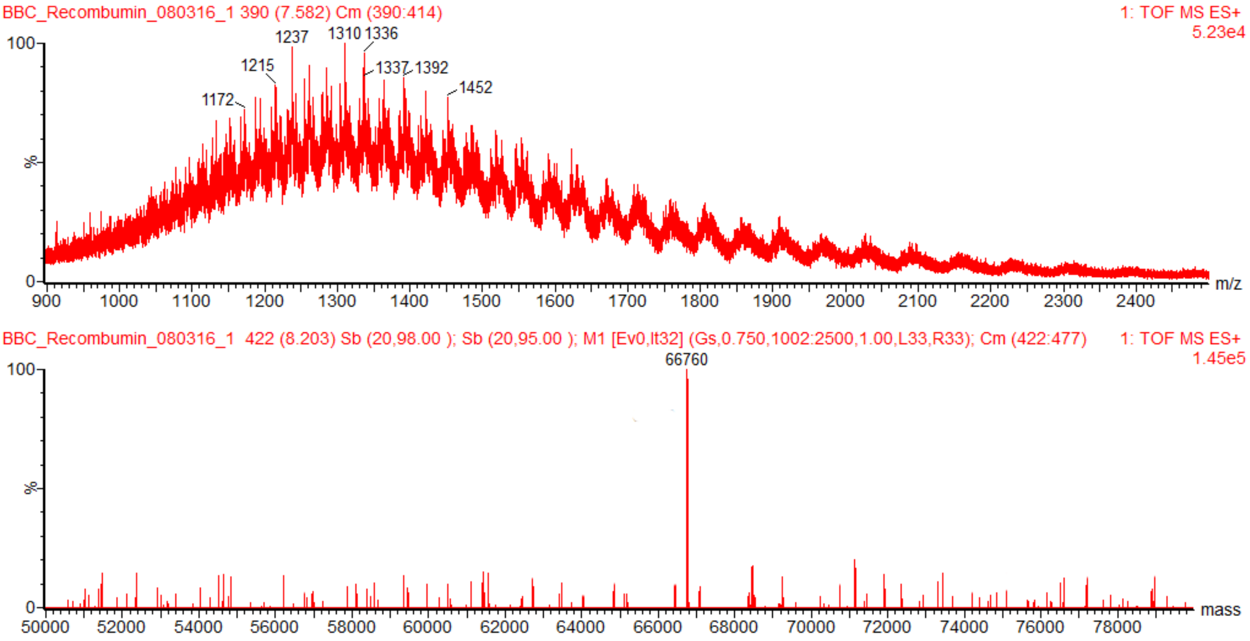

Supplementary Fig. 25 PEGylation of albumin with 8. Reaction of albumin-Cys34 with PEG reagent **8** after 2 h at RT. Deconvoluted mass spectrum reconstructed from the ion series using the MaxEnt algorithm.

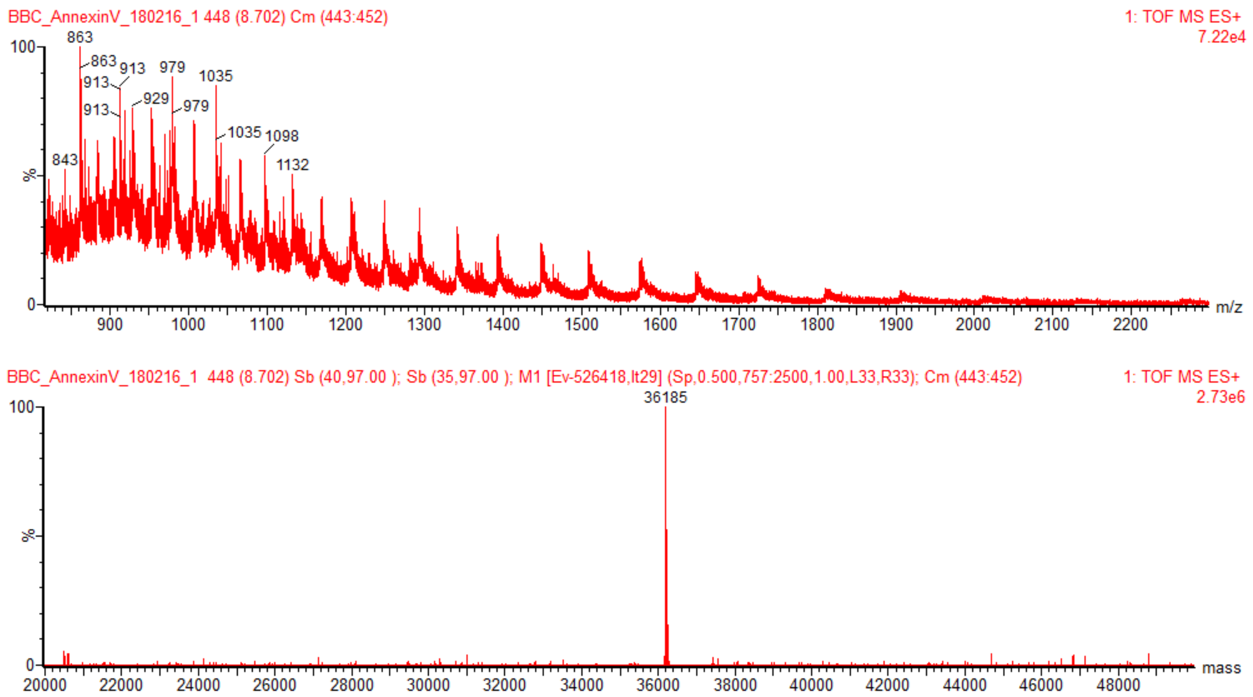

Supplementary Fig. 26 Labelling of Annexin V-Cys315 with fluorescent reagent 7. Reaction of Annexin V-Cys315 protein with **7** after 1 h at 37 ºC. Deconvoluted mass spectrum reconstructed from the ion series using the MaxEnt algorithm.

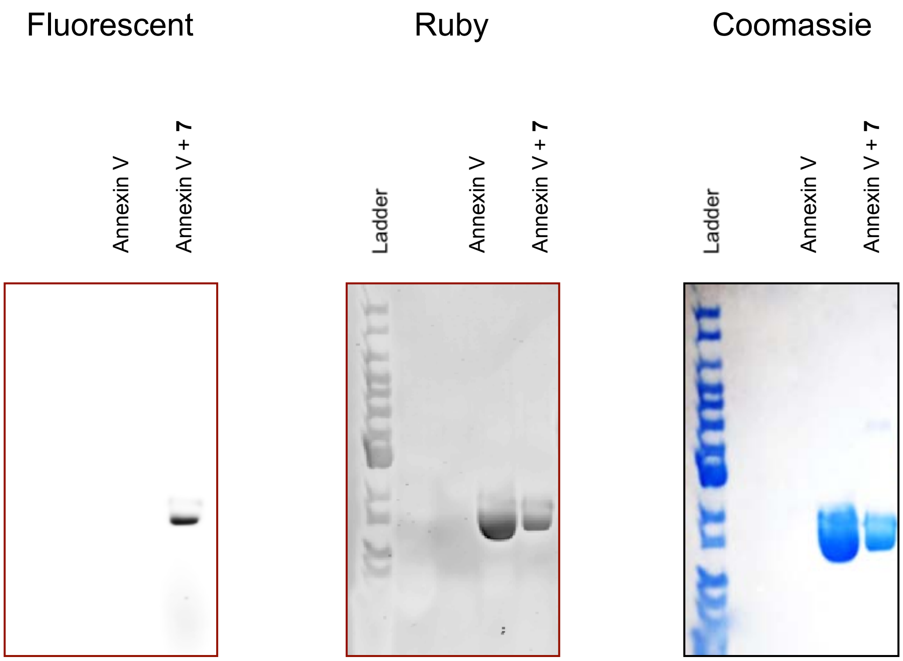

Supplementary Fig. 27 SDS-PAGE analysis of Annexin V-7. NuPAGE® Novex 4–12% Bis-Tris Mini Gel with MES buffer. Each lane contains 5 μL Mark12™ Unstained Standard.

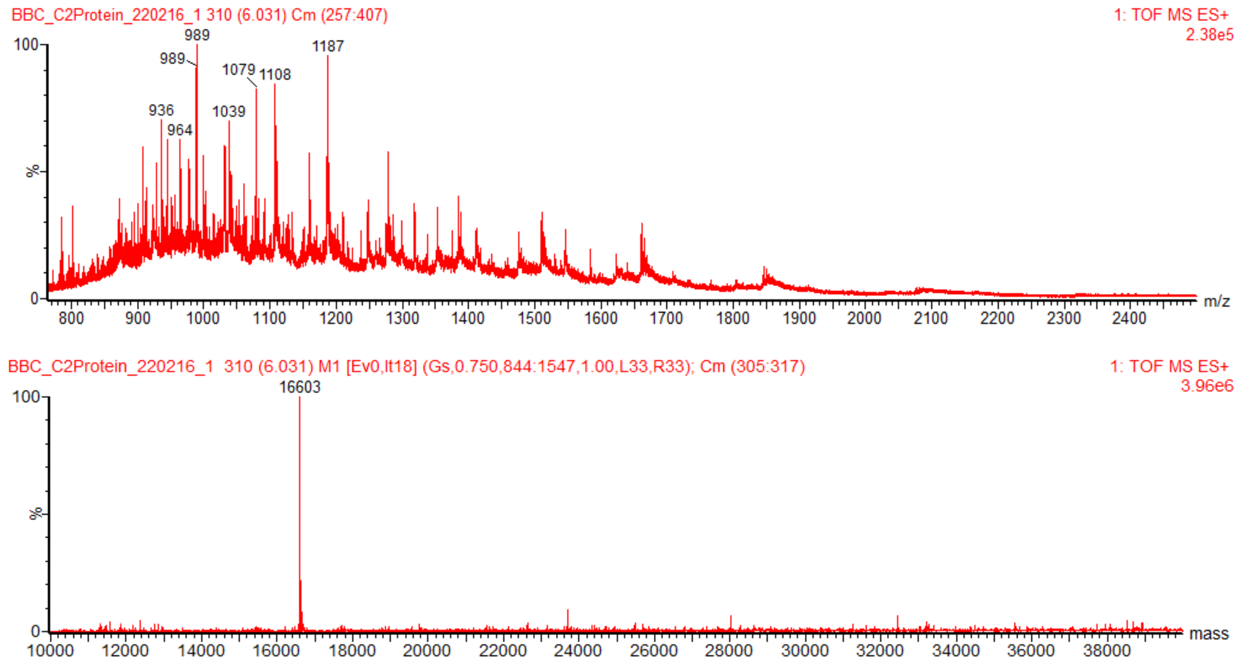

Supplementary Fig. 28 Labelling of C2Am with fluorescent reagent 7.
Reaction of C2Am-Cys95 protein with **7** after 2 h at RT. Deconvoluted mass spectrum reconstructed from the ion series using the MaxEnt algorithm.

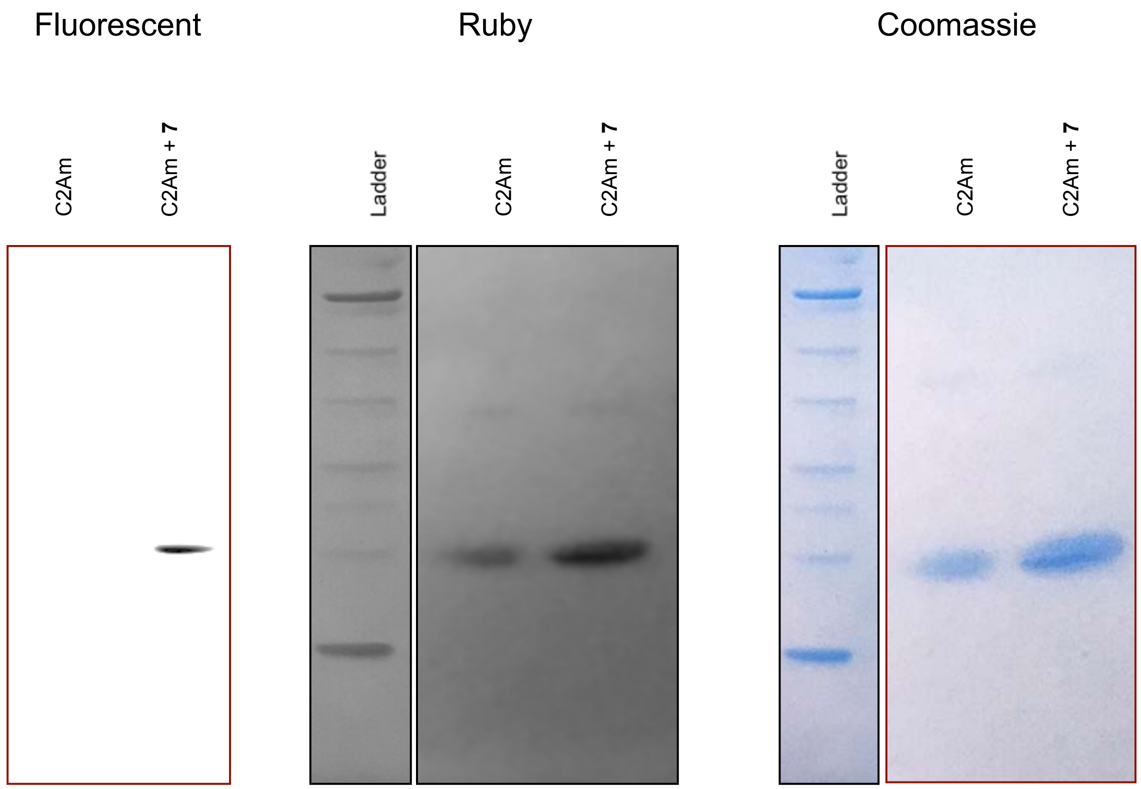

Supplementary Fig. 29 SDS-PAGE analysis of C2Am-7. NuPAGE® Novex 4–12% Bis-Tris Mini Gel with MES buffer. Each lane contains 5 µL Mark12™ Unstained Standard.

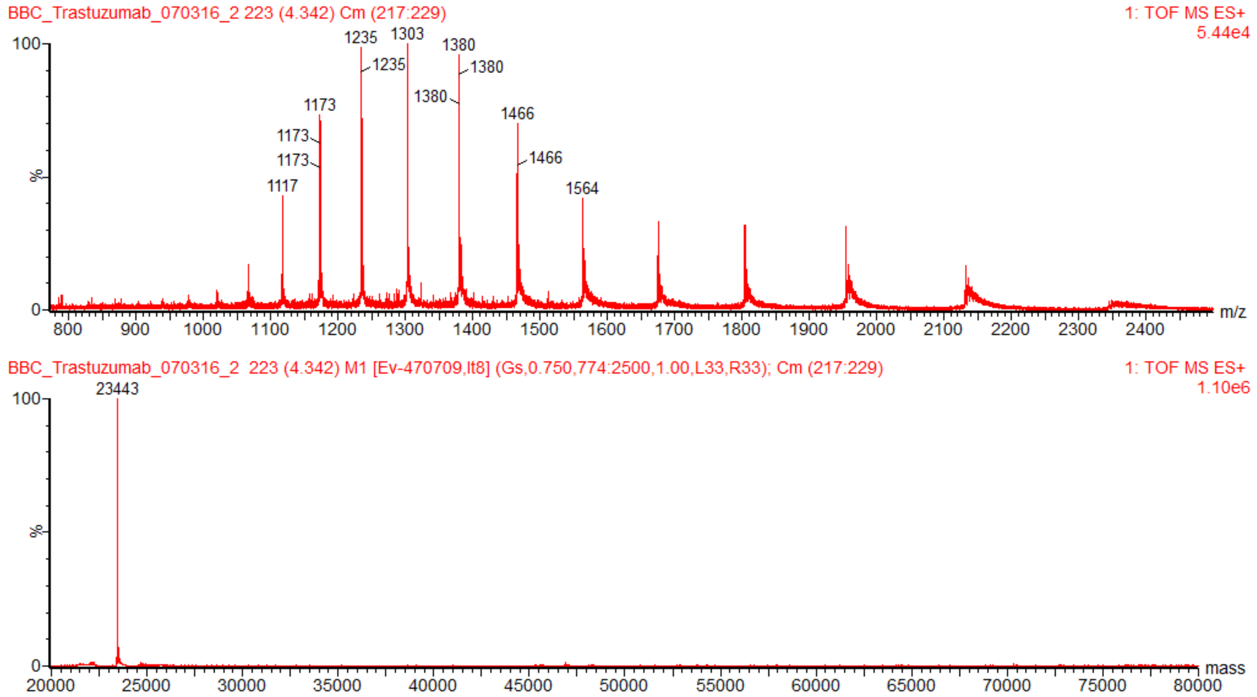

Supplementary Fig. 30 Disulfide reduction of Trastuzumab. Combined ion series and deconvoluted mass spectrum reconstructed from the ion series using the MaxEnt algorithm of the light-chain of Trastuzumab after reaction with 10 equiv. of TCEP after 1.5 h at RT.

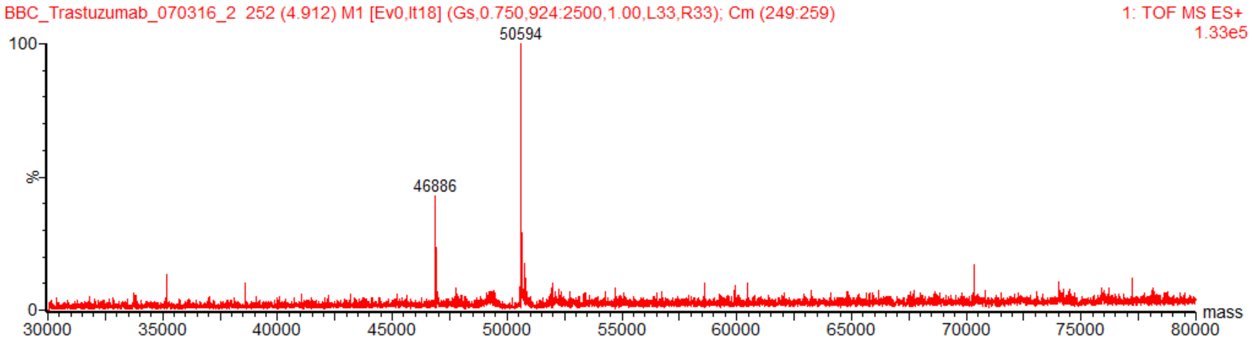

Supplementary Fig. 31 Disulfide reduction of Trastuzumab. Deconvoluted mass spectrum reconstructed from the ion series using the MaxEnt algorithm of the heavy-chain of Trastuzumab after reaction with 10 equiv. of TCEP after 1.5 h at RT.

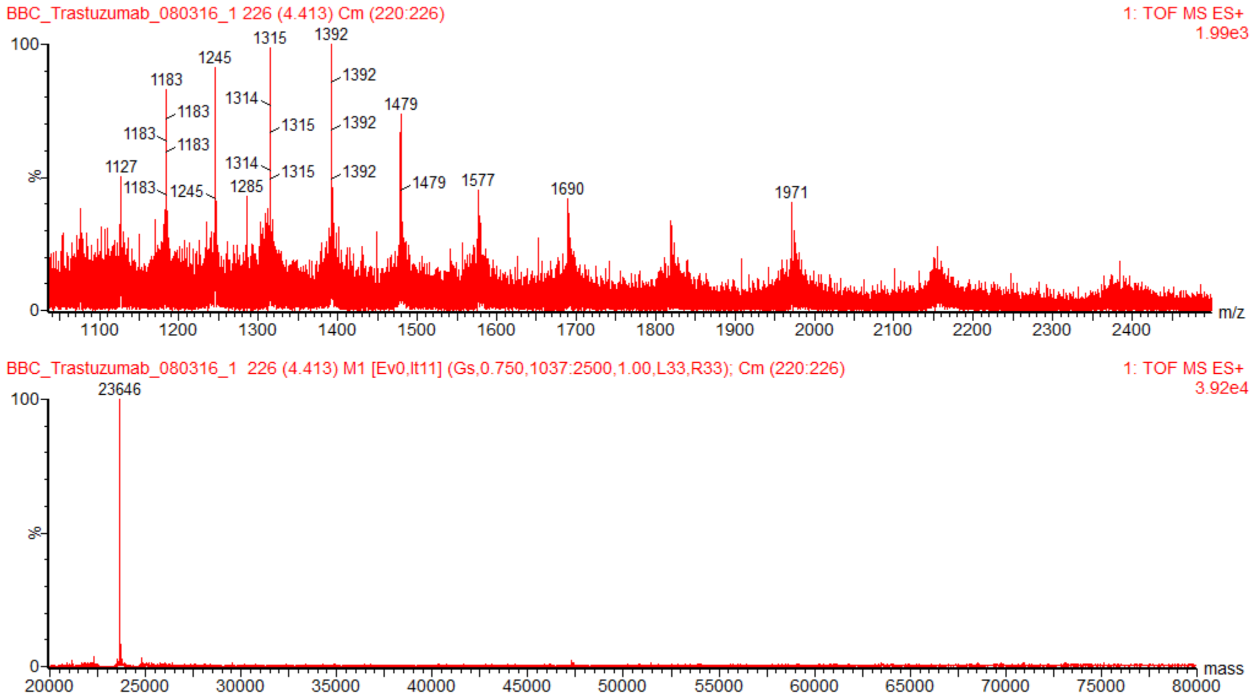

Supplementary Fig. 32 Bioconjugation of 2c to Trastuzumab. Combined ion series and deconvoluted mass spectrum reconstructed from the ion series using the MaxEnt algorithm of the light-chain of Trastuzumab after reaction with **2c** for 1 h at RT.

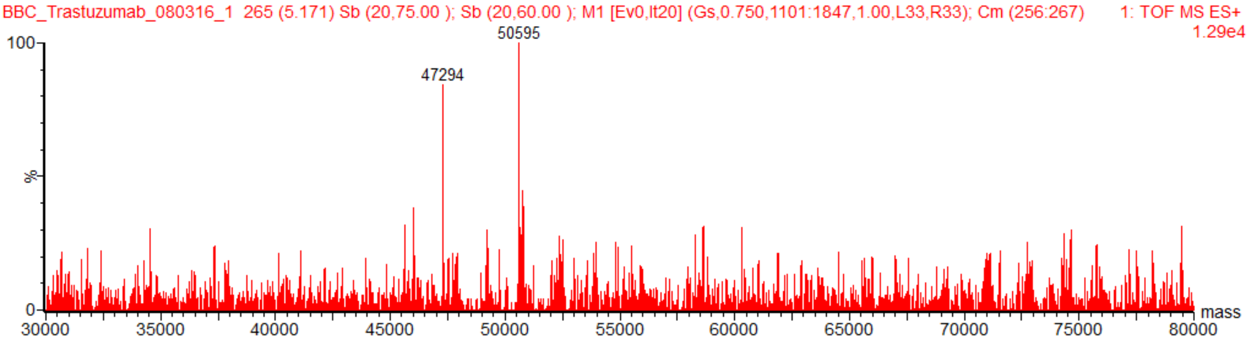

Supplementary Fig. 33 Bioconjugation of 2c to Trastuzumab. Deconvoluted mass spectrum reconstructed from the ion series using the MaxEnt algorithm of the heavy-chain of Trastuzumab after reaction with **2c** for 1 h at RT

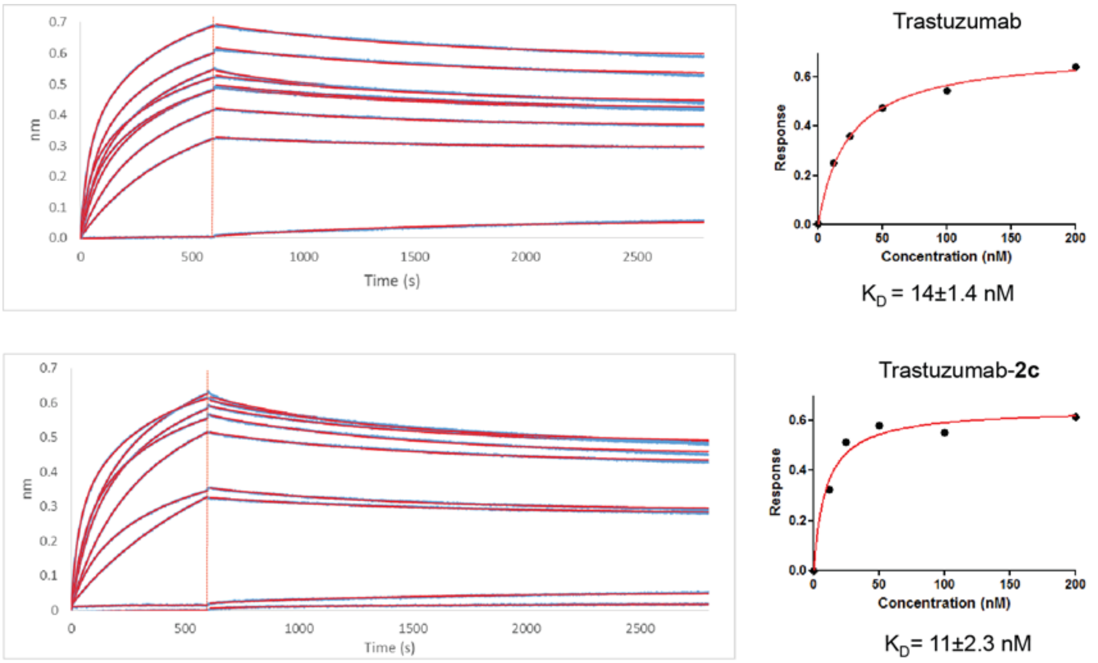

Supplementary Fig. 34 Determination of antibody conjugate binding constant. Bio-layer interferometry (BLI) curves (in blue) and fitting curves (in red) obtained for Trastuzumab and Trastuzumab-**2c** with HER2 Receptor, together with the K_D constants derived from BLI experiments.

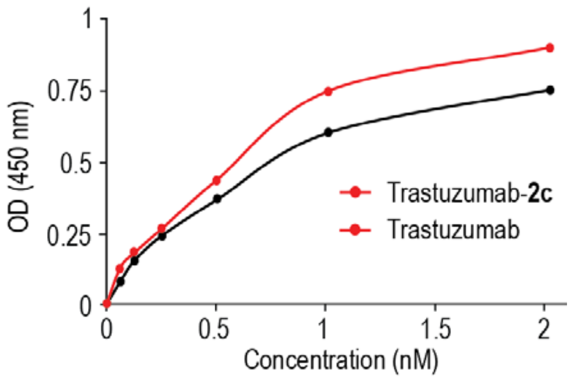

Supplementary Fig. 35 ELISA assessment of binding activity. Binding activity data for Trastuzumab and conjugate Trastuzumab-**2** determined using ELISA.

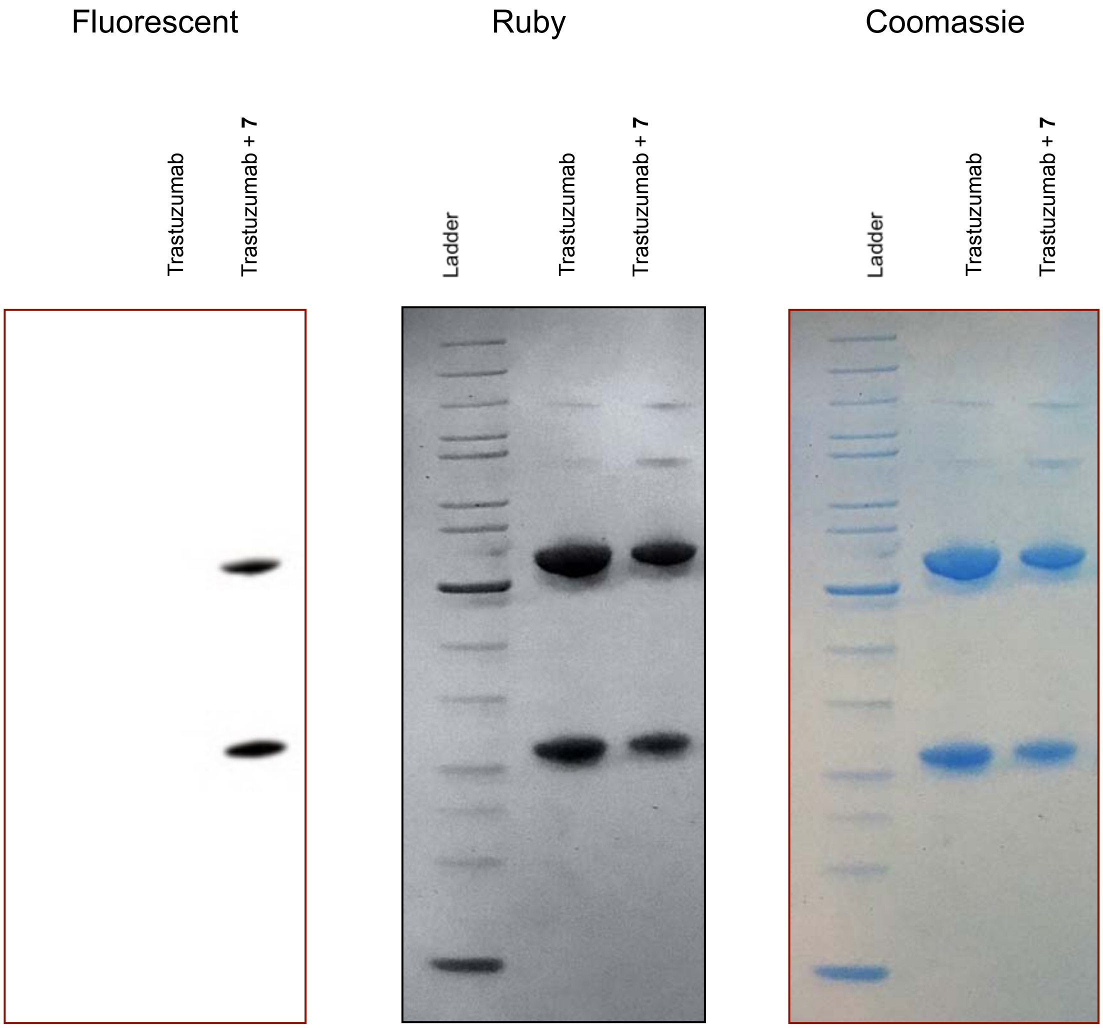

Supplementary Fig. 36 SDS-PAGE analysis of Trastuzumab-7. NuPAGE® Novex 4–12% Bis-Tris Mini Gel with MES buffer. Each lane contains 5 μL Mark12™ Unstained Standard.

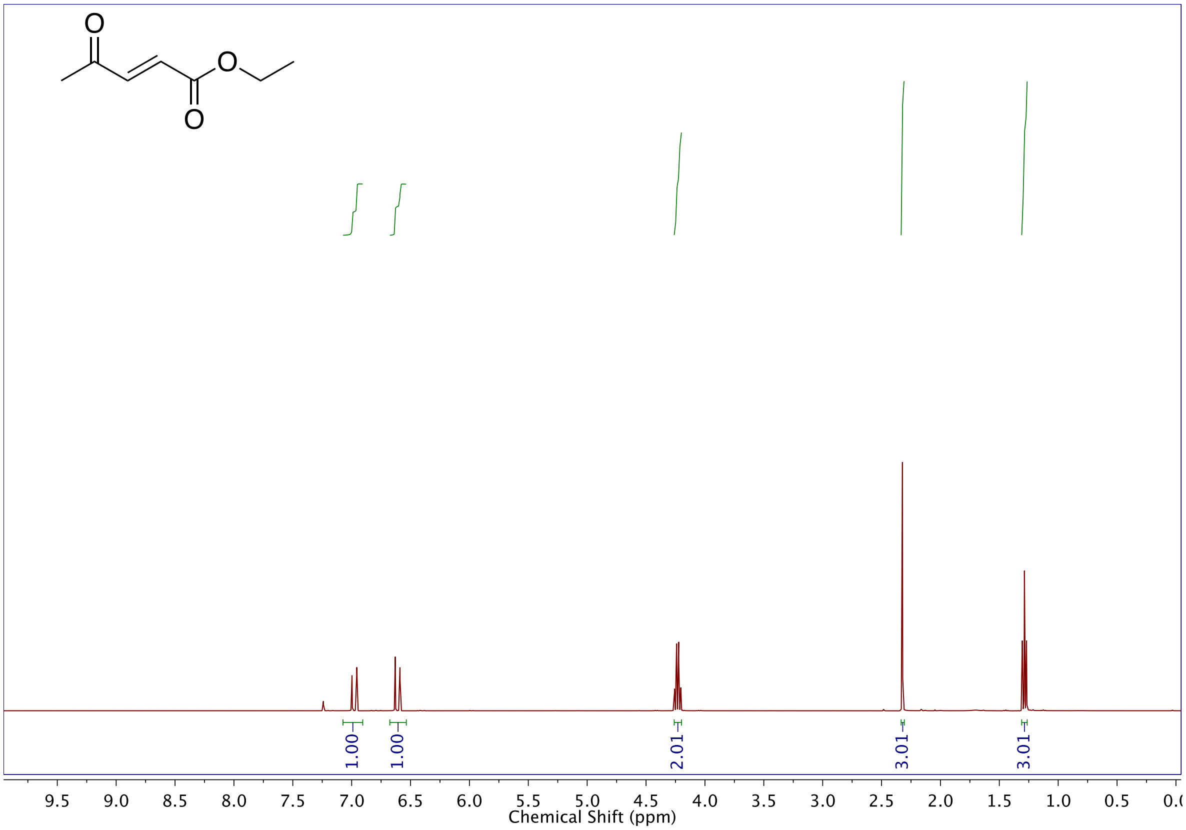

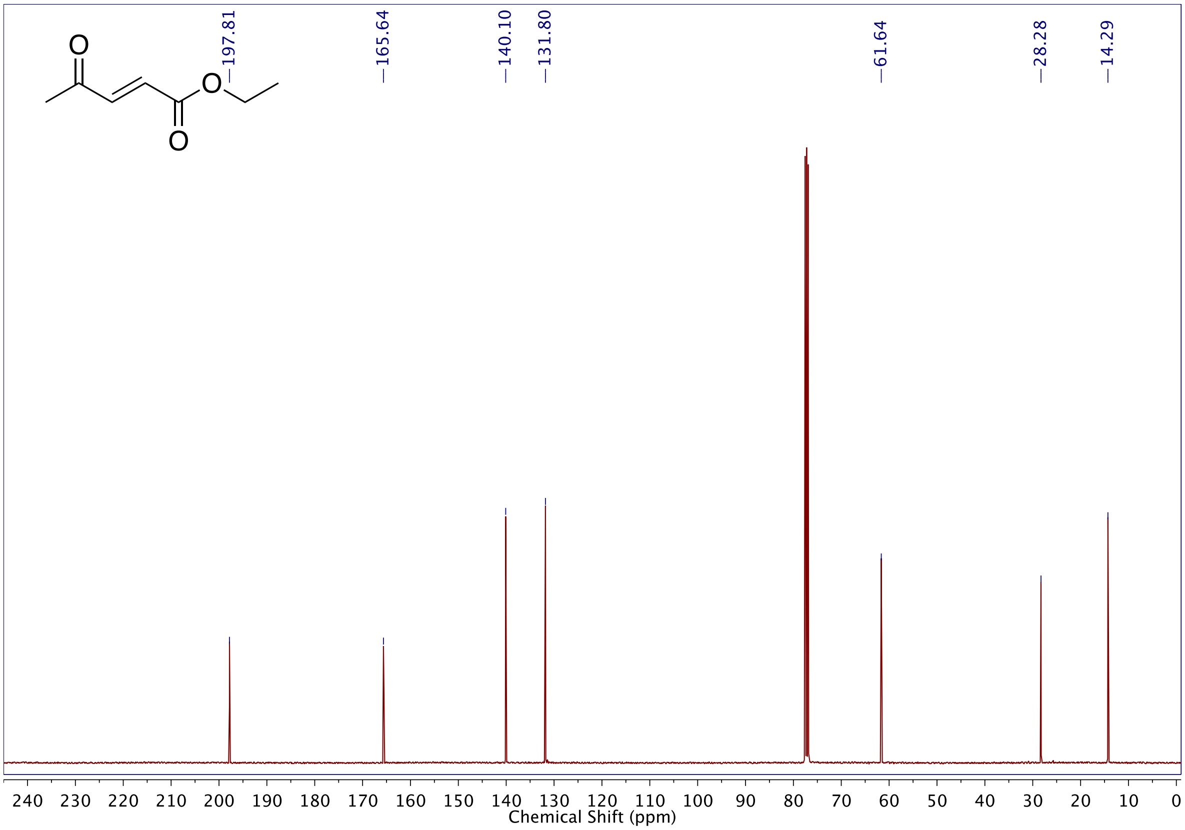

Supplementary Fig. 37 ^{1}H and ^{13}C spectra of compound **2b**.

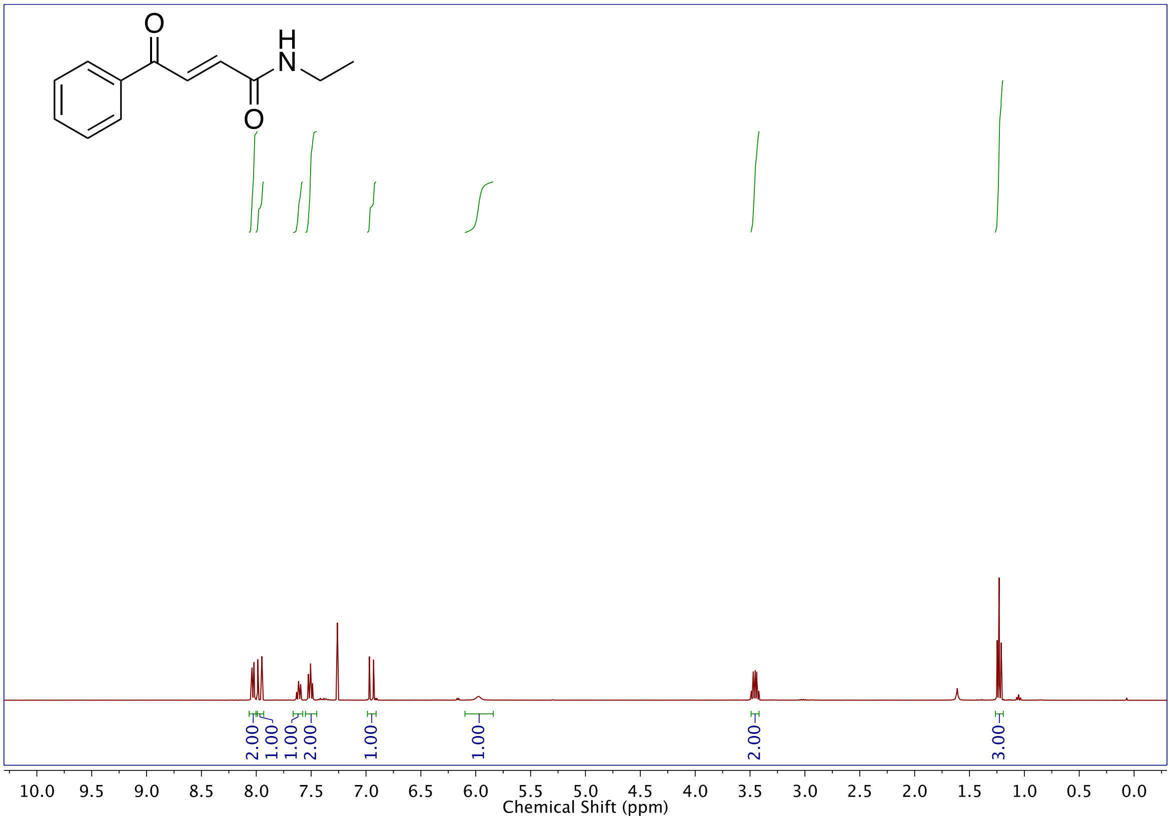

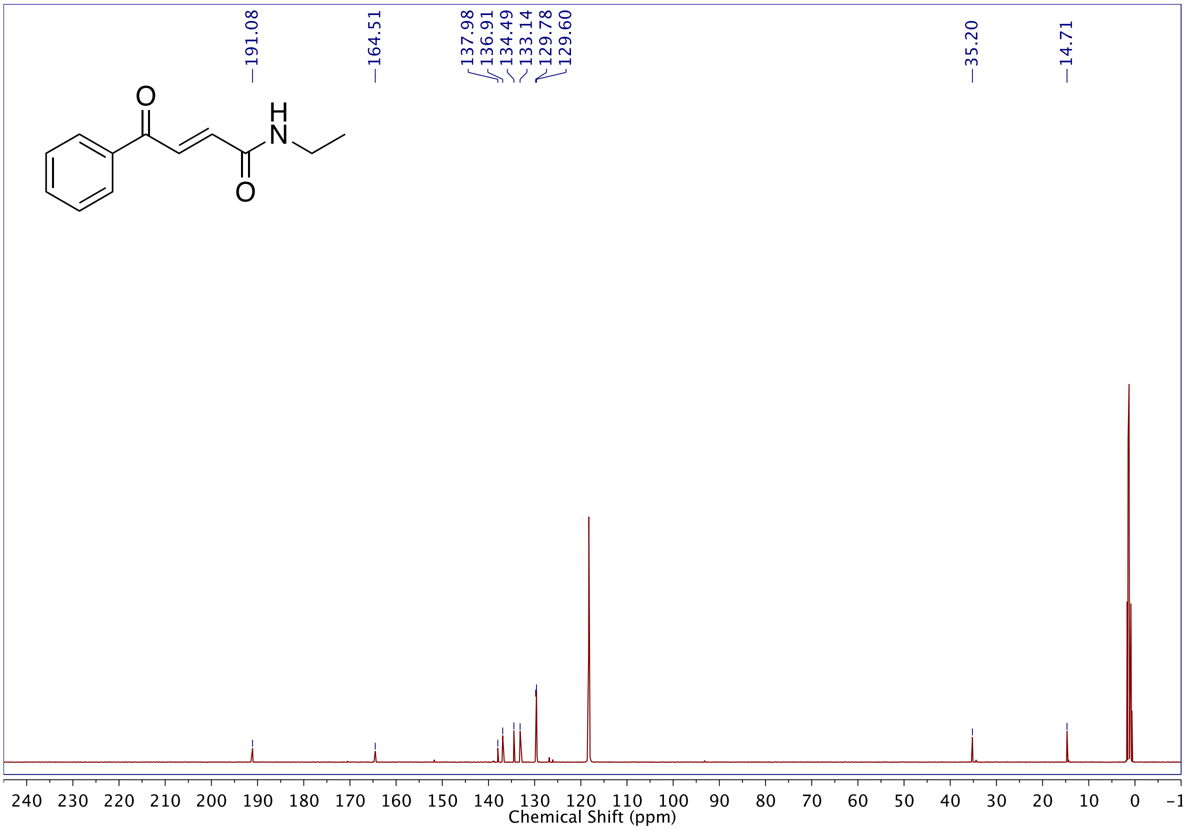

Supplementary Fig. 38 ^{1}H and ^{13}C spectra of compound **2c**.

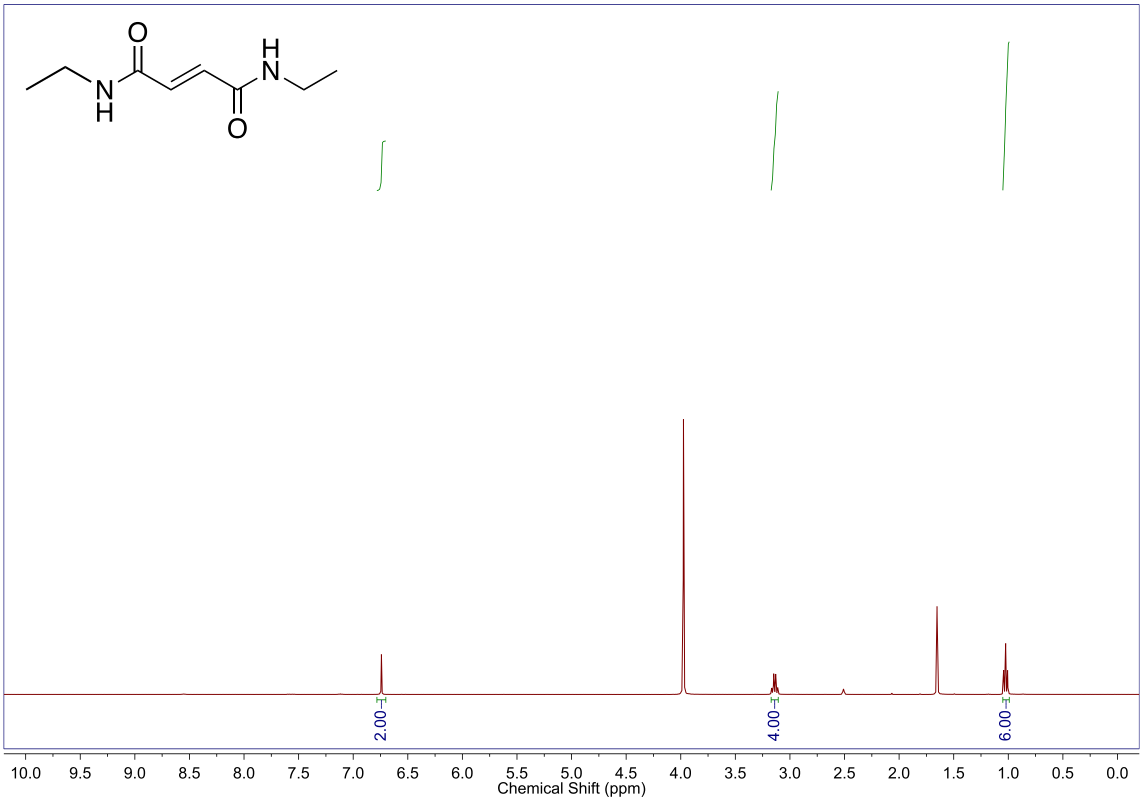

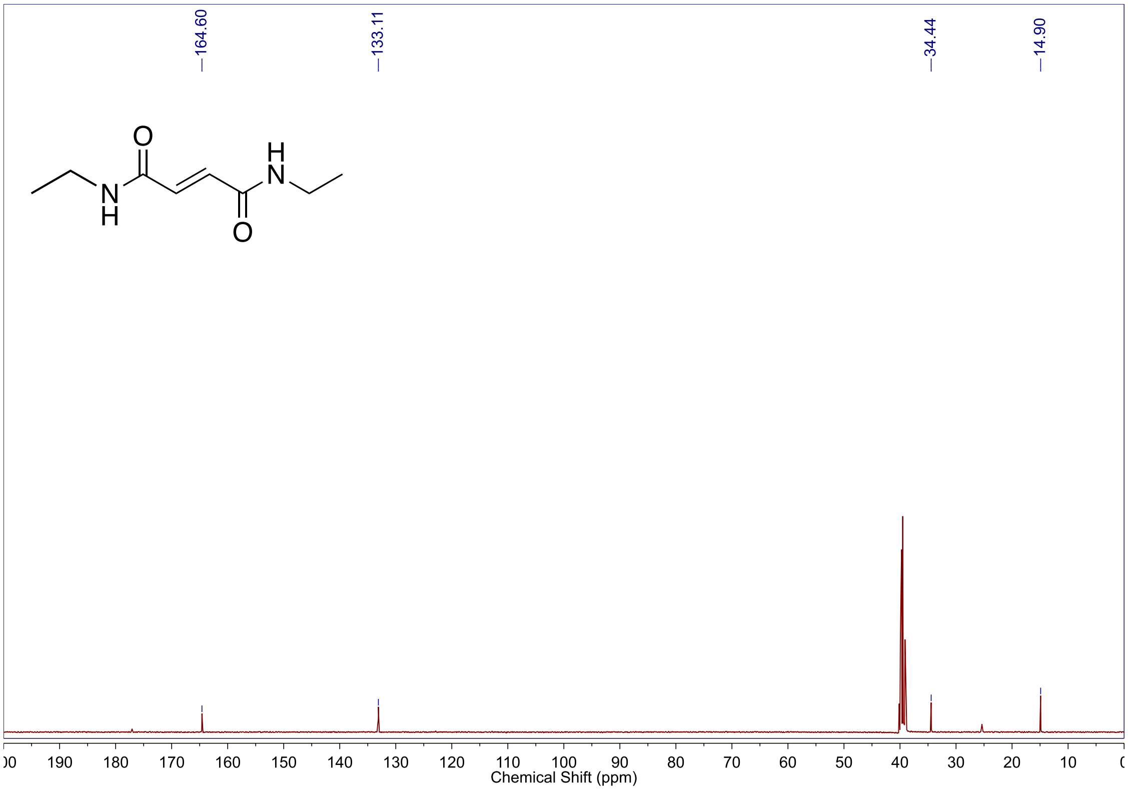

Supplementary Fig. 39 ^{1}H and ^{13}C spectra of compound **2d**.

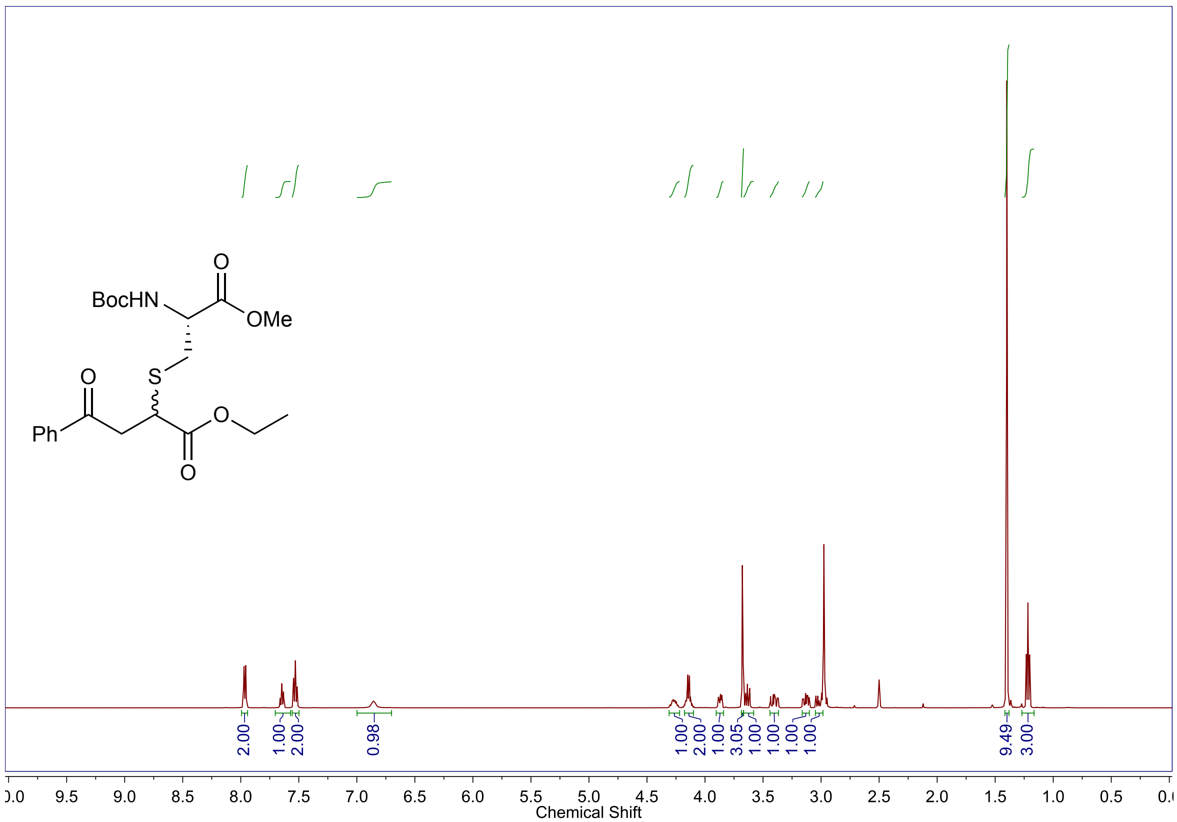

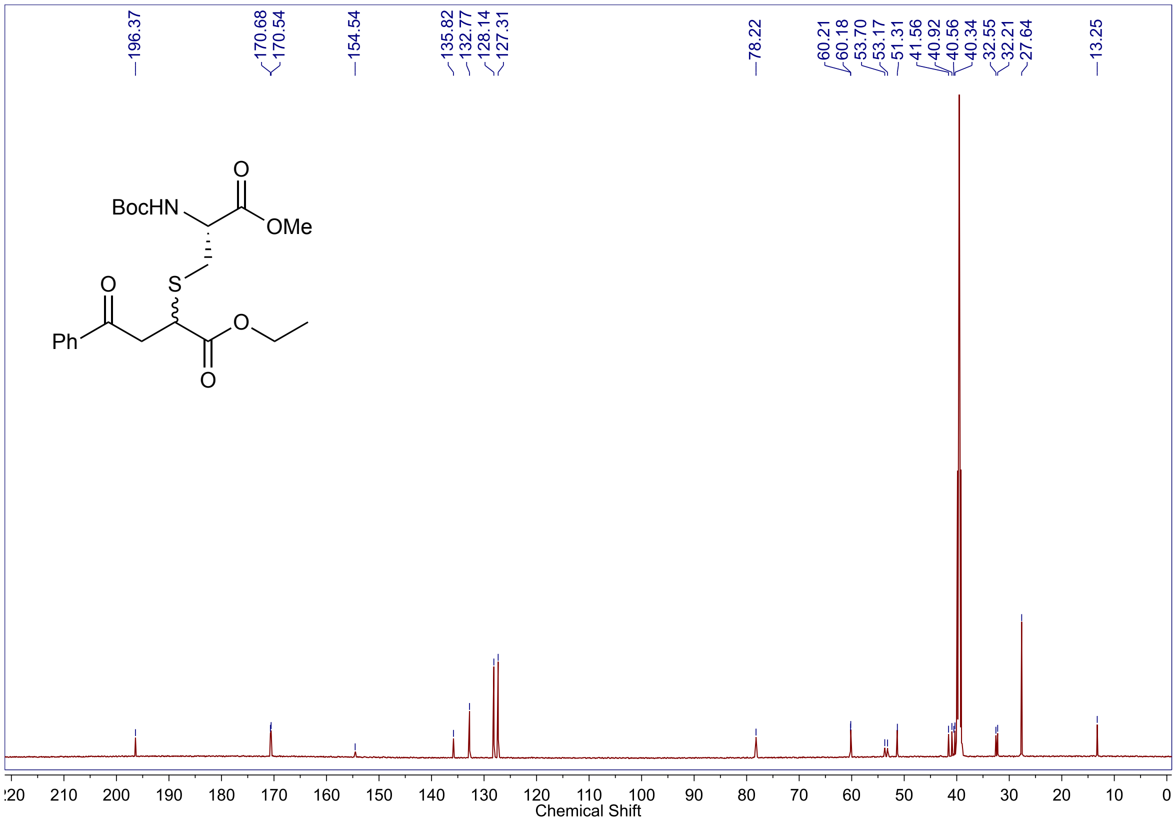

Supplementary Fig. 40 ^1H and ^{13}C spectra of compound **3a**.

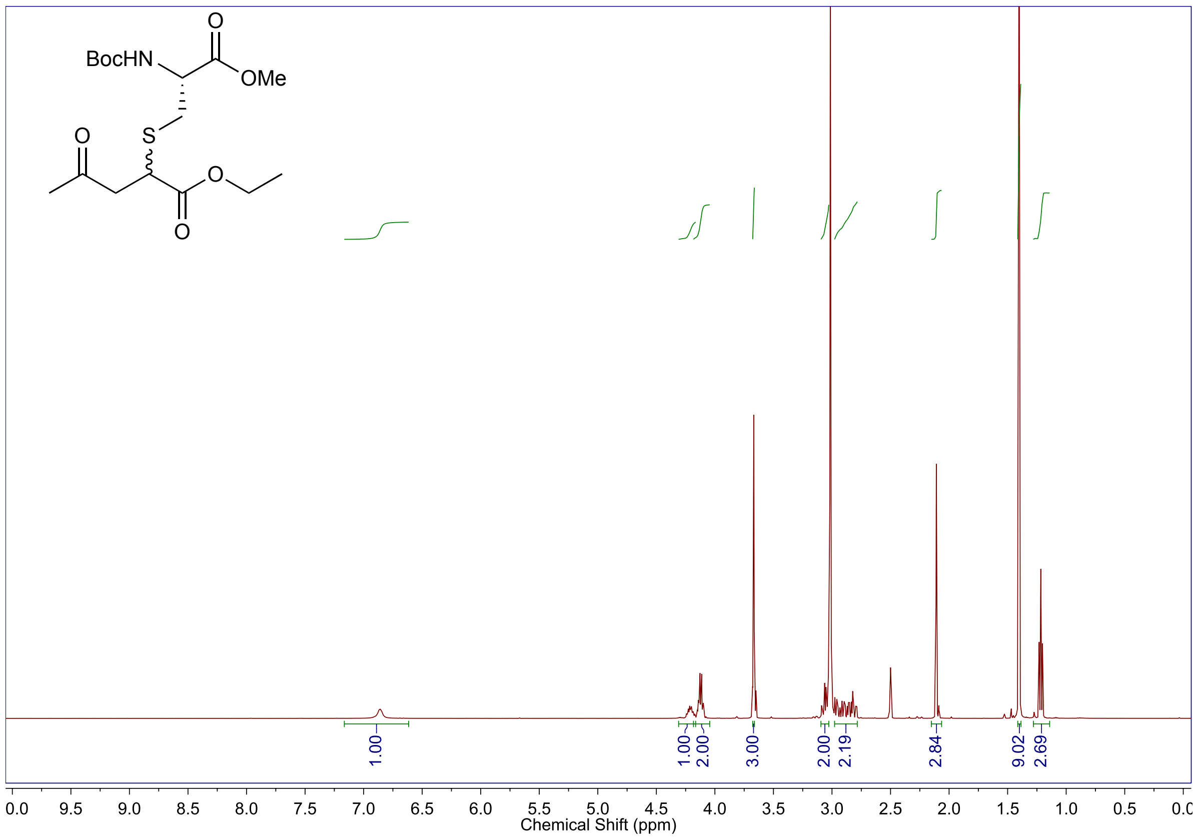

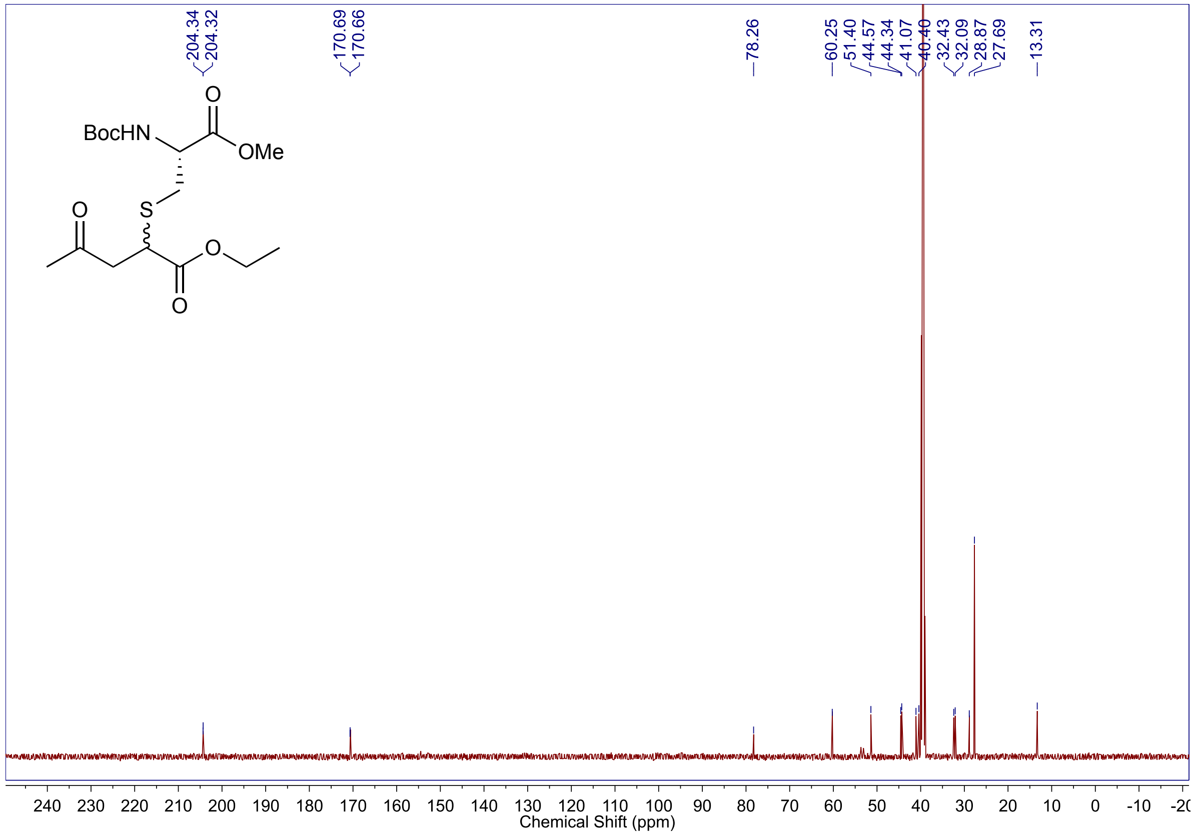

Supplementary Fig. 41 ^{1}H and ^{13}C spectra of compound **3b**.

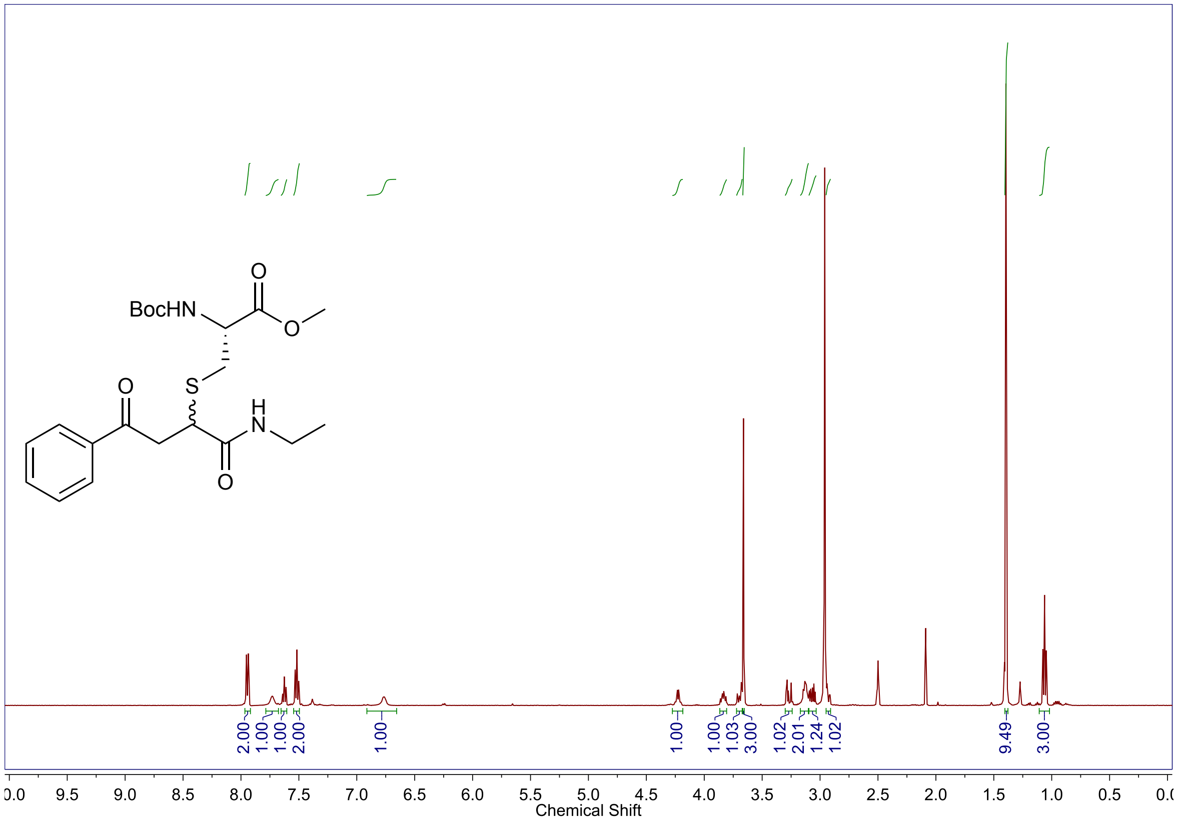

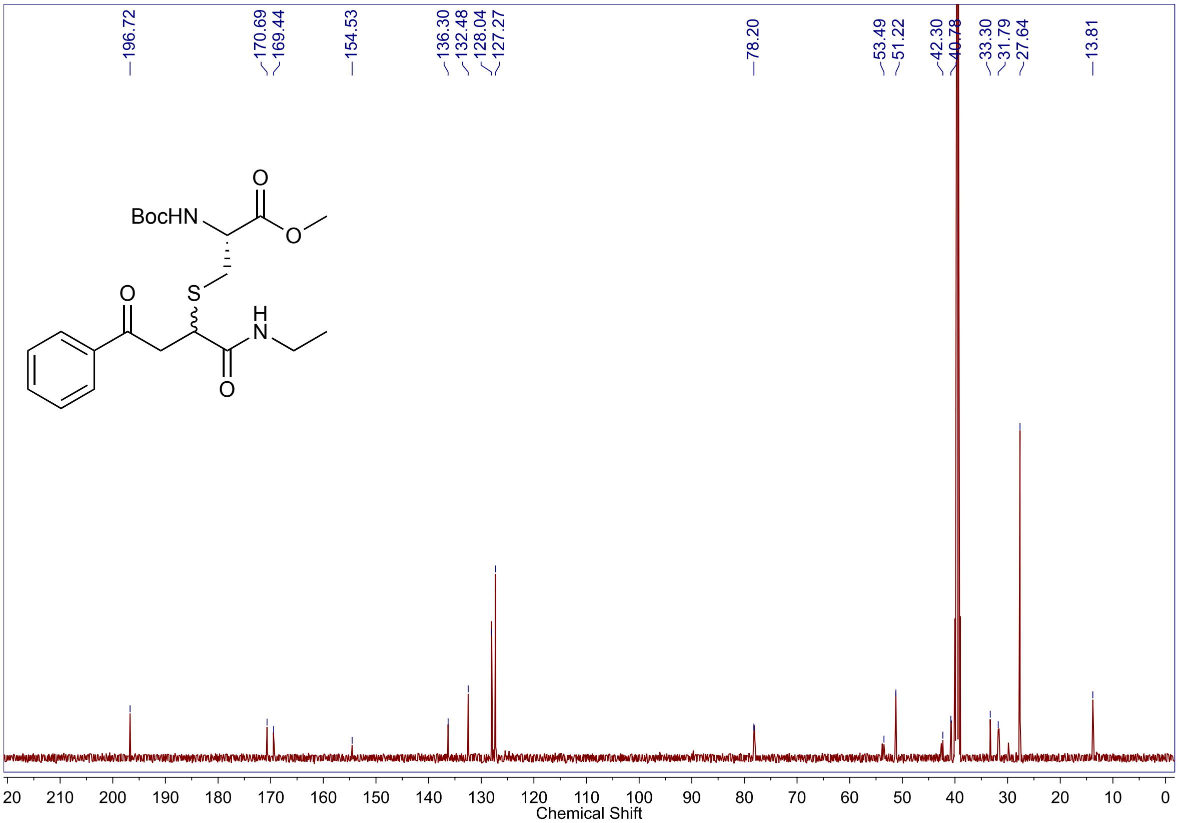

Supplementary Fig. 42 ^{1}H and ^{13}C spectra of compound **3c**.

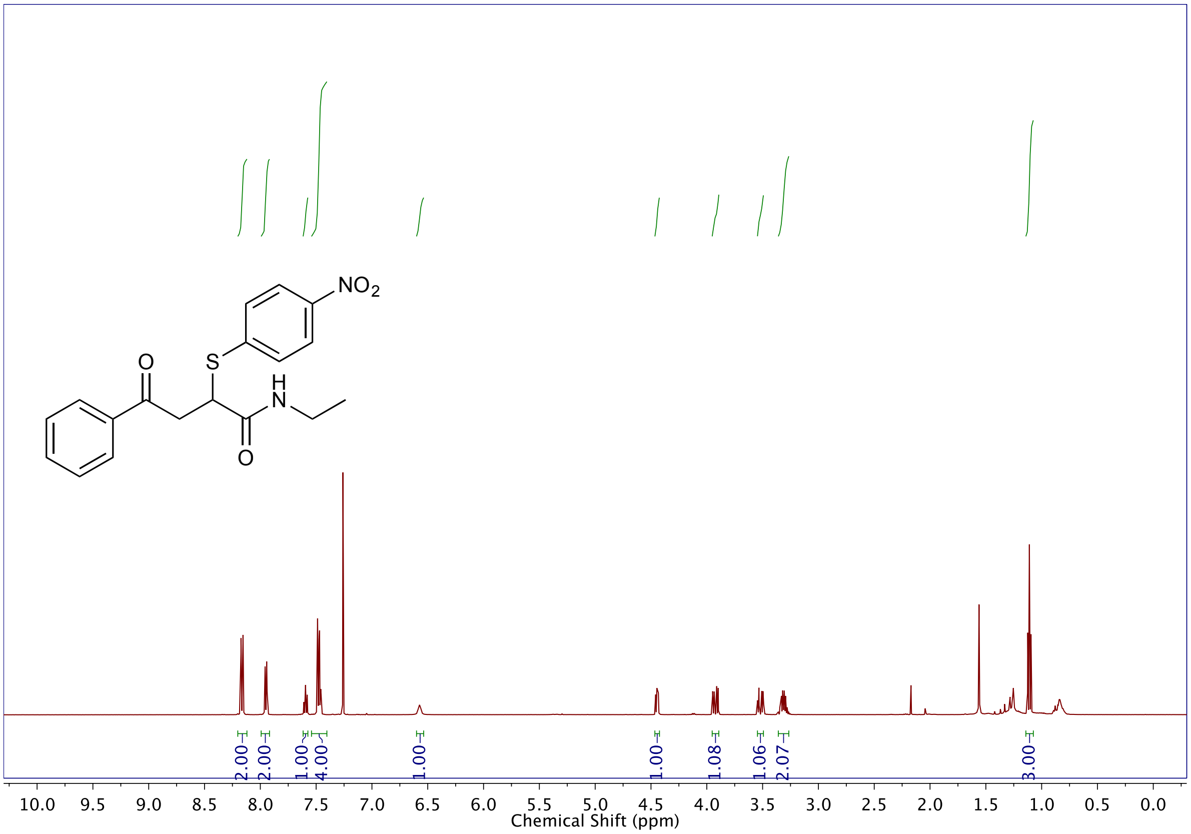

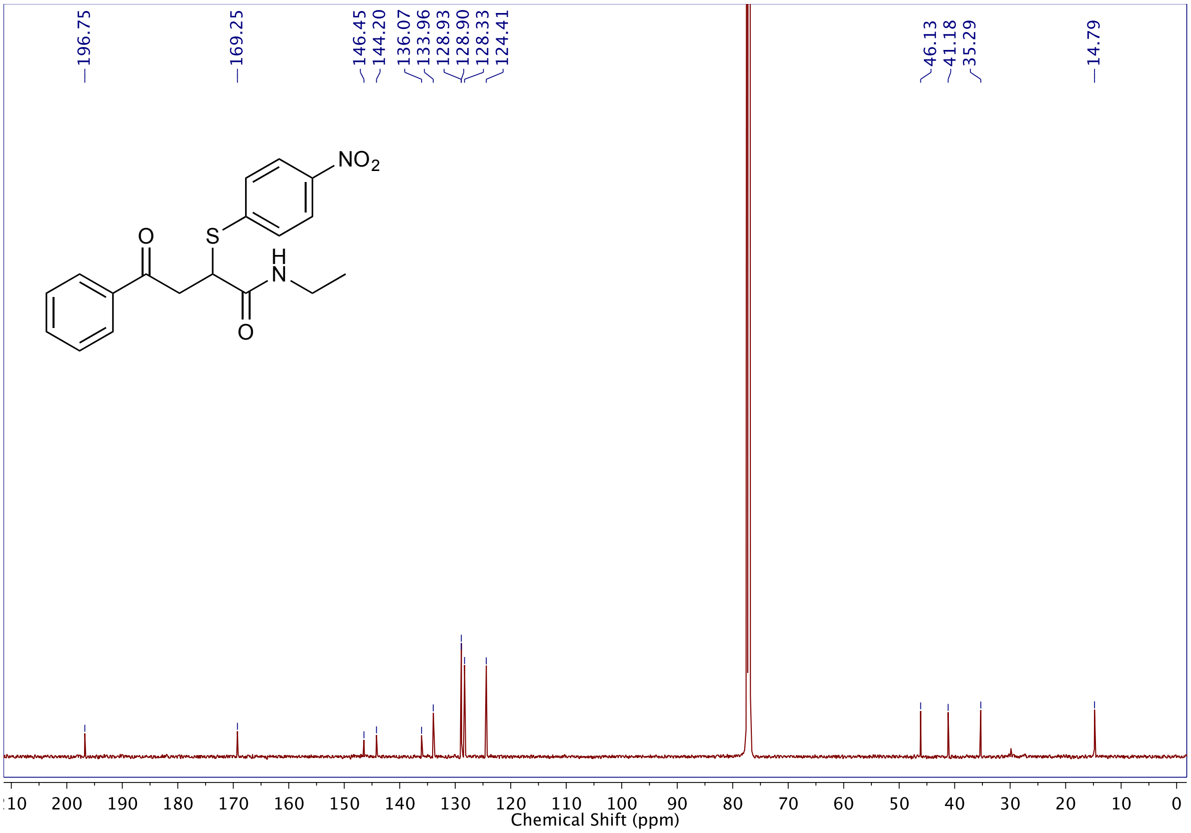

Supplementary Fig. 43 ^{1}H and ^{13}C spectra of compound **5**.

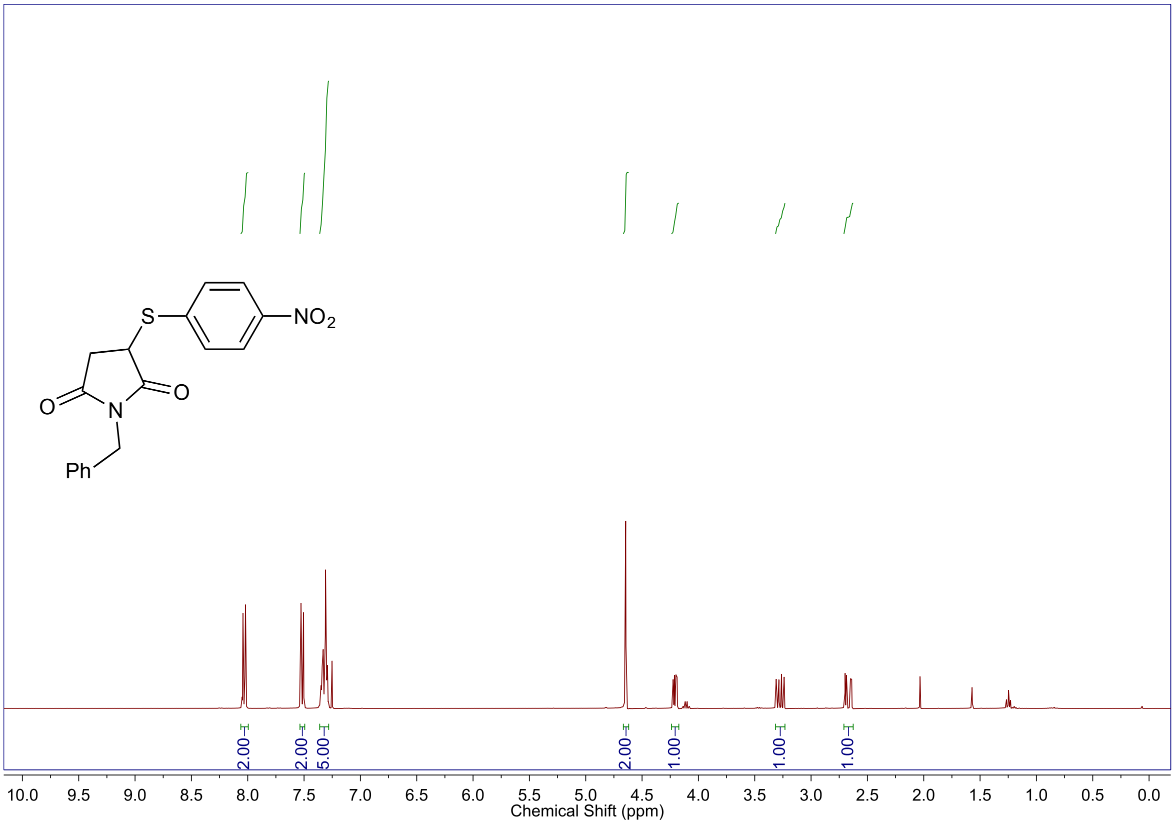

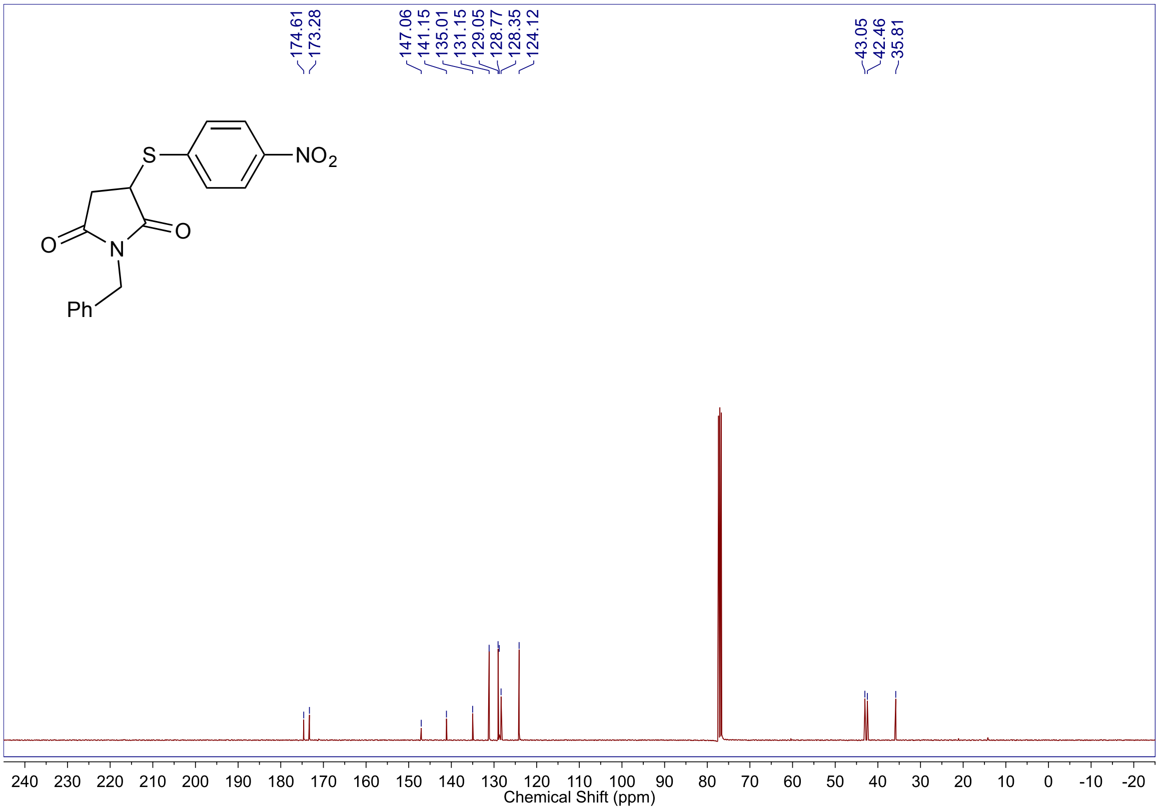

Supplementary Fig. 44 ^{1}H and ^{13}C spectra of compound **6**.

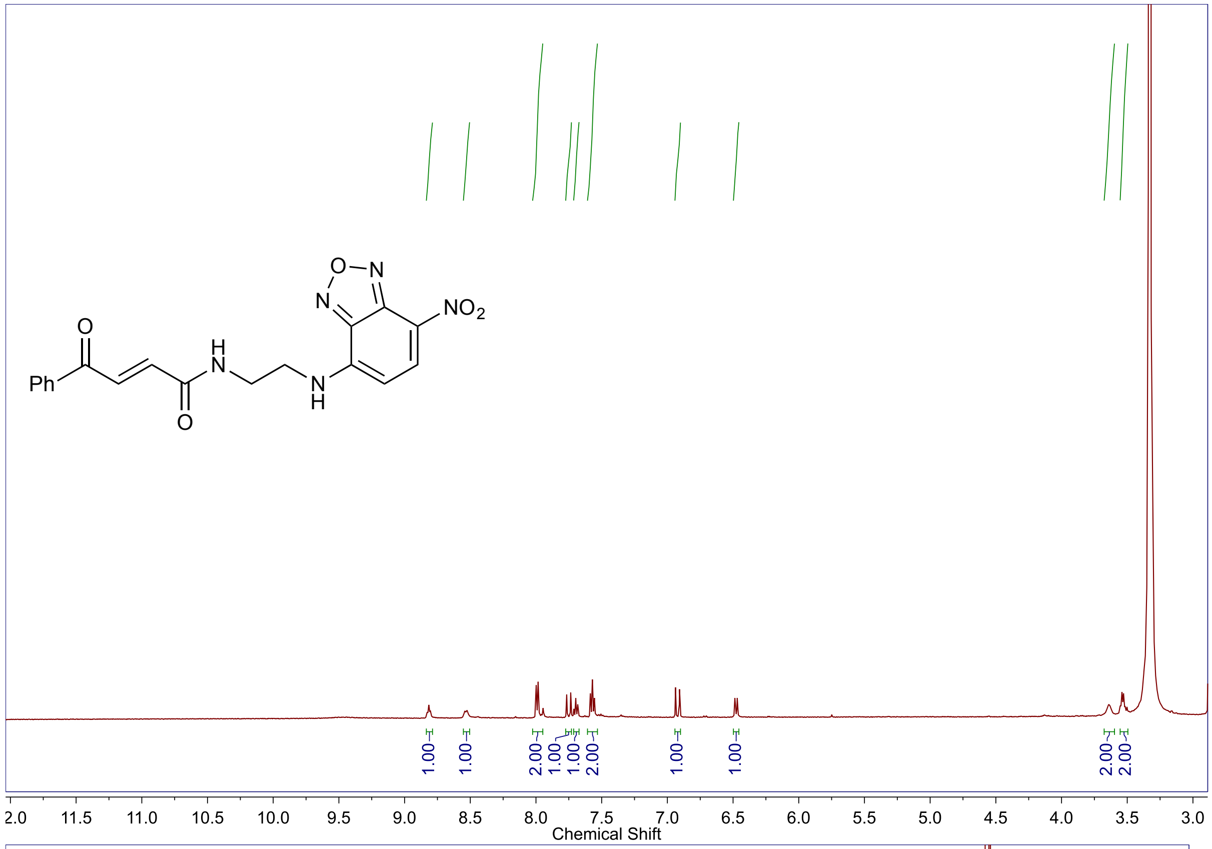

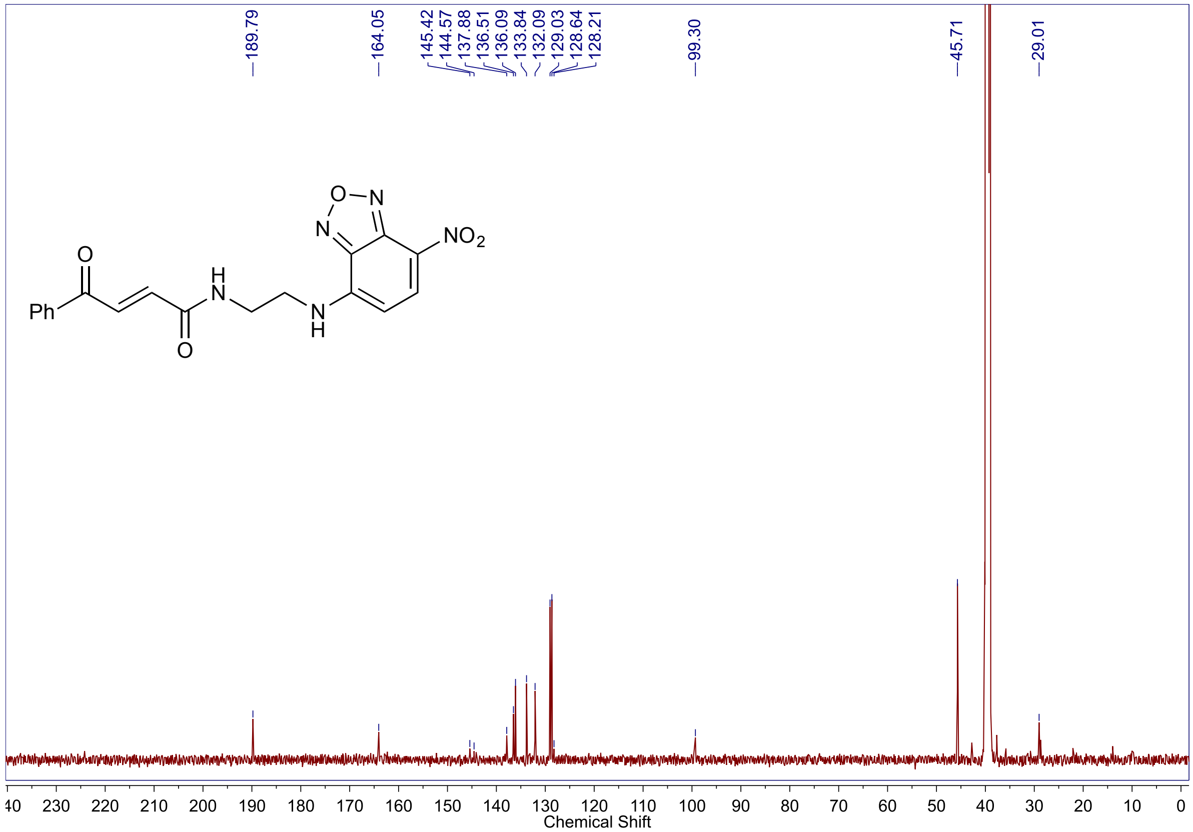

Supplementary Fig. 45 ^{1}H and ^{13}C spectra of compound **7**.

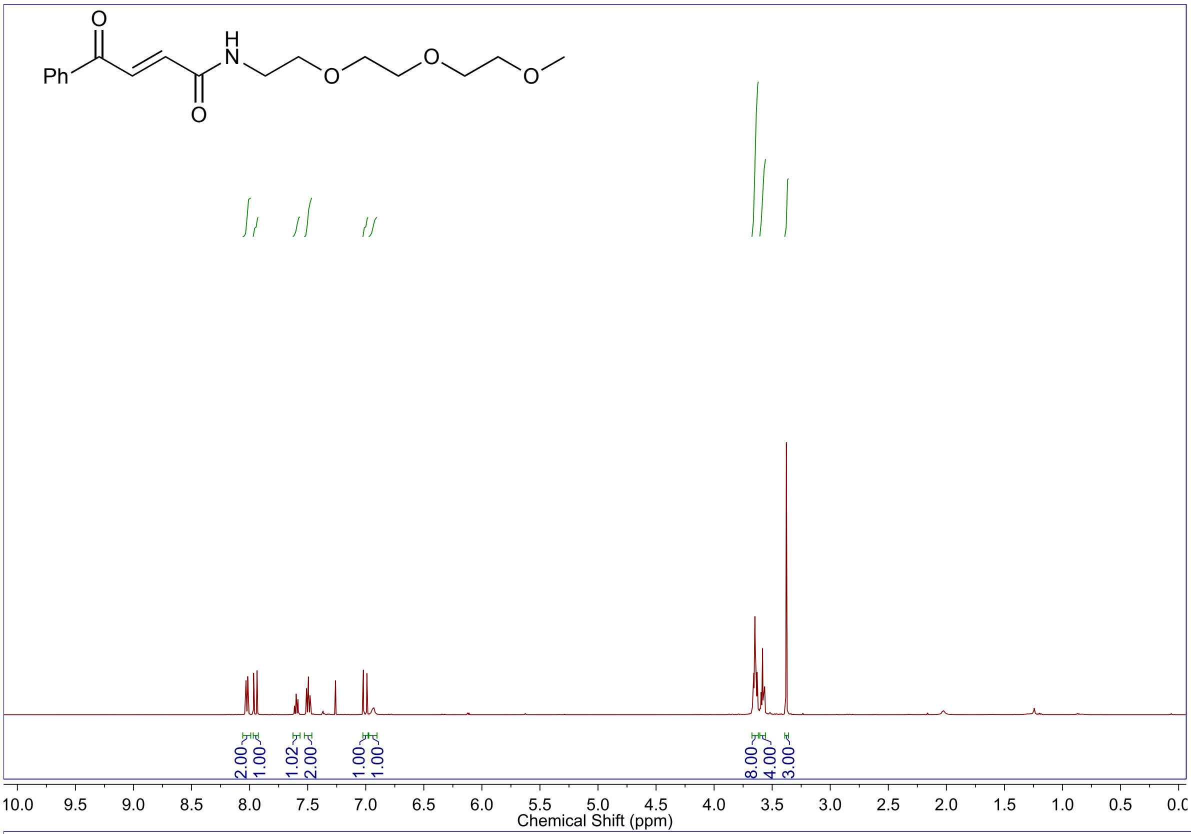

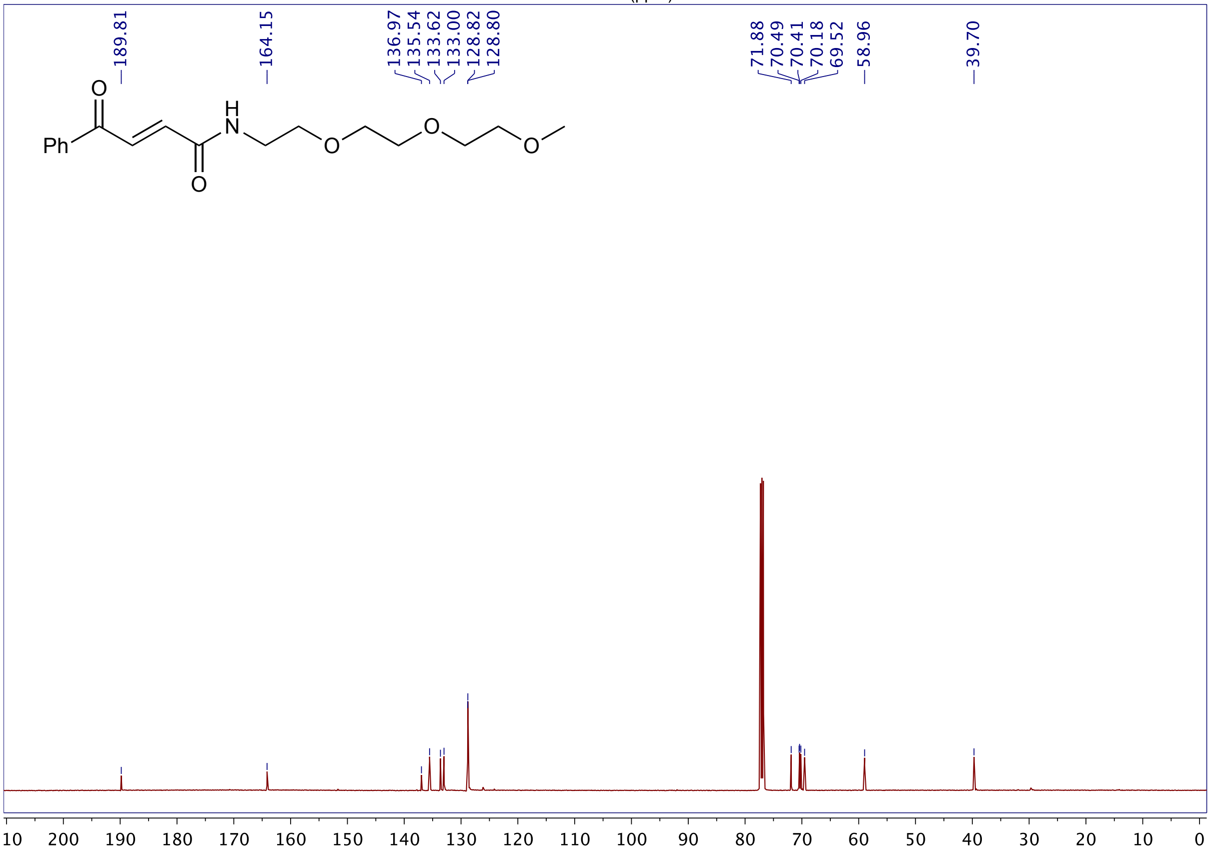

Supplementary Fig. 46 ^{1}H and ^{13}C spectra of compound **8**.

Supplementary Table

Supplementary Table 1 Energies, entropies, and lowest frequencies of the lowest energy calculated structures.[a]

Structure	E_{elec} (Hartree)	E_{elec} + ZPE (Hartree)	H (Hartree)	S (cal mol^{-1} K^{-1})	G (Hartree)	Lowest freq. (cm^{-1})	# of imag freq.
**SMe**	–438.170102	–438.133138	–438.129146	55.9	–438.155704	715.7	0
**2a**	–689.921193	–689.704406	–689.689190	124.3	–689.744753	18.1	0
**2a_ts1**	–1128.091365	–1127.837276	–1127.818703	140.0	–1127.881430	–198.3	1
**2a_ts2**	–1128.093158	–1127.838752	–1127.820004	142.2	–1127.883221	–145.7	1
**2a_add1**	–1128.099366	–1127.844285	–1127.825531	141.0	–1127.888551	24.1	0
**2a_add2**	–1128.107322	–1127.851674	–1127.832928	142.7	–1127.895792	16.7	0
**2b**	–498.250002	–498.087303	–498.075024	106.8	–498.124167	32.4	0
**2b_ts1**	–936.419358	–936.219327	–936.203575	124.9	–936.260796	–182.5	1
**2b_ts2**	–936.418891	–936.218965	–936.202903	128.9	–936.260603	–153.5	1
**2b_add1**	–936.424737	–936.224261	–936.208139	128.7	–936.265570	20.0	0
**2b_add2**	–936.431932	–936.230198	–936.214487	125.3	–936.271072	20.7	0
**2c**	–670.066070	–669.836809	–669.821279	125.7	–669.877250	20.0	0
**2c_ts1**	–1108.227319	–1107.960783	–1107.941959	140.7	–1108.005321	–240.3	1
**2c_ts2**	–1108.235140	–1107.968112	–1107.949171	142.7	–1108.012519	–167.0	1
**2c_add1**	–1108.233825	–1107.966019	–1107.947134	141.2	–1108.010285	22.9	0
**2c_add2**	–1108.252214	–1107.984125	–1107.965099	143.9	–1108.028293	21.3	0
**2d**	–573.049126	–572.827511	–572.812215	126.1	–572.867153	10.8	0
**2d_ts1**	–1011.205160	–1010.946393	–1010.927636	141.9	–1010.990759	–243.0	1
**2d_ts2**	–1011.205160	–1010.946395	–1010.927636	141.9	–1010.990761	–243.0	1
**2d_add1**	–1011.211783	–1010.951652	–1010.932967	141.1	–1010.995617	25.2	0
**2d_add2**	–1011.211721	–1010.951579	–1010.932929	140.4	–1010.995520	26.1	0

[a]Energy values calculated at the PCM_{water}/M06-2X/6-31+G(d,p) level. 1 Hartree = 627.51 kcal mol^{-1}. Thermal corrections at 298.15 K.

Supplementary Methods

DFT calculations.

Full geometry optimizations were carried out with Gaussian 09[1] using the M06-2X hybrid functional[2] and 6-31+G(d,p) basis set. Bulk solvent effects in water were considered implicitly through the IEF-polarizable continuum model.[3] The possibility of different conformations was taken into account for all structures. Frequency analyses were carried out at the same level used in the geometry optimizations, and the nature of the stationary points was determined in each case according to the appropriate number of negative eigenvalues of the Hessian matrix. Scaled frequencies were not considered. The quasiharmonic approximation reported by Trular *et al.* was used to replace the harmonic oscillator approximation for the calculation of the vibrational contribution to enthalpy and entropy.[4] Scaled frequencies were not considered. Mass-weighted intrinsic reaction coordinate (IRC) calculations were carried out by using the Gonzalez and Schlegel scheme[5,6] in order to ensure that the transition states (TSs) indeed connected the appropriate reactants and products. Gibbs free energies (ΔG) were used for the discussion on the relative stabilities of the considered structures. Cartesian coordinates, electronic energies, entropies, enthalpies, Gibbs free energies, and lowest frequencies of the calculated structures are available below (Supplementary Table 1 and Supplementary Fig. 1 and Supplementary Data 1).

General remarks.

All solvents were commercially available grade. All reactions were carried out under argon atmosphere unless otherwise mentioned. All reagents were purchased from either Sigma-Aldrich or Alfa-Aesar and used without further purification. Reaction mixtures were analyzed by analytical thin-layer chromatography and flash column chromatography was performed on Merck TLC Silica gel 60 F254 glass plates and Silica Gel high purity grade (Merck grade 9385 pore size 60Å, 230-400 mesh particle size), respectively. Visualization was accomplished with UV light (254 nm), ninhydrin or $KMnO_4$. ^1H NMR and ^{13}C NMR spectra were recorded on a Bruker 400 MHz DPX-400 Dual Spectrometer and Bruker 500 MHz AVIII HD Smart Probe in the stated solvents (DMSOd_6, $CDCl_3$, CD_3CN or D_2O) using tetramethylsilane as an internal standard. Chemical shifts were reported in parts per million (ppm) on the δ scale from an internal standard (NMR multiplicity abbreviations: s, singlet; d, doublet; t, triplet; q, quartet; m, multiplet; br, broad). Coupling constants, *J*, are reported in Hertz. FT-IR analysis were recorded using a Pelkin-Elmer spectrum one FT-IR universal with ATR sampling accessories. Mass spectroscopy was performed using a Waters micromass ZQ (LCMS) with Waters 2795 HPLC and a Waters 2996 photodiode array detector. This system is an automated service utilizing electrospray (ESI) ionization. The mobile phases are 95% aqueous acetonitrile with 0.05% formic acid and 10 mM ammonium acetate with 0.1% formic acid. The separation technology is based on a 50x4.6 mm C18 column (currently a Phenomenex Kinetix solid core column). There are several methods available enabling the user to produce mass spectra for compounds up to 2 kDa in positive and negative modes of ionization. In some cases, a Waters LCT Premier combined with an Agilent 1100 autosampler was also used. The system runs using 50% aqueous acetonitrile with 0.25% formic acid as mobile phase and can measure accurate masses from 150 Da to 1500 Da.

Synthesis of carbonylacrylic Michael-acceptors.

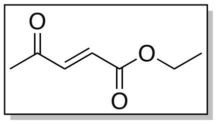

(*E*)-ethyl 4-oxopent-2-enoate 2b was prepared following a reported procedure.[7] To a solution of 1-(triphenylphosphoranylidene)-2-propanone (2.10 g, 6.59 mmol, 1.0 equiv.) and ethyl glycolate (0.75 mL, 7.92 mmol, 1.2 equiv.) in anhydrous CH_2Cl_2 (22 mL) was added MnO_2 (5.75 g, 65.9 mmol, 10.0 equiv.) and vigorously stirred at ambient temperature (25 ºC) for 30 h. The mixture was filtered through a short pad of Celite and the filtrate was concentrated using rotary evaporation. The residue was then diluted with cold Et_2O (10 mL), filtered through a plug of Celite and washed with additional Et_2O (5 mL). The filtrate was concentrated by rotary evaporation and purified by flash chromatography (5 to 20% EtOAc in hexanes) providing **2b** (618 mg, 4.35 mmol, 66% yield) as a pale yellow liquid: R_f = 0.40 (1:9, EtOAc/hexanes); ^{1}H NMR (400 MHz, Chloroform-*d*) δ 6.98 (d, *J* = 16.2 Hz, 1H), 6.61 (d, *J* = 16.1 Hz, 1H), 4.23 (q, *J* = 7.1 Hz, 2H), 2.32 (s, 3H), 1.29 (t, *J* = 7.1 Hz, 3H); ^{13}C NMR (100 MHz, Chloroform-*d*) δ 197.8, 165.6, 140.1, 131.8, 61.6, 28.3, 14.3; HRMS ESI$^+$ (*m/z*): Calcd. For $C_7H_{11}O_3^+$ [M + H]$^+$ 143.0703, found 143.0698; FTIR (cm^{-1}): 2986, 1721, 1701, 1683, 1641, 1467, 1424, 1365, 1290, 1223, 1181, 1155, 1096, 1029, 978, 947, 869.

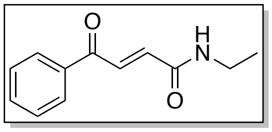

(*E*)-*N*-ethyl-4-oxo-4-phenylbut-2-enamide 2c was prepared by a modified reported procedure.[8] 1.12 g (6.4 mmol) of commercially available 3-benzoylacrylic acid were dissolved in dimethoxyethane (13 mL) and cooled to -10 ºC under argon. Isobutylchloroformate (1.18 mL, 7.7 mmol) was added followed by 0.85 mL (7.7 mmol) of *N*-methylmorpholine. Afterwards, 8.0 ml (16.0 mmol) of a 2M solution of ethylamine in THF was added and stirred for 15 min at -10 ºC and 1 h at RT. 10 mL H_2O was added and the mixture was extracted with CH_2Cl_2 (3 x 15 mL). Organic layers were combined, washed with 10 mL sat. aqueous NaOH solution and H_2O, dried over anhydrous Na_2SO_4 and evaporated. The residue was purified by flash chromatography (10 to 80% EtOAc in hexanes) to yield pure **2c** (1.04 g, 5.10 mmol, 80% yield) as a pale yellow solid: R_f = 0.47 (4:1, EtOAc/hexanes); ^{1}H NMR (400 MHz, Chloroform-*d*) δ 8.05 – 8.01 (m, 2H), 7.97 (d, *J* = 15.0 Hz, 1H), 7.64 – 7.57 (m, 1H),

7.54 – 7.47 (m, 2H), 6.95 (d, *J* = 15.0 Hz, 1H), 5.97 (s, 1H), 3.45 (qd, *J* = 7.3, 5.7 Hz, 2H), 1.23 (t, *J* = 7.3 Hz, 3H); ^{13}C NMR (100 MHz, CD_3CN) δ 191.1, 164.5, 138.0, 136.9, 134.5, 133.1, 129.8, 129.6, 35.2, 14.7; HRMS ESI$^+$ (*m/z*): Calcd. For $C_{12}H_{14}NO_2^+$ [M + H]$^+$ 204,1019, found 204.0995; FTIR (cm^{-1}): 3258, 1637, 1593, 1559, 1447, 1357, 1324, 1289, 1196, 1009, 977.

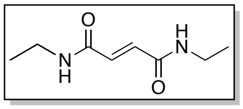

***N*[1],*N*[4]-diethylfumaramide 2d** Fumaric acid (1 g, 8.61 mmol), *N*-*N*-diisopropyl ethyl amine (3.3 mL, 19.0 mmol, 2.2 equiv.) and TBTU (5.5 g, 17.0 mmol, 2 equiv.) were dissolved in DMF (17 mL). A 2 M solution of ethylamine in THF (9.5 mL, 19.0 mmol, 2.2 equiv.) was then added and the reaction mixture was stirred at room temperature overnight. The reaction mixture was diluted with water and thrice extracted with dichloromethane. The combined organic phases were dried over sodium sulfate and evaporated. The crude product was purified by column chromatography (1 to 10% MeOH in CH_2Cl_2). The title compound **2d** (885 mg, 5,2 mmol, 60% yield) was obtained as a white solid. R_f = 0.29 (2:98, MeOH/CH_2Cl_2); ^{1}H NMR (400 MHz, DMSO-*d*$_6$) δ 6.74 (s, 2H), 3.14 (q, *J* = 7.3 Hz, 4H), 1.02 (t, *J* = 7.3 Hz, 6H); ^{13}C NMR (100 MHz, DMSO-*d*$_6$) δ 164.6, 133.1, 34.4, 14.9; HRMS ESI$^+$ (*m/z*): Calcd. For $C_8H_{15}N_2O_2^+$ [M + H]$^+$ 171.1128, found 171.1205; FTIR (cm^{-1}): 3282.00, 3068.18, 2977.87, 2936.84, 2887.13, 1622.83, 1480.55, 1405.59, 1367.81, 1317.23, 1198.65, 1144.83, 1103.32, 1043.17, 1013.02, 992.01, 981.76, 923.03, 910.08.

General procedure for thiol-Michael addition on small molecules.

Treating carbonylacrylic derivatives with *N*-(tert-Butoxycarbonyl)-*L*-cysteine methyl ester **1** in a solution of MeCN/sodium phosphate buffer (pH 8.0, 50 mM) at room temperature for 2 min gave excellent isolated yield of the desired cysteine-conjugates:

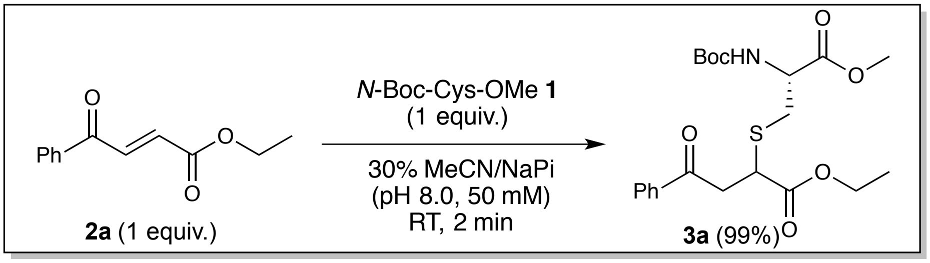

To a solution of *N*-(tert-Butoxycarbonyl)-*L*-cysteine methyl ester **1** (23.2 mg, 0.098 mmol, 1 equiv.) in 2 mL of Sodium phosphate buffer (pH 8.0, 50 mM) containing 30% of MeCN was added a solution of commercially available ethyl (*E*)-4-oxo-4-phenylbut-2-enoate **2a** (20.0 mg, 0.098 mmol, 1 equiv.) in MeCN (0.5 mL) at room temperature. The completion of reaction was monitored by TLC using short UV and ninhydrin staining solution. After 2 min, the reaction mixture was concentrated *in vacuo* to give the pure product **3a** in 99% yield (42.5 mg, 0.097 mmol) as a clear oil: R_f = 0.11 (1:1, EtOAc/hexanes); ^{1}H NMR (500 MHz, DMSO-d_6, 100 ºC) δ 7.97 (d, *J* = 7.3 Hz, 2H), 7.65 (t, *J* = 7.4 Hz, 1H), 7.53 (t, *J* = 7.7 Hz, 2H), 6.86 (s, 1H), 4.32 – 4.21 (m, 1H), 4.19 – 4.10 (m, 2H), 3.91 – 3.84 (m, 1H), 3.68 (d, *J* = 2.5 Hz, 3H), 3.67 – 3.59 (m, 1H), 3.46 – 3.29 (m, 1H), 3.19 – 3.08 (m, 1H), 3.06 – 2.98 (m, 1H), 1.41 (s, 9H), 1.22 (t, *J* = 7.1 Hz, 3H); ^{13}C NMR (125 MHz, DMSO-d_6, recorded at 100 ºC, major diastereomer) δ 196.4, 170.7, 170.5, 154.5, 135.8, 132.8, 128.1, 127.3, 78.2, 60.2, 60.2, 53.7, 53.2, 51.3, 41.6, 40.9, 40.6, 40.3, 32.5, 32.2, 27.6, 13.2; HRMS ESI$^+$ (*m/z*): Calcd. For $C_{21}H_{30}NO_7S^+$ [M + H]$^+$ 440.1737, found 440.1737.; FTIR (cm^{-1}): 2981, 1712, 1684, 1597, 1581, 1501, 1449, 1392, 1366, 1246, 1212, 1096, 1052, 1020, 1002, 988, 916, 857, 756.

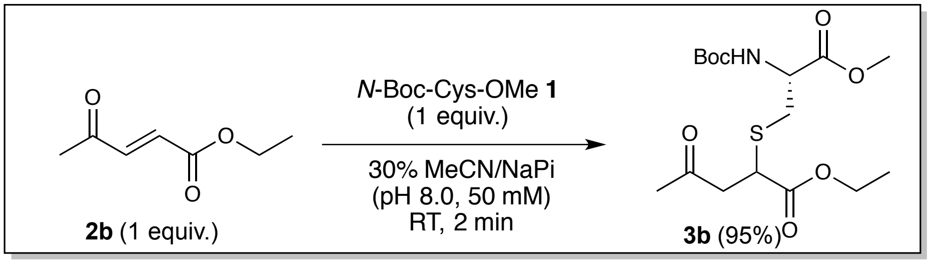

To a solution of *N*-(tert-Butoxycarbonyl)-*L*-cysteine methyl ester **1** (16.5 mg, 0.070 mmol, 1 equiv.) in 1.4 mL of 30% MeCN/sodium phosphate buffer (pH 8.0, 50 mM) was added a solution of ethyl (*E*)-4-oxopent-2-enoate **2b** (10.0 mg, 0.070 mmol, 1 equiv.) in MeCN (0.2 mL) at room temperature. The completion of reaction was monitored by TLC using short UV and ninhydrin staining solution. After 5 min, the reaction mixture was concentrated *in vacuo* to give the pure product **3b** in 95% yield: R_f = (25.3 mg, 0.067 mmol) as a clear oil: 0.40 (1:1, EtOAc /hexanes); ^{1}H NMR (500 MHz, 90°C, DMSO-d_6) δ 6.86 (s, 1H), 4.31 – 4.16 (m, 1H), 4.18 – 4.04 (m, 2H), 3.67 (s, 3H), 3.09 – 3.03 (m, 2H), 2.98 – 2.78 (m, 2H), 2.11 (s, 3H), 1.40 (s, 9H), 1.22 (t, *J* = 7.1 Hz, 3H); ^{13}C NMR (125 MHz, DMSO-d_6, recorded at 90 ºC, major diastereomer) δ 204.3, 204.3, 170.7, 170.7, 78.3, 60.3, 51.4, 44.6, 44.3, 41.1, 40.4, 32.4, 32.1, 28.9, 27.7, 13.3; HRMS ESI$^+$ (*m/z*): Calcd. For $C_{16}H_{28}NO_7S^+$ [M + H]$^+$ 378.1581, found 378.1585; FTIR (cm^{-1}): 2979, 1712, 1508, 1437, 1392, 1365, 1319, 1233, 1095, 1044, 1018, 860, 779.

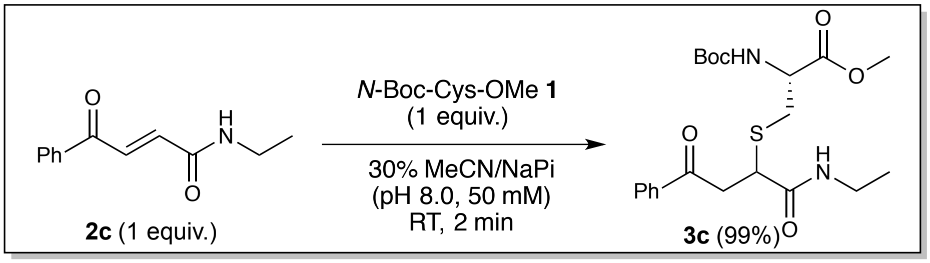

To a solution of *N*-(tert-Butoxycarbonyl)-*L*-cysteine methyl ester **1** (23.2 mg, 0.098 mmol, 1 equiv.) in 2 mL of 30% MeCN/sodium phosphate buffer (pH 8.0, 50 mM) was added a solution of (*E*)-*N*-ethyl-4-oxo-4-phenylbut-2-enamide **2c** (20.0 mg, 0.098 mmol, 1 equiv.) in MeCN (0.5 mL) at room temperature. The completion of reaction was monitored by TLC using short UV and ninhydrin staining solution. After 5 min, the reaction mixture was concentrated *in vacuo* to give the pure product **3c** in 99% yield (42.5 mg, 0.097 mmol) as a clear oil: R_f = 0.63 (4:1, EtOAc/hexanes); ^{1}H NMR (500

MHz, DMSO-*d6,* recorded at 100 ºC) δ 7.94 (d, *J* = 8.5 Hz, 2H), 7.73 (s, 1H), 7.63 (t, *J* = 7.5 Hz, 1H), 7.52 (t, *J* = 7.6 Hz, 2H), 6.77 (s, 1H), 4.27 – 4.18 (m, 1H), 3.88 – 3.79 (m, 1H), 3.72 – 3.67 (m, 1H), 3.66 (s, 3H), 3.27 (dt, *J* = 17.4, 4.9 Hz, 1H), 3.16 – 3.10 (m, 2H), 3.10 – 3.04 (m, 1H), 2.95 – 2.91 (m, 1H), 1.39 (s, 9H), 1.06 (t, *J* = 7.2 Hz, 3H); ^{13}C NMR (125 MHz, DMSO-*d6,* recorded at 100 ºC, major diastereomer) δ 196.7, 170.7, 169.4, 154.5, 136.3, 132.5, 128.0, 127.3, 78.2, 53.5, 51.2, 42.3, 40.8, 33.3, 31.8, 27.6, 13.8; HRMS ESI$^+$ (*m/z*): Calcd. For $C_{21}H_{31}N_2O_6S^+$ [M + H]$^+$ 439.1897, found 439.1899; FTIR (cm^{-1}): 2978, 1683, 1597, 1519, 1449, 1413, 1392, 1366, 1351, 1248, 1217, 1052, 1019, 988, 912, 858.

Selectivity studies.

Selectivity of carbonylacrylic derivative for cysteine over lysine. Treatment of **2a** with an equimolar mixture of *N*-Boc-Cysteine-OMe and *N*-Boc-Lysine-OMe, *N*-Boc-Cysteine-OMe **1** and *N*-Boc-Lysine-OMe **4**.

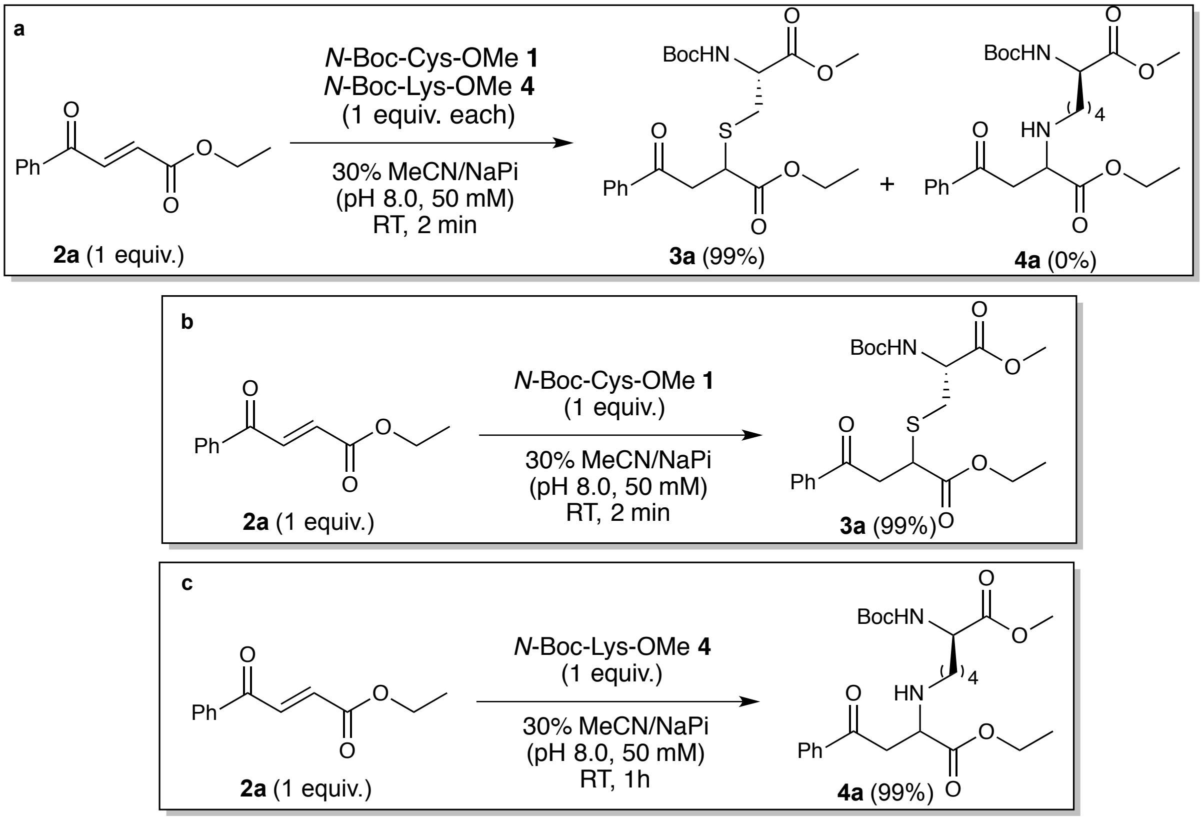

Treating compound **2a** with *N*-Boc-Cysteine-OMe **1** in phosphate buffer (pH 8, 50 mM) at room temperature for 2 min gave excellent isolated yield (99%) of the desired cysteine-conjugate. In contrast, treatment of **2a** with *N*-Boc-Lysine-OMe **4** under the same conditions required 1 h for completion of the reaction (followed by TLC). An equimolar mixture of *N*-Boc-Cysteine-OMe **1** (57.6 mg, 0.245 mmol, 1 equiv.) and *N*-Boc-Lysine-OMe **4** (63.7 mg, 0.245 mmol, equiv.) in MeCN (0.05 M) or in a 30% MeCN/Na-Pi buffer (50 mM) at pH 8 (0.05 M) was treated with **2a** (50.0 mg, 0.245 mmol, 1 equiv.) and gave only the cysteine-conjugate **3a** in 99% isolated whereas no *N*-lysine adduct was obtained.

Reaction kinetics between *N*-Boc-Cys-OMe 1 and 2a and 2c.

The second order reaction constant of the reaction between cysteine derivative **1** and two carbonylacrylic derivatives was determined at 20 ºC under second order conditions in 30% acetonitrile in sodium phosphate buffer (pH 8.0, 50 mM) by UV spectroscopy using a Cary 400 Scan UV-Visible Spectrophotometer. Concentration of compound **1** and carbonylacrylic derivatives was identical (0.075 mM). The absorption at 269 nm (for **2a**) or at 275 nm (for **2c**) was measured every 0.1 min. The second order rate constant k_2 was obtained from the slope of a plot of $(1/c - 1/c_0)$ *versus* time (Supplementary Fig. 3).

Glutathione exchange studies.

Reactions were performed by treating GSH - reduced glutathione (21 mg, 0.068 mmol, 1.5 equiv.) with compound **3c** (20 mg, 0.046 mmol, 1 equiv.) in 2 mL deuterated solution of NaP_i buffer (50 mM) at pH 7.4 containing 40% of CD_3CN at room temperature. After 100 and 200 h, a 0.5 mL aliquot was collected and analyzed by ^1H NMR and mass spectrometry. No exchange glutathione product was found (HRMS ESI$^+$ (*m/z*): Calcd. For $C_{22}H_{31}N_4O_8S^+$ $[M + H]^+$ 511.1857 or $C_{22}H_{30}N_4O_8SNa^+$ $[M + Na]^+$ 533.1677). See Supplementary Figs 5 and 6.

Kinetic assays for reverse Michael reactions on chromogenic thiol adducts.

Synthesis and characterization of 4-nitrothiophenyl conjugates 5 and 6

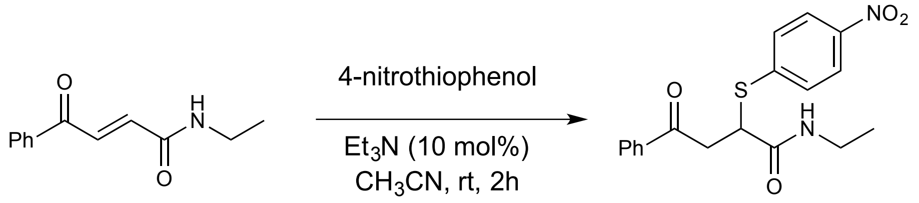

5 (95%)

***N*-ethyl-2-((4-nitrophenyl)thio)-4-oxo-4-phenylbutanamide 5** A solution of (*E*)-*N*-ethyl-4-oxo-4-phenylbut-2-enamide **2c** (100.0 mg, 0.45 mmol, 1 equiv.), 4-nitrothiophenol (108.0 mg, 0.45 mmol, 1 equiv.) and catalytic Et_3N (7 μL, 0.049 mmol) in anhydrous MeCN (10 mL) was stirred at room temperature under nitrogen atmosphere. After 2 h, the reaction mixture was concentrated and the crude was purified by column chromatography (3:7, EtOAc/hexanes) to give compound **5** (112 mg, 70%) as white solid: R_f = 0.43 in 3:7, EtOAc/hexanes); ^{1}H NMR (500 MHz, Chloroform-*d*) δ 8.20 – 8.13 (m, 2H), 7.97 – 7.91 (m, 2H), 7.62 – 7.56 (m, 1H), 7.51 – 7.43 (m, 4H), 6.57 (s, 1H), 4.45 (dd, *J* = 6.7, 5.5 Hz, 1H), 3.92 (dd, *J* = 18.0, 6.7 Hz, 1H), 3.52 (dd, *J* = 18.1, 5.5 Hz, 1H), 3.37 – 3.24 (m, 2H), 1.11 (t, *J* = 7.3 Hz, 3H); ^{13}C NMR (125 MHz, Chloroform-*d*) δ 196.8, 169.3, 146.5, 144.2, 136.1, 134.0, 128.9, 128.9, 128.3, 124.4, 46.1, 41.2, 35.3, 14.8; HRMS ESI$^+$ (*m/z*): Calcd. For $C_{18}H_{19}N_2O_4S^+$ $[M + H]^+$ 359.1060, found 359.1072; FTIR: 3271, 3097, 2985, 2903, 1682, 1637, 1595, 1577, 1517, 1481, 1446, 1401, 1366, 1341, 1279, 1264, 1223, 1189, 1165, 1148, 1114, 1082, 1010, 989.76.

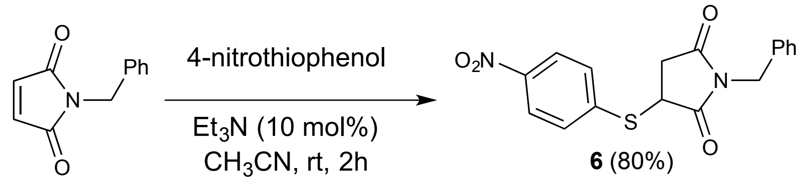

6 (80%)

1-benzyl-3-((4-nitrophenyl)thio)pyrrolidine-2,5-dione 6 was prepared following a described procedure[9]. A solution of *N*-benzylmaleimide (150.0 mg, 0.80 mmol, 1 equiv.), 4-nitrothiophenol (124.0 mg, 0.80 mmol, 1 equiv.) and catalytic Et_3N (11 μL, 0.08 mmol) in anhydrous MeCN (10 mL) was stirred at room temperature under nitrogen atmosphere. After 2 h, the reaction mixture was concentrated and the crude

was purified by column chromatography (3:7, EtOAc/hexanes) to give compound **6** (178 mg, 0.52 mmol, 65%) as colourless oil (all data is in accordance with that described in the literature): R_f = 0.37 in 3:7, EtOAc/hexanes; ^{1}H NMR (400 MHz, Chloroform-*d*) δ 8.08 – 7.97 (m, 2H), 7.58 – 7.47 (m, 2H), 7.37 – 7.25 (m, 5H), 4.65 (s, 2H), 4.21 (dd, *J* = 9.4, 4.3 Hz, 1H), 3.27 (dd, *J* = 18.8, 9.4 Hz, 1H), 2.67 (dd, *J* = 18.8, 4.4 Hz, 1H); ^{13}C NMR (101 MHz, Chloroform-*d*) δ 174.6, 173.3, 147.1, 141.2, 135.0, 131.1, 129.1, 128.8, 128.3, 124.1, 43.0, 42.5, 35.8.

Procedure for kinetic studies on compounds 5 and 6.

To 165 μL sodium phosphate buffer (pH 7.4, 10 mM) in a quartz cuvette was added a stock solution of compound **5** (33 μL of 0.45 mM solution in MeCN) and the mixture was thoroughly mixed by pipetting up and down. Absorbance at 410 nm corresponding to the release of the 4-nitrothiophenolate anion was measured at room temperature over 5 h. The same procedure was followed for compound **6**. See Supplementary Fig. 7.

Synthesis of fluorescent and PEG derivatives.

Synthesis of fluorescent derivative 7

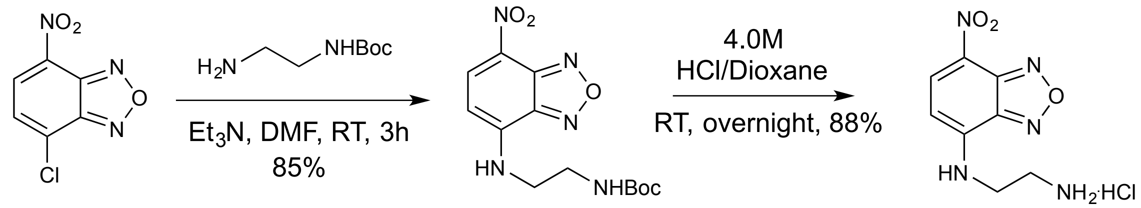

***tert*-butyl (2-((7-nitrobenzo[*c*][1,2,5]oxadiazol-4-yl)amino)ethyl)carbamate** was prepared following a reported procedure[10]. Under argon atmosphere, 4-chloro-7-nitrobenzofurazan- NBD-Cl (199 mg, 1.0 mmol, 1 equiv.) was dissolved in anhydrous DMF (6 mL). After adding triethylamine (138 μL, 1.0 mmol, 1 equiv.) and *N*-Boc-1,2-diaminoethane (174 μL, 1.2 mmol, 1.2 equiv.), the mixture was stirred at room temperature for 3 h. After completion of the reaction followed by TLC (1:1, EtOAc/hexanes, using UV and $KMnO_4$ staining solution), the reaction solution was poured into a saturated aqueous ammonium chloride solution (30 mL). The mixture was extracted with ethyl acetate (3 x 40 mL), and the organic layer was washed with water (50 mL) and brine (50 mL) and then dried over magnesium sulphate. After solvent removal under reduced pressure, brown oily *N*-Boc protected intermediate (275 mg, 0.85 mmol) was obtained with spectral data in accordance with the ones reported in the literature[10]: ^{1}H NMR (400 MHz, Chloroform-*d*) δ 8.48 (d, *J* = 8.7 Hz, 1H), 8.01 (br s, 1H), 6.16 (d, *J* = 8.6 Hz, 1H), 5.09 (br s, 1H), 3.59 (br s, 4H), 1.46 (s, 9H); HRMS ESI$^+$ (*m/z*): Calcd. For $C_{13}H_{18}N_5O_5^+$ $[M + H]^+$ 324.1302, found 324.1302.

***N*^{1}-(7-nitrobenzo[*c*][1,2,5]oxadiazol-4-yl)ethane-1,2-diamine** Under argon atmosphere, the Boc-protected intermediate (262 mg, 0.81 mmol, 1 equiv.) was dissolved in a 4.0 M solution of HCl in dioxane (6mL). The solution was shaded from the light and stirred overnight. After confirming completion of the reaction by TLC (9:1, CH_2Cl_2/MeOH, using UV and $KMnO_4$ staining solution), the reaction solution was evaporated under reduced pressure and the brown solid (185.4 mg, 0.71 mmol) used in the next step. The spectral data in accordance with the one reported in the literature.[10] ^{1}H NMR (400 MHz, Methanol-d_4) δ 8.54 (d, *J* = 8.7 Hz, 1H), 6.47 (d, *J* = 8.7 Hz, 1H),

3.89 (t, J = 6.2 Hz, 2H), 3.34 (t, J = 6.3 Hz, 2H); HRMS ESI$^+$ (m/z): Calcd. $C_8H_{10}N_5O_3^+$ [M + H]$^+$ 224.0778, found 224.0784.

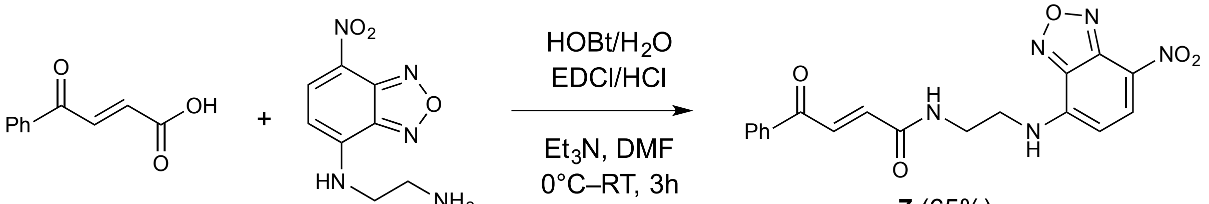

7 (65%)

(*E*)-*N*-(2-((7-nitrobenzo[*c*][1,2,5]oxadiazol-4-yl)amino)ethyl)-4-oxo-4-phenylbut-2-enamide 7 Under argon atmosphere, 3-benzoylacrylic acid (108.0 mg, 0.61 mmol, 1 equiv.) was dissolved in anhydrous DMF and triethylamine (170 μL, 1.22 mmol, 2.0 equiv.) was added dropwise. To the solution were sequentially added 1-hydroxybenzotriazole hydrate (111.5 mg, 0.73 mmol, 1.2 equiv.), *N*-(3-Dimethylaminopropyl)-*N′*-ethylcarbodiimide hydrochloride (140.0 mg, 0.73 mmol, 1.2 equiv.) and N^1-(7-nitrobenzo[*c*][1,2,5]oxadiazol-4-yl)ethane-1,2-diamine (149.5 mg, 0.61 mmol, 1 equiv.) under ice-cooling. Then, the reaction was shadded from light, and after reaching room temperature, stirred for 3 h. After detecting full conversion of the reactants by TLC (95:5, CH_2Cl_2/MeOH, using UV and $KMnO_4$ staining solution), the reaction was poured into an aqueous HCl solution (0.5 M, 40 mL) and extracted with CH_2Cl_2 (3 x 40 mL). The combined organic phase was washed with saturated Na_2CO_3 (2 x 40 mL) and brine (2 x 40 mL), then dried over magnesium sulphate. Removal of all volatiles *in vacuo* gave compound **7** as a yellow/brown solid (151.0 mg, 0.40 mmol, 65%). The product was obtained in excellent purity (^{1}H NMR analysis), the remainder of the material being composed of a mixture of unidentified minor components. Attempts to purify the compound by chromatography (silica, triethylamine washed silica, alumina, florisil) or recrystallization led to further decomposition. The material at ca. 95% purity was sufficient for subsequent experiments: R_f = 0.71 (95:5, CH_2Cl_2/MeOH); ^{1}H NMR (500 MHz, DMSO-d_6) δ 8.80 (t, J = 5.8 Hz, 1H), 8.52 (d, J = 8.8 Hz, 1H), 7.98 (d, J = 6.9 Hz, 2H), 7.74 (d, J = 15.3 Hz, 1H), 7.69 (t, J = 7.4 Hz, 1H), 7.56 (t, J = 7.8 Hz, 2H), 6.91 (d, J = 15.3 Hz, 1H), 6.47 (d, J = 9.0 Hz, 1H), 3.63 (bs, 2H), 3.56 – 3.47 (m, 2H); ^{13}C NMR (125 MHz, DMSO-d_6) δ 189.8, 164.1, 145.4, 144.6, 137.9, 136.5, 136.1, 133.8,

132.1, 129.0, 128.6, 128.2, 99.3, 45.7, 29.0; HRMS ESI$^+$ (*m/z*): Calcd. $C_{18}H_{16}N_5O_5^+$ [M + H]$^+$ 382,1146, found 382.1133.

Synthesis of PEG derivative 8

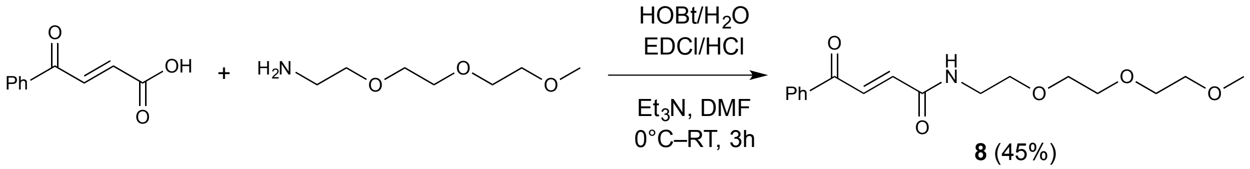

(*E*)-*N*-(2-(2-(2-methoxyethoxy)ethoxy)ethyl)-4-oxo-4-phenylbut-2-enamide 8 Under argon atmosphere, 3-benzoylacrylic acid (50.0 mg, 0.28 mmol, 1 equiv.) was dissolved in anhydrous DMF and triethylamine (78 μL, 0.56 mmol, 2.0 equiv.) was added. To the solution were sequentially added 1-hydroxybenzotriazole hydrate (46.0 mg, 0.34 mmol, 1.2 equiv.), *N*-(3-Dimethylaminopropyl)-*N′*-ethylcarbodiimide hydrochloride (65.0 mg, 0.34 mmol, 1.2 equiv.) and a 0.3 M solution (dry DMF) of 2-(2-(2-Methoxyethoxy)ethoxy) ethylamine (45.0 mg, 0.28 mmol, 1 equiv.) under ice-cooling. Then, the reaction was shadded from light, and after reaching room temperature, stirred for 3 h. After confirming completion of the reaction by TLC (95:5, CH_2Cl_2/MeOH, using UV and $KMnO_4$ staining solution), the reaction was poured into a 0.5 M aqueous hydrochloric acid solution (20 mL). The mixture was extracted with CH_2Cl_2 (3x 30 mL), and the organic phase washed with Na_2CO_3 (2 x 30 mL) and brine (2 x 30 mL). After drying over magnesium sulphate and purification by column chromatography (95:5, CH_2Cl_2/MeOH) a brown oil of **8** (43% yield, 38.0 mg, 0.12 mmol) was obtained after solvent removal under reduced pressure: R_f = 0.40 (5% MeOH:CH_2Cl_2); ^{1}H NMR (500 MHz, Chloroform-*d*) δ 8.04 – 8.00 (m, 2H), 7.95 (d, *J* = 15.1 Hz, 1H), 7.62 – 7.57 (m, 1H), 7.49 (dd, *J* = 8.3, 7.1 Hz, 2H), 7.01 (d, *J* = 15.0 Hz, 1H), 6.94 (s, 1H), 3.68 – 3.61 (m, 8H), 3.63 – 3.54 (m, 4H), 3.38 (s, 3H); ^{13}C NMR (125 MHz, Chloroform-*d*) δ 189.8, 164.1, 137.0, 135.5, 133.6, 133.0, 128.8, 128.8, 71.9, 70.5, 70.4, 70.2, 69.5, 59.0, 39.7; HRMS ESI$^+$ (*m/z*): Calcd. $C_{17}H_{23}NO_5Na^+$ [M + Na]$^+$ 344.1468, found 344.1455.

Proteins used in our study.

Annexin V-Cys315

Sequence:

AQVLRGTVTDFPGFDERADAETLRKAMKGLGTDEESILTLLTSRSNAQRQEISAAFKTL
FGRDLLDDLKSELTGKFEKLIVALMKPSRLYDAYELKHALKGAGTNEKVLTEIIASRTPE
ELRAIKQVYEEEYGSSLEDDVVGDTSGYYQRMLVVLLQANRDPDAGIDEAQVEQDAQ
ALFQAGELKWGTDEEKFITIFGTRSVSHLRKVFDKYMTISGFQIEETIDRETSGNLEQLL
LAVVKSIRSIPAYLAETLYYAMKGAGTDDHTLIRVMVSRSEIDLFNIRKEFRKNFATSLYS
MIKGDTSGDYKKALLLL**C**GEDD

Isotopically Averaged Molecular Weight = 35805 Da

Albumin-Cys34

Sequence:

DAHKSEVAHRFKDLGEENFKALVLIAFAQYLQQ**C**PFEDHVKLVNEVTEFAKTCVADES
AENCDKSLHTLFGDKLCTVATLRETYGEMADCCAKQEPERNECFLQHKDDNPNLPRL
VRPEVDVMCTAFHDNEETFLKKYLYEIARRHPYFYAPELLFFAKRYKAAFTECCQAAD
KAACLLPKLDELRDEGKASSAKQRLKCASLQKFGERAFKAWAVARLSQRFPKAEFAE
VSKLVTDLTKVHTECCHGDLLECADDRADLAKYICENQDSISSKLKECCEKPLLEKSHC
IAEVENDEMPADLPSLAADFVESKDVCKNYAEAKDVFLGMFLYEYARRHPDYSVVLLL
RLAKTYETTLEKCCAAADPHECYAKVFDEFKPLVEEPQNLIKQNCELFEQLGEYKFQN
ALLVRYTKKVPQVSTPTLVEVSRNLGKVGSKCCKHPEAKRMPCAEDYLSVVLNQLCVL
HEKTPVSDRVTKCCTESLVNRRPCFSALEVDETYVPKEFNAETFTFHADICTLSEKERQ
IKKQTALVELVKHKPKATKEQLKAVMDDFAAFVEKCCKADDKETCFAEEGKKLVAASQ
AALGL

Isotopically Averaged Molecular Weight = 66472; with 17 internal disulfides = 66439 Da

C2Am-Cys95

Sequence:

GSPGISGGGGGILDSMVEKLGKLQYSLDYDFQNNQLLVGIIQAAELPA LDMGGTSDPY
VKVFLLPDKKKKFETKVHRKTLNPVFNEQFTFKVPY**C**ELGGKTLVMAVYDFDRFSKHDI
IGEFKVPMNTV DFGHVTEEWR DLQSAEK

Isotopically Averaged Molecular Weight = 16222 Da

Procedure for Annexin V-Cys315 bioconjugation.

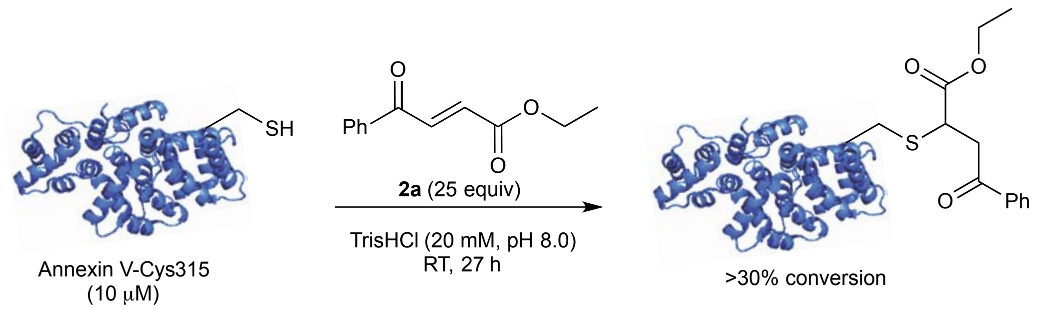

To an eppendorf with 23.17 μL of TrisHCl 20 mM at pH 8.0 was added a 14.40 μL aliquot of a stock solution of Annexin V (27.80 μM, 400 nmol) and the resulting mixture was vortexed for 30 seconds. Afterwards, a 4.11 mM solution of (*E*)-4-oxo-4-phenylbut-2-enoate **2a** (2.42 μL, 25 equiv.) in DMF was added and the reaction mixed for 27 h at room temperature. After, a 10 μL aliquot was analyzed by LC–MS and 30% of conversion to the expected product was observed (calculated mass, 36010; observed mass, 36009). See Supplementary Fig. 9.

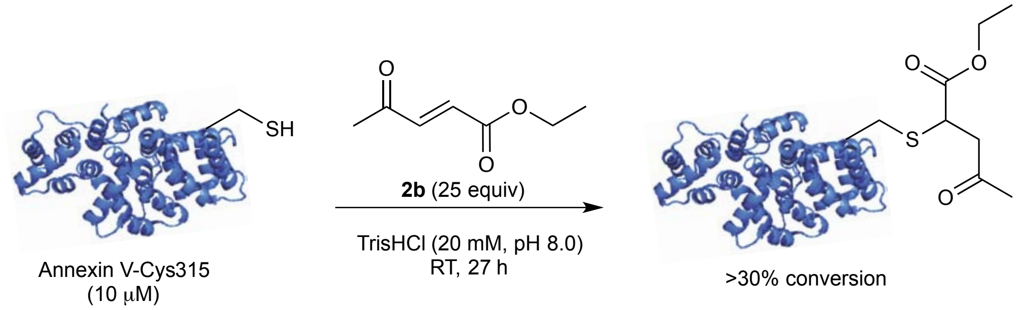

To an eppendorf with 23.73 μL of TrisHCl 20 mM at pH 8.0 was added a 14.40 μL aliquot of a stock solution of Annexin V (27.80 μM, 400 nmol) and the resulting mixture was vortexed for 30 seconds. Afterwards, a 5.34 mM solution of ethyl (*E*)-4-oxopent-2-enoate **2b** (1.87 μL, 25 equiv.) in DMF was added and the reaction mixed for 27 h at room temperature. At the end, a 10 μL aliquot was analyzed by LC–MS and complete conversion to the expected product was observed (calculated mass, 35948; observed mass, 35947). See Supplementary Fig. 10.

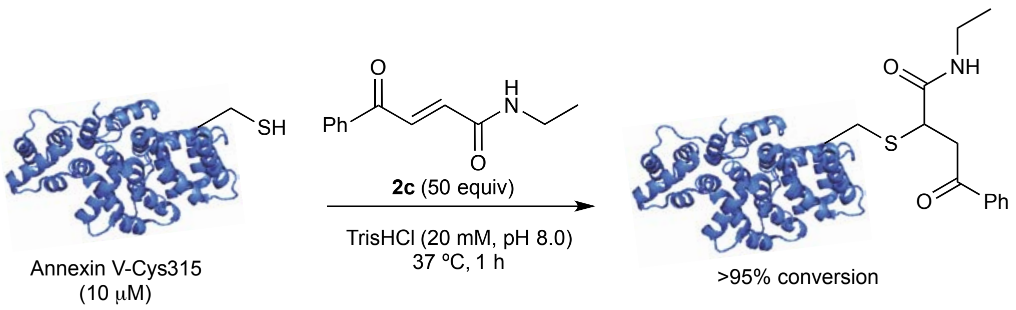

To an eppendorf with 20.26 µL of TrisHCl 20 mM at pH 8.0 was added a 14.40 µL aliquot of a stock solution of Annexin V (27.80 µM, 400 nmol) and the resulting mixture was vortexed for 30 seconds. Afterwards, a 3.74 mM solution of (*E*)-*N*-ethyl-4-oxo-4-phenylbut-2-enamide **2c** (5.34 µL, 50 equiv.) in DMF was added and the reaction mixed for 1 h at 37 ºC. At the end, a 10 µL aliquot was analyzed by LC–MS and complete conversion to the expected product was observed (calculated mass, 36009; observed mass, 36006). See Supplementary Fig. 11.

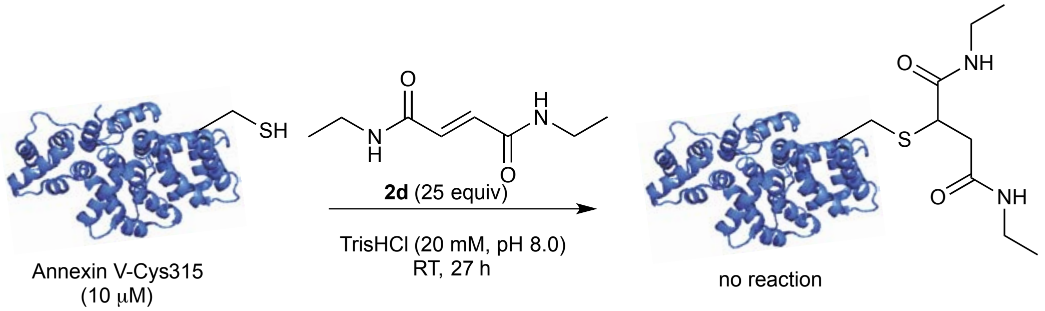

To an eppendorf with 21.73 µL of TrisHCl 20 mM at pH 8.0 was added a 14.40 µL aliquot of a stock solution of Annexin V (27.80 µM, 400 nmol) and the resulting mixture was vortexed for 30 seconds. Afterwards, a 2.58 mM solution of $N_{1,}N_{4}$-diethylfumaramide **2d** (3.87 µL, 25 equiv.) in DMF was added and the reaction mixed for 27 h at room temperature. At the end, a 10 µL aliquot was analyzed by LC–MS and showed only starting protein (calculated mass, 35805). See Supplementary Fig. 12.

Procedure for C2Am-Cys95 bioconjugation

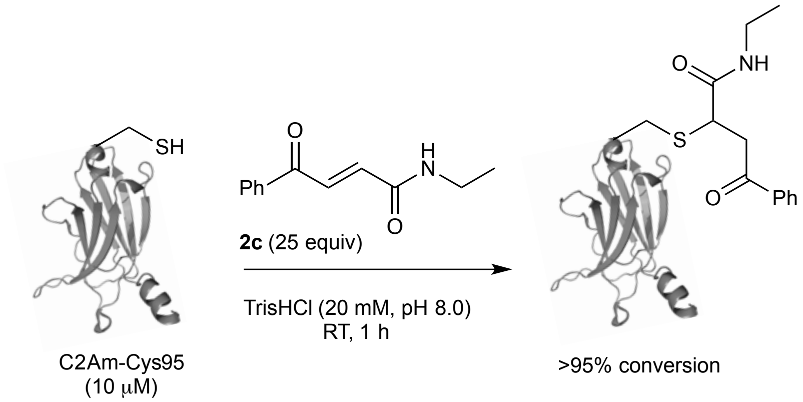

A 3.24 μL aliquot of a stock solution of C2Am (61.64 μM, 200 nmol) was added to an eppendorf containing 15 μL of TrisHCl 20 mM at pH 8.0. TCEP (2.0 mmol, 10 equiv., 0.5 μL of a 40 mM solution) was added and the reaction reacted for 30 min at room temperature. Afterwards, 25 equiv. of a DMF solution of (*E*)-*N*-ethyl-4-oxo-4-phenylbut-2-enamide **2c** (1.34 μL of a 3.74 mM stock solution) was added and the reaction was stirred for 1 h at room temperature. At the end, a 10 μL aliquot was analyzed by LC–MS and complete conversion to the expected product was observed (calculated mass, 16425; observed mass, 16425). See Supplementary Fig. 15.

Procedure for Albumin-Cys34 bioconjugation

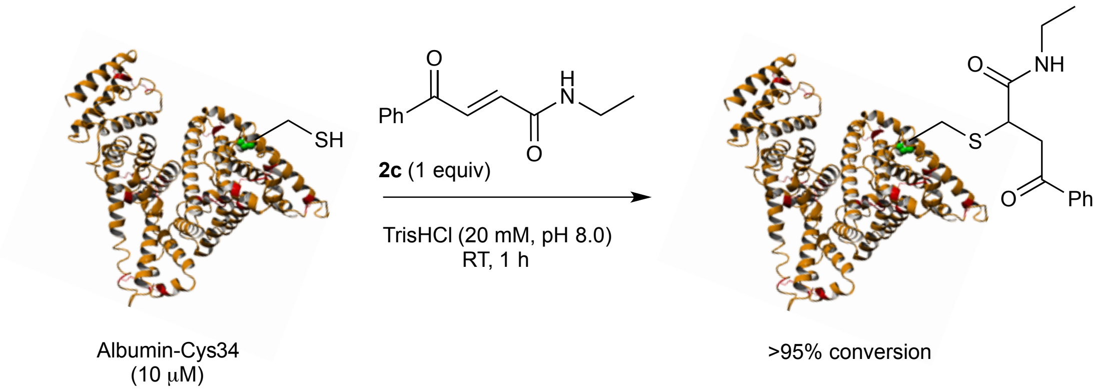

To an eppendorf with 13.40 μL of TrisHCl 20 mM at pH 8.0 was added a 26.5 μL aliquot of a stock solution of Albumin (15.05 μM, 400 nmol) and the resulting mixture was vortexed for 30 seconds. Afterwards, a 3.74 mM solution of (*E*)-*N*-ethyl-4-oxo-4-phenylbut-2-enamide **2c** (0.10 μL, 1 equiv.) in DMF was added and the reaction mixed for 2 h at room temperature. At the end, a 10 μL aliquot was analyzed by LC–MS and complete conversion to the expected product was observed (calculated mass, 66644; observed mass, 66641). See Supplementary Fig. 17.

Chemical controls.

Control: Ellman's reaction with Annexin V-Cys315

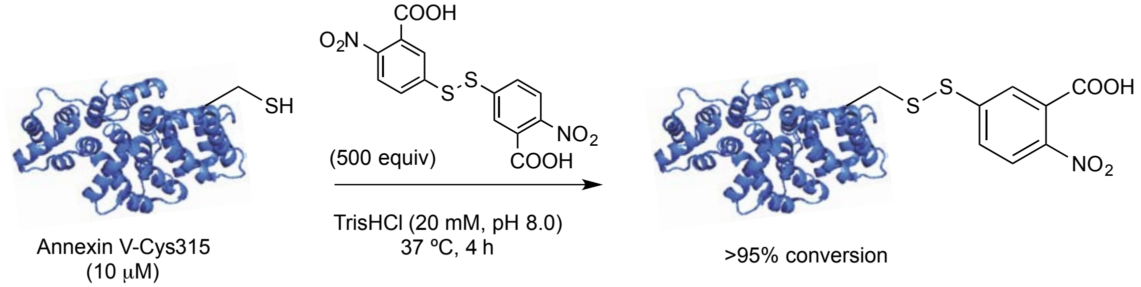

A 14.4 µL aliquot of Annexin V-Cys315 (27.8 µM, 400 nmol) in TrisHCl (20 mM, pH 8.0) was transferred to a 0.5 mL eppendorf tube. A 4 µL aliquot of Ellman's reagent (50.5 mM, 200 µmol, 500 equiv.) was added at room temperature and the resulting mixture vortexed for 30 seconds. After 4 h of additional mixing at 37°C, a 10 µL aliquot was analyzed by LC–MS and full conversion to the expected product (calculated mass, 36003; observed mass, 36002) was observed. Small molecules were removed from the reaction mixture by loading the sample onto a Zeba Spin Desalting Column previously equilibrated with TrisHCl (20 mM, pH 8.0). The sample was eluted *via* centrifugation (2 min, 1000xg). The protein sample was used to the next reaction control. See Supplementary Fig. 18.

Control: Annexin V-SS-Ellman's reaction with 2c

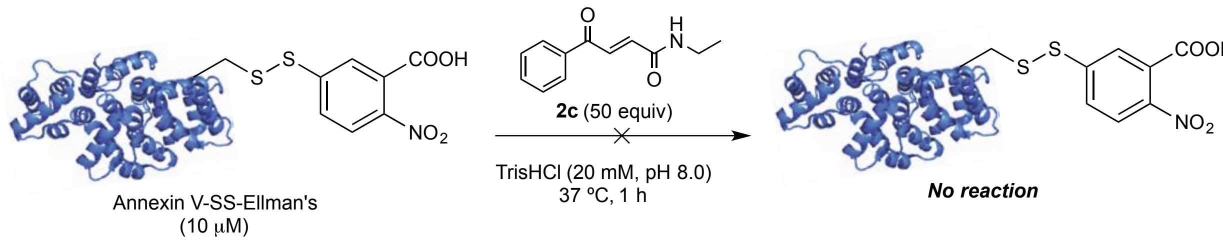

A 26 µL aliquot of Annexin V-SS-Ellman's (10 µM, 260 nmol) in TrisHCl (20 mM, pH 8.0) was transferred to a 0.5 mL eppendorf tube. A 4 µL aliquot of compound **2c** (3.72 mM, 50 equiv.) was added at room temperature and the resulting mixture vortexed for 30 seconds. After 1 h of additional mixing at 37 ºC, a 10 µL aliquot was analyzed by LC–MS and no reaction observed. The protein sample was stored at –20 ºC.
See Supplementary Fig. 19.

Control: Annexin V conjugate reaction with Ellman's Reagent

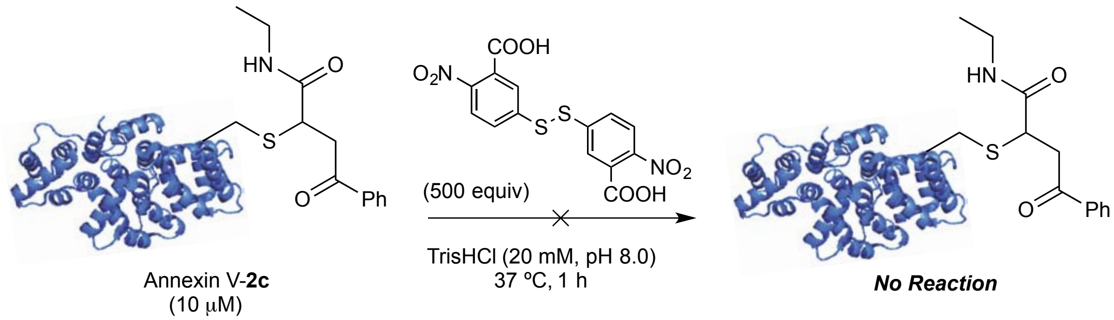

A 13.5 μL aliquot of the conjugate Annexin V-**2c** (10 μM, 135 nmol) in TrisHCl (20 mM, pH 8.0) was transferred to a 0.5 mL eppendorf tube. A 1.5 μL aliquot of Ellman's reagent (50.5 mM, 500 equiv.) was added at room temperature and the resulting mixture vortexed for 30 seconds. After 1 h of additional mixing at 37 ºC, a 10 μL aliquot was analyzed by LC–MS and no conversion to the expected product was observed.
See Supplementary Fig. 20.

Stability of the conjugate albumin-2c in human plasma.

A 20 µL aliquot of the conjugate albumin-**2c** (10 µM) in 20 mM TrisHCl buffer at pH 8.0 was thawed. 2 µL of reconstituted human plasma (Sigma Aldrich) was added at room temperature and the resulting mixture vortexed for 30 seconds. The resulting reaction mixture was then mixed at 37 ºC overnight. After 1 and 24 h, a 5 µL aliquot of each reaction mixture was analyzed by LC–MS (5 µL aliquot diluted with 5 µL of 20 mM Tris HCl buffer at pH 8.0). No significant degradation of the adduct was observed at 48 h. Similar data was obtained when we incubated the albumin conjugate in the presence of GSH (10 mM) (data not shown). See Supplementary Fig. 21.

Assessment of the FcRn binding properties of albumin-2c

Immobilisation summary

Instrument: Biacore 3000; Chip: CM5; Running buffer: HBS-EP (GE Healthcare); Ligand: shFcRn (BSP biotin) 760 µg/mL diluted to 10 µg/mL; Dilution buffer: Na Acetate buffer pH 4.5 (GE Healthcare); Coupling method: EDC/NHS (amine coupling); Wash buffer: 1 M Ethanolamine (GE Healthcare); Flow path: FC2; Ligand level: 1362RU

Immobilisation method

Immobilisation was carried out using GE Healthcare amine coupling kit (following manufacturer's instructions) and using an Immobilisation wizard that aimed to reach desired level of ligand used. CM5 chip surface was activated with NHS and EDC, diluted 1:1. Ligand was flowed over chip at a constant flow rate of 5 µL/min. Surface of reference cell was activated with NHS:EDC and deactivated with ethanolamine but left blank for reference.

Chip surface was left to stabilise with a constant flow (5 µL/min) of running buffer - HBS-EP buffer (0.01 M HEPES, 3 mM EDTA, 0.15 M NaCl, 0.005% surfactant P20) at pH 7.4 (GE Healthcare) at 25 °C for ~2 hours or overnight.

Binding Analysis pH 5.5 Method

Albumins were diluted to 10 µM (in running buffer – 67 mM Phosphate buffer, 0.15 M Na Cl, 0.005% Tween 20 pH 5.5) and flowed over immobilised shFcRn, at a constant flow rate (30 µL/min) for one minute, with 2.5 minutes delay before washing (with running buffer). Regeneration using HBS-EP buffer pH 7.4 for 12 seconds restored the baseline between injections.

Sensorgrams for binding data were obtained using BIAevaluation software 4.1 (BIAcore AB). Data was reference cell subtracted and zero-adjusted.

Kinetic Analysis Method

Albumins were 8-step serially diluted 1:1 (10 μM – 0.156 μM + 0 μM) down a microtitre plate in running buffer and flowed over immobilised shFcRn to obtain kinetic data and confirm K_D values. Flow rate was 30 μL/min and injection time was 60 s with a 60 s delay before washing. All injections required a 12 s regeneration pulse (HBS-EP) post injection to restore the baseline.

Kinetic parameters were extrapolated using BIAevaluation 1:1 Langmuir model (BIAcore AB). Data was reference cell adjusted and zero-adjusted.

See Supplementary Fig. 22.

Labelling of albumin with the fluorescent reagent 7.

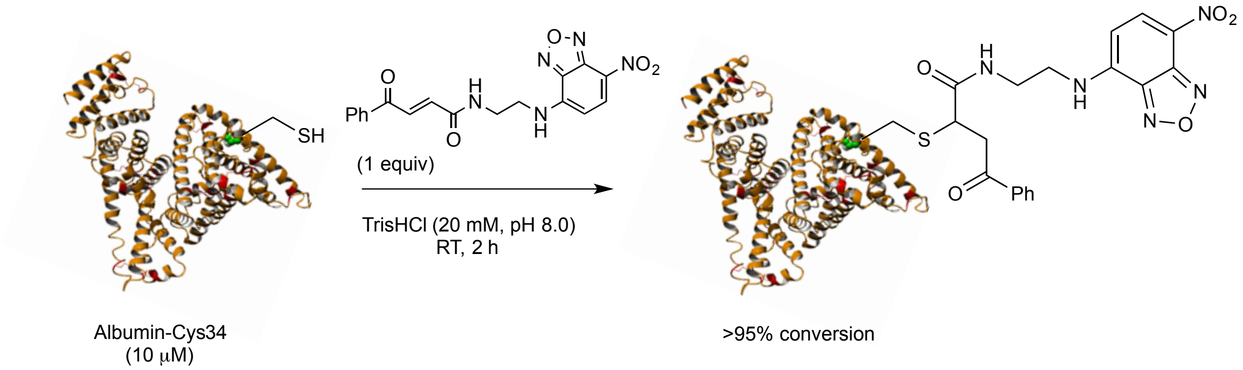

To an eppendorf with 49.43 µL of TrisHCl 20 mM at pH 8.0 was added a 0.33 µL aliquot of a stock solution of albumin (1505 µM, 500 nmol) and the resulting mixture was vortexed for 30 seconds. Afterwards, a 4.19 mM solution of **7** (0.12 µL, 1 equiv.) in DMF was added and the reaction mixed for 2 h at room temperature. At the end, a 10 µL aliquot was analyzed by LC–MS and complete conversion to the expected product was observed (calculated mass, 66822; observed mass, 66821). See Supplementary Figs 23 and 24.

Labelling of albumin with the PEG reagent 8.

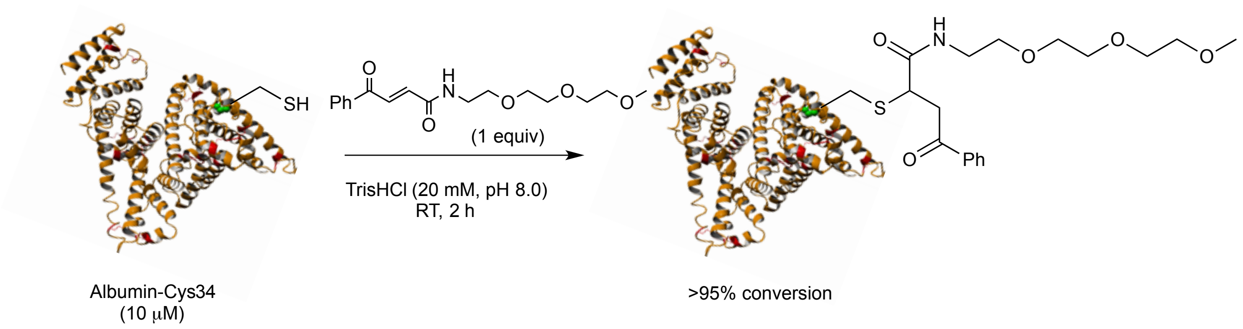

To an eppendorf with 13.18 μL of TrisHCl 20 mM at pH 8.0 was added a 26.57 μL aliquot of a stock solution of albumin (15.05 μM, 400 nmol) and the resulting mixture was vortexed for 30 seconds. Afterwards, a 2.74 mM solution of **8** (0.13 μL, 1 equiv.) in DMF was added and the reaction mixed for 2 h at room temperature. At the end, a 10 μL aliquot was analyzed by LC–MS and complete conversion to the expected product was observed (calculated mass, 66762; observed mass, 66760). See Supplementary Fig. 25.

Labelling of Annexin V-Cys315 with fluorescent reagent 7.

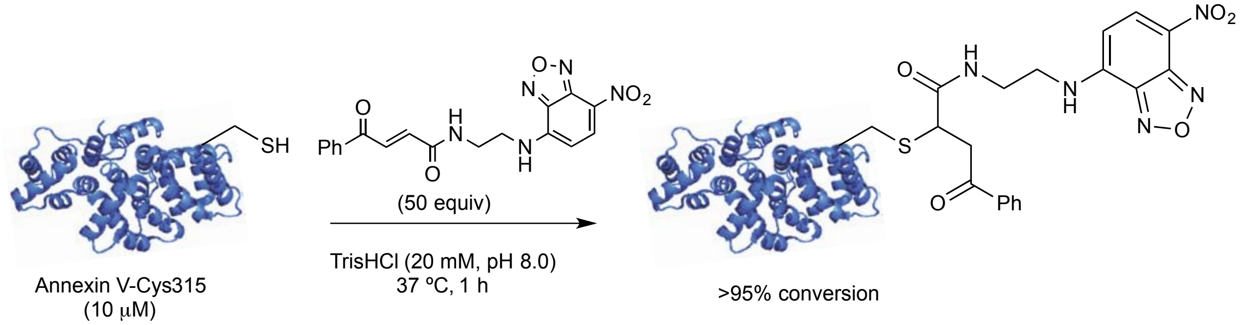

To an eppendorf with 20.84 µL of TrisHCl 20 mM at pH 8.0 was added a 14.40 µL aliquot of a stock solution of Annexin V (27.8 µM, 400 nmol) and the resulting mixture was vortexed for 30 seconds. Afterwards, a 4.19 mM solution of **7** (4.76 µL, 50 equiv.) in DMF was added and the reaction mixed for 1 h at 37 ^{o}C. At the end, a 10 µL aliquot was analyzed by LC–MS and complete conversion to the expected product was observed (calculated mass, 36187; observed mass, 36185). See Supplementary Figs 26 and 27.

Labelling of C2Am with fluorescent reagent 7.

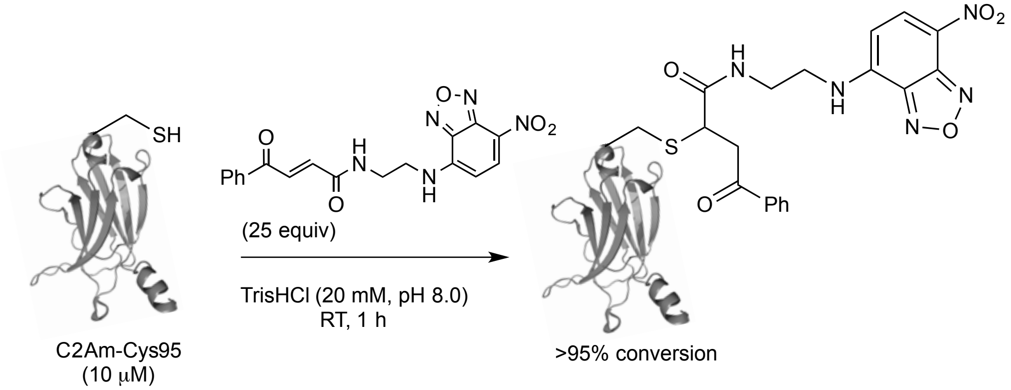

A 6.49 μL aliquot of a stock solution of C2Am (61.64 μM, 400 nmol) was added to an eppendorf containing 26.10 μL of TrisHCl 20 mM at pH 8.0. TCEP (20.0 mmol, 50 equiv., 5 μL of a 40 mM solution) was added and the reaction reacted for 1 h at room temperature. Afterwards, 25 equiv. of a DMF solution of **7** (2.88 μL of a 4.19 mM stock solution) was added and the reaction was stirred for 1 h at room temperature. At the end, a 10 μL aliquot was analyzed by LC–MS and complete conversion to the expected product was observed (calculated mass, 16603; observed mass, 16603). See Supplementary Figs 28 and 29.

Procedure for Trastuzumab bioconjugation.

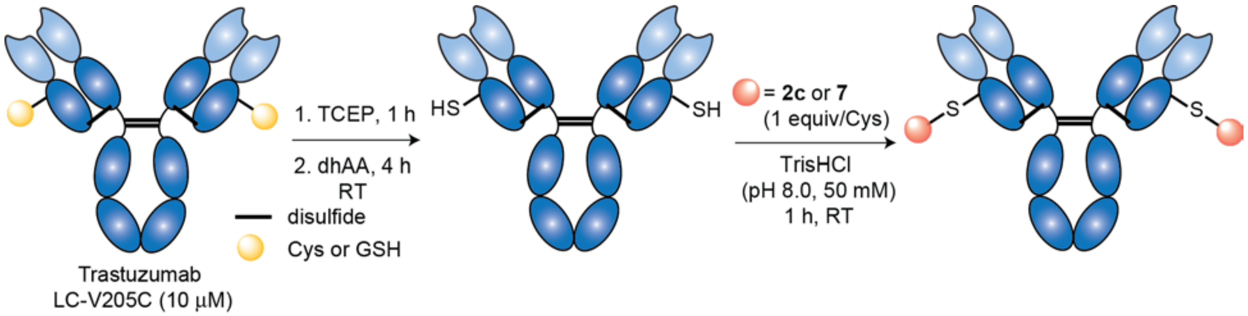

Disulfide reduction

Trastuzumab reduction and oxidation was performed following an adapted reported procedure.[11] Trastuzumab (15.33 μL, 1 equiv., 400 nmol) disulfide bonds were reduced by mild reduction in TrisHCl pH 8 (23.46 μL) at 25 °C by the addition of tenfold molar excess reducing agent TCEP (1 μL, 4000 μM) for 1.5 h, followed by filtration using a Zeba Spin Desalting Column previously equilibrated with 20 mM TrisHCl (20 mM) at pH 8.0. The sample was eluted *via* centrifugation (2 min, 1500xg). Sample was analyzed by LC-MS (calculated mass, 23441; observed mass, 23443). See Supplementary Figs 30 and 31.

Disulfide re-oxidation

To re-form the interchain disulfide bonds, the reduced Trastuzumab was incubated for 4 h at 25 °C with dhAA (Sigma-Aldrich) at a tenfold molar excess. After, a 5μL aliquot of reaction mixture was analyzed by LC–MS (5μL aliquot diluted with 5μL of 20 mM TrisHCl buffer at pH 8.0).

Bioconjugation with 2c

To an eppendorf with 19.51 μL of re-oxidized Trastuzumab was added a 0.48 μL aliquot of a stock solution of (*E*)-*N*-ethyl-4-oxo-4-phenylbut-2-enamide **2c** in DMF and the reaction mixed for 1 h at room temperature. At the end, a 10 μL aliquot was analyzed by LC–MS and complete conversion to the expected product was observed (calculated mass, 23646; observed mass, 23646). See Supplementary Figs 32 and 33.

Activity by enzyme-linked immunosorbent assay (ELISA).

Binding affinity to HER2 receptor was determined by ELISA, using a Trastuzumab (Herceptin™) ELISA Kit-96 wells provided by Generon. Antibodies to Trastuzumab are pre-coated onto microwells. Different concentrations of the Trastuzumab-**2c** and unmodified Trastuzumab in the sample buffer provided by the kit were pipetted into the microwells (0 nM, 0.06 nM, 0.12 nM, 0.25 nM, 0.50 nM, 1.00 nM and 2.0 nM) and were incubated for 1 h at room temperature. Then, the solutions were removed and the wells washed with the wash solution three times. Then, a HRP (horseradish peroxidase) conjugated anti-Human IgG was pipetted and incubated with the samples. After removing the solutions and washing again the microwells with the wash solution three times, the ready to use substrate solution (TMB) was added to the microwells and color develops proportionally to the amount of Trastuzumab in the sample. Color development was stopped after 30 min by addition of a stop solution. Absorbance was measured at 450 nm. Absorbance was corrected by subtracting average of negative controls. See Supplementary Fig. 35.

Supplementary Discussion

Antibody conjugates specificity by flow cytometry. To assess whether the Trastuzumab-**7** retained the her2/c-erb-2 specificity, we used this modified antibody to stain cells that overexpress the her2/c-erb-2 gene product (SKBR3), as well as cells that express low levels of this protein (HepG2). We incubated these two cell-lines with the modified antibody and quantified the percentage of the population that was stained by flow-cytometry, after exposure to increasing concentrations of modified antibody. Our results show that after a 1 h treatment with the modified antibody at 150 nM, >90% of the SKBR3 cells are stained, while only & 20% of the HepG2 cells are positive for this marker. These results are in line with what has been previously published regarding anti-her2 antibody staining scores (ref. 29 of the manuscript). At higher concentrations, both cell lines showed high staining levels, indicative of unspecific binding of the antibody, while at concentrations lower than 10 nM there was no observable staining (data not shown). Our results clearly show that the Trastuzumab-**7** retains its her2/c-erb-2 specificity, at working concentrations similar to those previously described.

Supplementary References

(1) Frisch, M. J.; Trucks, G. W.; Cheeseman, J. R.; Scalmani, G.; Caricato, M.; Hratchian, H. P.; Li, X.; Barone, V.; Bloino, J.; Zheng, G.; Vreven, T.; Montgomery, J. A.; Petersson, G. A.; Scuseria, G. E.; Schlegel, H. B.; Nakatsuji, H.; Izmaylov, A. F.; Martin, R. L.; Sonnenberg, J. L.; Peralta, J. E.; Heyd, J. J.; Brothers, E.; Ogliaro, F.; Bearpark, M.; Robb, M. A.; Mennucci, B.; Kudin, K. N.; Staroverov, V. N.; Kobayashi, R.; Normand, J.; Rendell, A.; Gomperts, R.; Zakrzewski, V. G.; Hada, M.; Ehara, M.; Toyota, K.; Fukuda, R.; Hasegawa, J.; Ishida, M.; Nakajima, T.; Honda, Y.; Kitao, O.; Nakai, H. *Gaussian Inc. Wallingford CT 2009*.

(2) Zhao, Y. & Truhlar, D. G. The M06 Suite of Density Functionals for Main Group Thermochemistry, Thermochemical Kinetics, Noncovalent Interactions, Excited States, and Transition Elements: Two New Functionals and Systematic Testing of Four M06-Class Functionals and 12 Other Functionals. *Theor. Chem. Acc.* **120**, 215–241, (2007).

(3) Scalmani, G. & Frisch, M. J. Continuous Surface Charge Polarizable Continuum Models of Solvation. I. General Formalism. *J. Chem. Phys.* **132**, 114110 (2010).

(4) Ribeiro, R. F.; Marenich, A. V.; Cramer, C. J. &; Truhlar, D. G. Use of Solution-Phase Vibrational Frequencies in Continuum Models for the Free Energy of Solvation. *J. Phys. Chem. B* **115**, 14556–14562 (2011).

(5) Gonzalez, C. & Schlegel, H. B. An Improved Algorithm for Reaction Path Following. *J. Chem. Phys.* **90**, 2154–2161 (1989).

(6) Gonzalez, C. & Schlegel, H. B. Reaction Path Following in Mass-Weighted Internal Coordinates. *J. Phys. Chem.* **94**, 5523–5527 (1990).

(7) Abbasov, M. E.; Hudson, B. M.; Tantillo, D. J. & Romo, D. Acylammonium Salts as Dienophiles in Diels–Alder/Lactonization Organocascades. *J. Am. Chem. Soc.* **136**, 4492–4495 (2014).

(8) Diwischek, F. Development of Synthesis Pathways and Characterization of Cerulenin Analogues as Inhibitors of the Fatty Acid Biosynthesis of Mycobacterium Tuberculosis and of Efflux Pump Resistant Candida Albicans. Doctoral Thesis, Julius-Maximilians-Universität Würzburg: Würzburg, Germany, 2008.

(9) Kalia, D.; Malekar, P. V. & Parthasarathy, M. Exocyclic Olefinic Maleimides: Synthesis and Application for Stable and Thiol-Selective Bioconjugation. *Angew. Chem. Int. Ed.* **55**, 1432–1435 (2015).

(10) Oohashi, T.; Kakuta, H. Lysine Oligomer Derivative and Cartilage Tissue Marker Made Thereof. EP2826785 (A1), December 2, 2015.

(11) Junutula, J. R. *et al.* Site-Specific Conjugation of a Cytotoxic Drug to an Antibody Improves the Therapeutic Index. *Nat. Biotechnol.* **26**, 925–932 (2008).
