# Supplementary material for: Stoichiometric and irreversible cysteine-selective protein modification using carbonylacrylic reagents
Source: Nat Commun. 2016 Oct 26;7:13128. doi: 10.1038/ncomms13128 (PMC5095172; doi:10.1038/ncomms13128)
Supplement: Supplementary Dataset 1 — Cartesian coordinates of the lowest energy calculated structures. [file ncomms13128-s2.doc]

**Supplementary Data 1** Cartesian coordinates of the lowest energy calculated structures.

**Structure 2a**

C 1.079713 0.894107 -0.075052

C -0.116645 0.011037 -0.251271

H 0.008814 -1.007925 -0.604077

C -1.340121 0.473302 0.011181

H -1.501480 1.485914 0.367635

C -2.532726 -0.397569 -0.178831

O -3.655758 0.248530 0.133705

C -4.885546 -0.493230 -0.013120

H -4.970269 -0.813488 -1.054957

H -4.826600 -1.383298 0.618845

C -6.016214 0.422931 0.395651

H -6.045920 1.309661 -0.241955

H -6.966452 -0.106976 0.296403

H -5.902525 0.738314 1.435523

O -2.497023 -1.546994 -0.568302

C 2.431199 0.266844 -0.004215

C 2.605653 -1.101198 0.238282

C 3.553096 1.091958 -0.161334

C 3.889966 -1.635638 0.324526

H 1.751416 -1.754117 0.382444

C 4.832510 0.554208 -0.088632

H 3.403511 2.150771 -0.345766

C 5.001650 -0.811380 0.155731

H 4.020726 -2.694228 0.522394

H 5.698419 1.194371 -0.221383

H 6.000754 -1.231305 0.214950

O 0.942080 2.105974 -0.001347

**Structure 2a_ts1**

C 0.884411 -0.813302 0.741885

C -0.272241 -0.082960 0.137701

H -0.118434 0.330923 -0.855902

C -1.571289 -0.510212 0.439831

H -1.761723 -1.137484 1.301413

C -2.662697 -0.023464 -0.326511

O -3.870369 -0.531460 0.082571

C -5.019997 -0.107849 -0.651916

H -5.100169 0.982990 -0.605097

H -4.903848 -0.388976 -1.703801

C -6.228774 -0.777642 -0.031607

H -6.336032 -0.487427 1.016974

H -7.134386 -0.480175 -0.566687

H -6.137891 -1.865863 -0.084364

O -2.614408 0.767763 -1.279282

O 0.756524 -1.497703 1.750875

S 0.390582 1.814202 1.128984

C 1.512462 2.528032 -0.117533

H 1.550643 3.611758 0.016393

H 2.523065 2.121252 -0.028216

H 1.139472 2.321466 -1.126211

C 2.229609 -0.699275 0.083620

C 2.373252 -0.536486 -1.297657

C 3.371223 -0.787898 0.888378

C 3.645223 -0.463869 -1.866335

H 1.500434 -0.482698 -1.940656

C 4.639435 -0.691945 0.324231

H 3.247620 -0.917834 1.959245

C 4.778111 -0.531478 -1.056955

H 3.748892 -0.350050 -2.940655

H 5.519817 -0.742090 0.957093

H 5.766891 -0.461743 -1.499369

**Structure 2a_ts2**

C -1.521881 0.448869 -0.534581

C -0.444623 -0.313734 0.054246

H -0.654269 -1.076498 0.794826

C 0.855908 -0.007664 -0.244848

C 1.949867 -0.895367 0.226656

O 3.054493 -0.752408 -0.519450

C 4.216591 -1.475482 -0.081893

H 4.469294 -1.137521 0.928999

H 3.976751 -2.541289 -0.037296

C 5.326230 -1.186076 -1.068456

H 5.540263 -0.114868 -1.106801

H 6.235114 -1.710633 -0.763919

H 5.049984 -1.524872 -2.070122

O 1.859536 -1.702175 1.134356

O -1.325270 1.441773 -1.253037

H 1.081545 0.638079 -1.086548

S 1.931460 1.808081 1.182106

C 3.271227 2.266915 0.027718

H 4.176968 1.685405 0.217575

H 2.963178 2.077312 -1.006427

H 3.511409 3.329372 0.120391

C -2.940170 0.031566 -0.249913

C -3.959816 0.973717 -0.432390

C -3.275567 -1.261915 0.168921

C -5.288137 0.636644 -0.187430

H -3.691525 1.971550 -0.764409

C -4.606849 -1.604271 0.401957

H -2.504135 -2.014933 0.293437

C -5.614741 -0.655202 0.230059

H -6.069222 1.378456 -0.321826

H -4.856731 -2.612915 0.715543

H -6.650293 -0.921470 0.417656

**Structure 2a_add1**

C 1.380760 0.648206 0.626944

C 0.220194 1.285009 -0.120369

H 0.228686 0.953551 -1.162180

C -1.035106 0.806696 0.521736

H -1.176507 0.954270 1.586505

C -1.988028 0.128025 -0.225749

O -3.093651 -0.271396 0.520838

C -4.118056 -0.961737 -0.185434

H -4.496693 -0.334596 -1.000177

H -3.712240 -1.875308 -0.634051

C -5.217516 -1.285332 0.807101

H -5.625642 -0.369935 1.244537

H -6.029163 -1.820288 0.306424

H -4.835944 -1.915346 1.615413

O -1.951915 -0.161933 -1.448558

O 1.956770 1.210247 1.547243

S 0.315065 3.121656 -0.142326

C 1.952604 3.372680 -0.891265

H 2.073517 4.441383 -1.076226

H 2.740625 3.032564 -0.216787

H 2.022739 2.839953 -1.842964

C 1.776374 -0.752510 0.239218

C 1.054588 -1.516044 -0.689507

C 2.929779 -1.290104 0.823047

C 1.488410 -2.798718 -1.020328

H 0.142105 -1.128274 -1.136847

C 3.363642 -2.569407 0.485266

H 3.479863 -0.689665 1.540439

C 2.641962 -3.326902 -0.438344

H 0.921908 -3.389080 -1.733792

H 4.261980 -2.974996 0.939965

H 2.976472 -4.325251 -0.703124

**Structure 2a_add2**

C -1.192729 -0.324172 0.664679

C -0.186168 -0.344336 -0.283906

H -0.386262 -0.161203 -1.333598

C 1.205124 -0.719061 0.103878

C 2.178490 0.262996 -0.515953

O 2.558336 1.193922 0.369533

C 3.398403 2.251938 -0.131774

H 4.306533 1.807563 -0.548213

H 2.866134 2.765348 -0.937560

C 3.700044 3.176188 1.026653

H 4.222567 2.640446 1.822992

H 4.337382 3.995266 0.684704

H 2.778922 3.600752 1.433302

O 2.549903 0.264807 -1.672438

O -1.050459 -0.617417 1.902987

H 1.292404 -0.667974 1.191949

S 1.591661 -2.444216 -0.410353

C 3.318965 -2.572750 0.142128

H 3.957597 -1.888371 -0.421645

H 3.397034 -2.359325 1.210663

H 3.650610 -3.596182 -0.040128

C -2.590336 0.050728 0.210094

C -3.678845 -0.383345 0.976480

C -2.849420 0.823802 -0.930699

C -4.988325 -0.079025 0.605284

H -3.475670 -0.964635 1.870224

C -4.156504 1.136157 -1.302008

H -2.023931 1.203451 -1.525127

C -5.233414 0.681950 -0.538194

H -5.818222 -0.433826 1.209779

H -4.334521 1.743163 -2.184935

H -6.251104 0.926056 -0.827388

**Structure 2b**

C 2.964902 0.056396 -0.000075

C 1.599268 -0.544088 -0.000051

H 1.562600 -1.631046 -0.000054

C 0.470007 0.167817 -0.000054

H 0.453266 1.253277 -0.000051

C -0.850753 -0.522852 0.000011

O -1.852513 0.355994 0.000447

C -3.188234 -0.192305 0.000727

H -3.300176 -0.822324 -0.885602

H -3.300673 -0.820422 0.888367

C -4.152929 0.971259 -0.000784

H -4.012419 1.589775 -0.890393

H -5.177690 0.592536 -0.000649

H -4.012892 1.591669 0.887576

O -1.003408 -1.726902 -0.000154

O 3.930976 -0.688969 -0.000335

C 3.115208 1.554904 0.000245

H 2.634181 1.985243 -0.882917

H 2.634092 1.984780 0.883588

H 4.174455 1.808735 0.000355

**Structure 2b_ts1**

C 2.032456 1.193717 -0.385746

C 0.887252 0.242802 -0.418171

C -0.386700 0.633068 -0.006403

H -0.543857 1.489458 0.638511

C -1.512798 -0.179206 -0.334587

O -2.688900 0.334559 0.140469

C -3.873176 -0.410958 -0.152659

H -3.985904 -0.499709 -1.237935

H -3.775713 -1.421165 0.257517

C -5.040746 0.328392 0.466759

H -5.126994 1.334918 0.048979

H -5.970208 -0.210326 0.265147

H -4.916295 0.409569 1.549806

O -1.507387 -1.236763 -0.974247

O 2.901216 1.152287 -1.252680

C 2.052649 2.263597 0.680887

H 1.463571 3.116124 0.323417

H 1.617206 1.914672 1.618136

H 3.078930 2.595734 0.842696

H 0.950244 -0.485389 -1.224466

S 2.056808 -1.106070 1.040449

C 3.218588 -1.836484 -0.155648

H 3.749250 -1.041554 -0.689831

H 3.951749 -2.453428 0.369130

H 2.694515 -2.462000 -0.883227

**Structure 2b_ts2**

C 3.020778 -0.303935 0.437303

C 1.813540 -0.745298 -0.216760

H 1.885772 -1.424272 -1.060117

C 0.588046 -0.258001 0.167878

C -0.652056 -0.877541 -0.367638

O -1.702028 -0.652778 0.436105

C -2.979595 -1.101864 -0.044203

H -3.184029 -0.599509 -0.995790

H -2.932264 -2.178590 -0.228297

C -4.006337 -0.752970 1.010577

H -4.027829 0.325482 1.187543

H -4.997737 -1.069766 0.677807

H -3.779178 -1.258653 1.952449

O -0.722909 -1.553338 -1.378186

O 3.031906 0.541845 1.347200

C 4.335736 -0.890484 -0.039360

H 5.004126 -1.035905 0.812052

H 4.201553 -1.833672 -0.572546

H 4.811911 -0.176594 -0.720701

H 0.492926 0.267388 1.112534

S -0.125446 1.868637 -0.918827

C -1.363709 2.404789 0.312284

H -2.356584 2.015027 0.074400

H -1.090303 2.040878 1.308720

H -1.415401 3.495991 0.351738

**Structure 2b_add1**

C 1.796938 1.265556 -0.233621

C 1.099096 -0.030394 0.140007

H 0.977496 -0.061286 1.228428

C -0.254245 0.014525 -0.478299

H -0.344730 0.170362 -1.547495

C -1.390858 -0.132495 0.306189

O -2.574255 -0.064948 -0.429420

C -3.785337 -0.188079 0.306013

H -3.810534 -1.151372 0.827650

H -3.842957 0.599385 1.065856

C -4.931404 -0.075138 -0.680272

H -4.874723 -0.867503 -1.431860

H -5.887318 -0.163856 -0.156757

H -4.906950 0.890621 -1.192764

O -1.467611 -0.308763 1.547677

O 2.517974 1.384897 -1.211373

C 1.509655 2.436954 0.677653

H 0.515123 2.362099 1.121720

H 2.247019 2.415468 1.489075

H 1.617444 3.376959 0.134420

S 2.038925 -1.530977 -0.360567

C 3.627017 -1.227881 0.472292

H 4.245123 -2.118734 0.349001

H 4.137849 -0.371787 0.027295

H 3.470306 -1.052513 1.539648

**Structure 2b_add2**

C -2.313140 -1.208738 -0.157813

C -1.417053 -0.456316 0.580326

H -1.432818 -0.478859 1.665723

C -0.429190 0.438734 -0.092839

C 0.943685 0.199690 0.500125

O 1.650109 -0.662494 -0.244617

C 2.927889 -1.067907 0.282368

H 3.548019 -0.177426 0.417041

H 2.769423 -1.527300 1.262257

C 3.537079 -2.037813 -0.705363

H 3.677871 -1.561467 -1.678738

H 4.511331 -2.370182 -0.338701

H 2.897237 -2.914600 -0.831740

O 1.365801 0.668661 1.538427

O -2.394432 -1.233142 -1.436949

C -3.296004 -2.091504 0.602223

H -3.169183 -3.132905 0.287025

H -3.170393 -2.031977 1.686675

H -4.320570 -1.799160 0.347768

H -0.402720 0.189245 -1.156831

S -0.918838 2.205979 0.060337

C 0.475398 3.014901 -0.780882

H 1.402950 2.865743 -0.223063

H 0.586682 2.629266 -1.796996

H 0.259773 4.083483 -0.829569

**Structure 2c**

C 1.099401 0.895235 -0.050887

C -0.096578 0.015216 -0.218416

H 0.025206 -1.013130 -0.543495

C -1.322935 0.485358 0.015993

H -1.462504 1.513015 0.344230

C -2.522577 -0.390100 -0.167600

C -4.964992 -0.519128 -0.026896

H -5.059219 -0.877153 -1.057553

H -4.938236 -1.401582 0.621130

C -6.127474 0.388927 0.340727

H -6.164732 1.262407 -0.316553

H -7.069129 -0.153904 0.238885

H -6.042974 0.733376 1.375457

O -2.433602 -1.561621 -0.542968

C 2.452414 0.266052 0.003492

C 2.629687 -1.099728 0.256050

C 3.572870 1.087430 -0.180471

C 3.914670 -1.635307 0.325933

H 1.776834 -1.749488 0.420940

C 4.852884 0.548608 -0.124593

H 3.421603 2.144683 -0.372438

C 5.024646 -0.814674 0.130206

H 4.047246 -2.692129 0.532149

H 5.717167 1.186299 -0.278574

H 6.024078 -1.235498 0.176529

O 0.969718 2.108659 0.029060

N -3.702486 0.197005 0.105305

H -3.718948 1.160440 0.412193

**Structure 2c_ts1**

C -1.069484 0.717264 -0.935574

C 0.316289 0.312193 -0.532842

C 0.615204 -1.074551 -0.413799

H -0.173586 -1.807088 -0.296212

C 1.955102 -1.552485 -0.337138

C 4.319105 -0.990861 0.073163

H 4.373870 -1.526701 1.032269

H 4.644882 -1.693653 -0.697845

C 5.231725 0.227373 0.101983

H 4.908975 0.939888 0.868778

H 6.258790 -0.068397 0.330185

H 5.225065 0.739869 -0.864426

O 2.283922 -2.766990 -0.378435

O -1.227634 1.584675 -1.787735

H 1.035608 0.902989 -1.104300

S 0.487109 1.533676 1.214107

C 0.885946 3.088467 0.357617

H 0.064970 3.380206 -0.303201

H 1.039463 3.875728 1.098137

H 1.800341 2.983358 -0.234170

C -2.267174 0.051159 -0.320553

C -2.219707 -0.600988 0.917880

C -3.476864 0.104301 -1.022180

C -3.368469 -1.187443 1.444242

H -1.289852 -0.636132 1.475746

C -4.622413 -0.492196 -0.501169

H -3.504258 0.613664 -1.980218

C -4.569506 -1.137971 0.734808

H -3.326606 -1.682280 2.409244

H -5.553970 -0.453584 -1.056724

H -5.461929 -1.600002 1.145505

N 2.954193 -0.585268 -0.223951

H 2.656315 0.263138 0.249909

**Structure 2c_ts2**

C -1.513839 0.445222 -0.522101

C -0.432474 -0.283436 0.092374

H -0.635063 -1.038609 0.842372

C 0.870825 0.034419 -0.196650

C 1.966742 -0.868591 0.275418

C 4.306775 -1.528495 -0.041071

H 4.648967 -1.116886 0.917830

H 4.056825 -2.579307 0.129883

C 5.391132 -1.396788 -1.099075

H 5.651767 -0.346899 -1.266298

H 6.293816 -1.920299 -0.777351

H 5.062601 -1.828256 -2.048747

O 1.834882 -1.637322 1.232643

O -1.331717 1.429734 -1.259149

H 1.071901 0.661779 -1.061388

S 1.844213 1.930688 1.156200

C 3.149278 2.421306 -0.023784

H 4.063784 1.838552 0.121548

H 2.809704 2.280316 -1.057853

H 3.397587 3.479303 0.093817

C -2.928437 0.003414 -0.249232

C -3.965501 0.917371 -0.472243

C -3.244032 -1.286576 0.195379

C -5.290609 0.557070 -0.242484

H -3.713134 1.912224 -0.825035

C -4.571459 -1.652444 0.414509

H -2.458917 -2.019250 0.351329

C -5.596854 -0.730851 0.201436

H -6.085072 1.277722 -0.409863

H -4.804657 -2.658466 0.748866

H -6.629609 -1.015551 0.377091

N 3.099801 -0.833174 -0.464067

H 3.188893 -0.114166 -1.169744

**Structure 2c_add1**

C 1.390421 0.654868 0.628154

C 0.224253 1.275418 -0.120211

H 0.228058 0.939868 -1.160704

C -1.020683 0.777141 0.527252

H -1.133823 0.909676 1.599713

C -1.957939 0.045512 -0.204530

C -4.315113 -0.670866 -0.174286

H -4.730256 0.305855 -0.473953

H -4.131487 -1.232079 -1.092260

C -5.315775 -1.405563 0.707151

H -5.499458 -0.856373 1.636854

H -6.272953 -1.518549 0.190901

H -4.941828 -2.400079 0.967794

O -1.897441 -0.197142 -1.450371

O 1.956830 1.222177 1.552551

S 0.292855 3.112899 -0.145848

C 1.928321 3.386646 -0.891728

H 2.036414 4.457171 -1.074274

H 2.719217 3.054880 -0.216374

H 2.007093 2.856649 -1.844280

C 1.805650 -0.740069 0.239437

C 1.087051 -1.517787 -0.680233

C 2.976264 -1.255224 0.809178

C 1.541579 -2.792171 -1.015614

H 0.162289 -1.145954 -1.117148

C 3.430494 -2.526263 0.466417

H 3.523795 -0.643886 1.519335

C 2.712295 -3.298030 -0.447992

H 0.977710 -3.393788 -1.721832

H 4.342282 -2.914069 0.909805

H 3.062823 -4.289803 -0.716834

N -3.040320 -0.520024 0.516874

H -3.149334 -0.097677 1.433297

**Structure 2c_add2**

C -1.411328 -0.758335 0.451206

C -0.226091 -0.149340 0.083022

H -0.217361 0.779557 -0.477063

C 1.081246 -0.805931 0.370782

C 2.095318 0.215872 0.876639

C 3.699247 1.983070 0.310913

H 3.178271 2.759825 0.881390

H 4.463105 1.556722 0.970574

C 4.334978 2.570295 -0.939387

H 3.576725 3.010923 -1.593329

H 5.045420 3.353244 -0.666281

H 4.873997 1.801474 -1.501060

O 2.254808 0.400467 2.088701

O -1.510698 -1.896891 1.031026

H 0.945295 -1.544546 1.165310

S 1.680360 -1.724619 -1.121083

C 3.228417 -2.432043 -0.479941

H 3.028494 -3.050050 0.398246

H 3.659428 -3.056441 -1.264125

H 3.939137 -1.642330 -0.222753

C -2.708473 -0.042208 0.128088

C -3.877420 -0.802430 0.000512

C -2.801593 1.347608 -0.033575

C -5.098655 -0.201434 -0.303570

H -3.807728 -1.875782 0.145336

C -4.021461 1.953845 -0.330989

H -1.918069 1.965769 0.094135

C -5.175970 1.181472 -0.472261

H -5.991291 -0.811859 -0.406662

H -4.072774 3.032930 -0.443611

H -6.125955 1.653983 -0.702826

N 2.750210 0.939723 -0.049007

H 2.623909 0.691167 -1.023346

**Structure 2d**

C -1.860855 -0.480712 -0.001292

C -0.590764 0.307304 -0.000943

H -0.636935 1.393823 0.000610

C 0.590759 -0.307270 -0.002505

H 0.636910 -1.393794 -0.003997

C 1.860849 0.480732 -0.002157

C 4.307986 0.364446 -0.003629

H 4.397556 1.001552 -0.889951

H 4.390850 1.016076 0.872571

C 5.391517 -0.701806 0.009042

H 5.320189 -1.342776 -0.874416

H 6.376341 -0.230733 0.008684

H 5.313474 -1.328166 0.902312

O 1.875148 1.714716 -0.001221

O -1.875155 -1.714660 -0.003279

N 2.988847 -0.254726 -0.002453

H 2.924191 -1.263962 -0.005859

H -2.924203 1.263951 0.002480

N -2.988838 0.254705 0.000702

C -4.307966 -0.364505 0.000833

H -4.395140 -1.007069 -0.881699

H -4.393204 -1.010711 0.880892

C -5.391524 0.701788 0.004218

H -5.317833 1.337311 -0.882951

H -6.376342 0.230705 0.004327

H -5.315868 1.333626 0.893848

**Structure 2d_ts1**

C 1.579449 -0.196323 0.915134

C 0.305914 0.513514 0.542114

C -0.788249 -0.276245 0.165928

H -0.632130 -1.239445 -0.311467

C -2.121546 0.247322 0.263430

C -4.530104 -0.274100 0.112959

H -4.709597 -0.313622 1.197371

H -4.739782 0.750269 -0.205525

C -5.448766 -1.252569 -0.604815

H -5.249074 -2.282276 -0.290546

H -6.493757 -1.029279 -0.376939

H -5.310026 -1.191271 -1.688066

O -2.406993 1.381853 0.713387

O 2.052010 -0.098776 2.053585

H 0.091062 1.336907 1.217142

S 1.100059 1.853383 -1.101752

C 2.658261 2.398791 -0.321885

H 3.363470 1.565957 -0.232692

H 3.124612 3.182452 -0.923319

H 2.467221 2.801354 0.677505

C 3.389327 -1.683786 0.197026

C 3.714207 -2.580379 -0.987745

H 4.197161 -0.959321 0.365071

H 2.925733 -3.322534 -1.140928

N -3.140429 -0.569198 -0.193235

H -2.907974 -1.544016 -0.332080

N 2.134414 -0.982806 -0.030453

H 1.837026 -0.813442 -0.984712

H 3.290828 -2.272746 1.113314

H 3.820577 -1.993614 -1.905403

H 4.654704 -3.107437 -0.813532

**Structure 2d_ts2**

C 2.121496 0.247281 0.263537

C 0.788195 -0.276294 0.166164

H 0.632043 -1.239547 -0.311116

C -0.305965 0.513560 0.542155

C -1.579540 -0.196190 0.915186

C -3.389392 -1.683741 0.197045

H -3.290811 -2.272766 1.113283

H -4.197285 -0.959361 0.365161

C -3.714225 -2.580267 -0.987792

H -2.925709 -3.322367 -1.141033

H -4.654693 -3.107391 -0.813620

H -3.820626 -1.993439 -1.905406

O -2.052057 -0.098671 2.053665

O 2.406960 1.381892 0.713273

H -0.091193 1.337093 1.217052

S -1.099989 1.852975 -1.101988

C -2.657797 2.399111 -0.321894

H -2.466361 2.801895 0.677333

H -3.124064 3.182750 -0.923419

H -3.363216 1.566513 -0.232273

C 4.530060 -0.274235 0.113096

C 5.448702 -1.252458 -0.605039

H 4.739592 0.750225 -0.205179

H 6.493699 -1.029268 -0.377092

N -2.134558 -0.982618 -0.030428

H -1.837162 -0.813270 -0.984684

N 3.140364 -0.569352 -0.192981

H 2.907876 -1.544187 -0.331663

H 4.709716 -0.313998 1.197475

H 5.249001 -2.282276 -0.291137

H 5.309947 -1.190774 -1.688265

**Structure 2d_add1**

C 1.491360 -0.309105 0.842074

C 0.397112 0.630928 0.347774

C -0.771831 -0.130340 -0.153907

H -0.603640 -0.950458 -0.846902

C -2.068265 0.292937 0.140338

C -4.384956 -0.538882 0.237391

H -4.241351 -1.087571 1.183443

H -4.729114 0.462681 0.502165

C -5.427671 -1.249582 -0.614480

H -5.091337 -2.254540 -0.891584

H -6.368570 -1.351155 -0.066831

H -5.620104 -0.689854 -1.534583

O -2.383691 1.258320 0.903809

O 1.588275 -0.600700 2.040299

H 0.093863 1.242558 1.201371

S 1.040878 1.820339 -0.942050

C 2.436355 2.577812 -0.052432

H 3.207960 1.834199 0.164561

H 2.861876 3.354620 -0.689846

H 2.091387 3.032380 0.879228

N -3.132835 -0.402537 -0.497311

H -2.824129 -1.288896 -0.884006

C 3.348965 -1.797957 0.246225

H 2.899133 -2.668322 0.735990

H 4.030782 -1.337749 0.970721

C 4.101722 -2.216531 -1.007094

H 4.569875 -1.352645 -1.488543

H 4.887736 -2.930221 -0.751741

H 3.428547 -2.692275 -1.726253

N 2.298175 -0.849284 -0.090305

H 2.222251 -0.502060 -1.039280

**Structure 2d_add2**

C 2.055596 0.129108 0.360794

C 0.763736 -0.214175 -0.035451

H 0.589512 -1.080368 -0.667752

C -0.395769 0.609194 0.385709

C -1.550915 -0.271031 0.850075

C -3.432968 -1.707464 0.205542

H -3.038339 -2.577744 0.740958

H -4.131900 -1.203156 0.882793

C -4.138900 -2.136902 -1.071051

H -3.449570 -2.657486 -1.742235

H -4.962074 -2.814375 -0.834861

H -4.550902 -1.271860 -1.599388

O -1.715932 -0.525064 2.049535

O 2.380587 1.149697 1.044995

H -0.110194 1.234042 1.236106

S -0.920322 1.781144 -0.968979

C -2.325812 2.623121 -0.176529

H -2.009944 3.092005 0.758379

H -2.683586 3.396670 -0.857993

H -3.139274 1.919832 0.021009

N -2.334142 -0.805626 -0.105424

H -2.198750 -0.491287 -1.059195

H 2.806042 -1.415986 -0.721477

C 4.410054 -0.196128 -0.283654

H 4.364465 0.513812 -1.126728

H 4.726722 0.372885 0.592456

C 5.416931 -1.295406 -0.593106

H 5.106071 -1.879569 -1.466021

H 6.399694 -0.867844 -0.809621

H 5.514957 -1.978580 0.255745

N 3.100324 -0.769839 0.004178

**Structure maleimide**

C -0.666400 1.268488 -0.000161

C 0.666336 1.268481 0.000155

C 1.144111 -0.157311 0.000023

C -1.144125 -0.157279 -0.000081

H -0.000153 -1.954748 -0.000190

H 1.353107 2.103957 0.000218

N -0.000016 -0.942973 -0.000095

O -2.284949 -0.561691 0.000159

O 2.285048 -0.561640 0.000010

H -1.353172 2.103976 -0.000335

**Structure maleimide_ts**

C -0.149348 -0.179377 1.149359

C -1.332339 -0.856683 0.937447

C -2.015889 -0.244899 -0.178317

C -0.162914 1.041975 0.275370

H -1.550360 1.559457 -1.239208

H -1.684478 -1.753504 1.425263

N -1.242833 0.872881 -0.564489

O 0.566752 2.021903 0.319685

O -3.069578 -0.542820 -0.742031

H 0.473578 -0.207335 2.031604

S 1.608118 -1.102023 -0.258390

C 2.986082 0.084468 -0.415220

H 3.747236 -0.076055 0.351958

H 3.455813 -0.010237 -1.397900

H 2.597218 1.104308 -0.319115

**Structure maleimide_add**

C 0.346249 0.087045 0.974072

C -0.766590 -0.899055 0.937692

C -1.754526 -0.472559 0.075196

C -0.156330 1.237814 0.085026

H -1.895200 1.383194 -1.063647

H -0.779361 -1.837368 1.473618

N -1.326793 0.823522 -0.441905

O 0.406825 2.310883 -0.120487

O -2.861750 -0.933000 -0.310136

H 0.615768 0.485695 1.958527

S 1.987994 -0.499611 0.319520

C 1.493700 -0.917727 -1.373862

H 0.557994 -1.482951 -1.342806

H 1.366659 -0.017467 -1.979715

H 2.278171 -1.538159 -1.808719
